# Research on the isolation and identification of black spot disease of *Rosa* *chinensis* in Kunming, China

**DOI:** 10.1038/s41598-023-35295-1

**Published:** 2023-05-23

**Authors:** Yanjie Li, Meiying Pu, Yusi Cui, Ju Gu, Xi Chen, Louqin Wang, Hongzhi Wu, Yuyong Yang, Chao Wang

**Affiliations:** 1 Yunnan Province Engineering Research Center for Functional Flower Resources and Industralization, Southwest Research Center for Landscape Architecture Engineering (State Forestry and Grassland Administration), Yunnan Province South and Southeast Asia Joint R&D Center of Economic Forest Full Industry ChainKey Laboratory of Forest Disaster Warning and Control in Universities of Yunnan Province, Southwest Forestry University, Kunming, 650224 Yunnan China; 2 College of Landscape and Horticulture, Yunnan Agricultural University, Kunming, 650201 China; 3 Kunming Yang Chinese Rose Gardening Co., Ltd, Kunming, 650503 Yunnan China

**Keywords:** Fungi, Pathogens, Plant sciences

## Abstract

Through a survey of rose diseases in the South Tropical Garden in Kunming, China, it was found that black spot was the most common and serious disease of rose cultivated in the open air there, with an incidence of more than 90%. In this study, fungus isolation was performed on leaf samples of five black spot susceptible varieties of rose from the South Tropical Garden by tissue isolation. 18 strains of fungus were initially obtained, and seven of them were finally identified to cause black spot symptoms on healthy leaves of rose after verification by Koch's rule. By observing the morphology of colonies and spores, and constructing a phylogenetic tree by combining molecular biology and multiple genes, two pathogenic fungus were identified, namely, *Alternaria* *alternata* and *Gnomoniopsis* *rosae.* *G.* *rosae* was the first pathogenic fungi of rose black spot isolated and identified in this study. The results of this study can provide a reference base for further research and control of the black spot disease of rose in Kunming.

## Introduction

Rose is one of the most famous and popular of all flowers, China is the birthplace of the *Rosa* *chinensis*.It was spread from the Silk Road to Persia, Ceylon and other countries before the twelfth century, and was highly praised and loved abroad as an endemic species for cultural exchange^1^. In China, rose is defined as the city flower by several cities and is widely used in urban landscaping, but in the process of planting and growing, it is vulnerable to diseases and insects, resulting in poor growth, leaf drop, wilting, and even death, which both affects the ornamental and reduces the economic value of the rose itself^2^.There are ten reported species of rose diseases, of which seven are fungal: powdery mildew, black spot, downy mildew, gray mold, leaf mold, rust, and branch blight, respectively, among them, black spot is the most serious and has become a worldwide disease, which occurs commonly in most of the rose planting areas, especially in open field cultivation with a very high incidence^3^.

Rose black spot was first reported in Sweden in 1815^4^. At present, black spot disease of rose occurs in all parts of world, and has become an important problem to be solved urgently in the production of rose. According to previous studies, there are two species of pathogens that cause black spot in rose: one is *Marssonina* *rosae* and the other is *Alternaria* sp.^5^.Abbas first reported in Pakistan that the pathogenic fungi causing black spot of rose was mainly the *Alternaria* sp.^6^.In 2013, Xu et al. collected 15 leaf samples of rose with typical black spot symptoms in Xi'an, Xianyang, Baoji and Weinan, and identified the pathogenic fungi infesting black spot as *Marssonina* *rosae* after isolation and purification^7^. Feng et al. collected diseased leaves of rose with black spot symptoms in the garden of Yuncheng College, after morphological identification and molecular phylogenetic analysis, the pathogenic fungi causing black spot of rose was *Alternaria* *alternata*^8^.

The aim of this study was to accurately identify two pathogenic fungus isolated from the black spot diseased leaves of five rose varieties in the South Tropical Garden in Kunming, China, through morphological identification and molecular biology phylogenetic analysis combined with pathogenicity determination, which can provide a theoretical basis for subsequent research on the biological characteristics of rose black spot pathogens or for effective control of rose black spot disease in the future.

## Materials and methods

### Experimental materials

In November 2021, We did a rose diseases’ survey of four areas of the South Tropical Garden in Kunming,China, which covering an area about 5 acres. The symptoms of these black spot diseases are similar. Fifty leaf samples of five rose varieties ("Red Leonardo da Vinci", "Sweet Pretty", "Happy Carefree", "Benita" and "Home run") with obvious symptoms of black spot were collected (ten samples were collected for each variety), and brought back to the laboratory for storage at 4 ℃ in the refrigerator. Five rose varieties were severely infected with black spot, and the incidence of each variety was more than 80%.

Medium: potato dextrose agar medium (PDA): potato 200 g, glucose anhydrous 20 g, agar 17–20 g, distilled water 1 L.

Reagents: Sangon Biotech Rapid Fungi Genomic DNA Isolation Kit, PCR primers (ITS1/ITS4, NS1/NS4, LSU1Fd/LR5, EF1-728F/EF1-986R, T1/T2), 2× Taq PCR Mix, nucleic acid dyes, DNA Marker DL2000, 5xTAE, etc.

Instruments: optical microscope, autoclave, constant temperature metal bath, low temperature centrifuge, PCR instrument, gel imaging electrophoresis instrument, constant temperature and humidity incubator, etc.

## Experimental methods

### Isolation and purification of pathogenic fungus

The tissue isolation^9^ was used to isolate rose leaf samples with obvious symptoms of black spot. After rinsing the diseased rose leaves, use a sterilized scalpel to cut 3 × 3 mm pieces of tissue at the junction of diseased and healthy leaves in the ultra-clean bench, then put these tissues into 1% sodium hypochlorite solution (5 s), 75% alcohol (30 s), finally rinse with sterile water three times, place on sterilized filter paper, dried and then inoculate them onto the PDA medium with forceps. Three tissue blocks were inoculated in each Petri dish, marked with number and date on the lid, sealed and incubated in a constant temperature and humidity incubator at 25 ℃. Three groups of parallel controls were used for each experiment. After 2 days, the colonies were grown and immediately purified, after purification 2–3 times to obtain pure culture, inoculate it on PDA test tube slant, put it in 25 ℃ constant temperature incubator to wait for colonies to grow full of medium, put it in the refrigerator at 4 ℃ to be refrigerated.

### Determination of pathogenicity of pathogenic fungus

ex vivo leaves inoculation: healthy leaves of five rose varieties ("Red Leonardo da Vinci", "Sweet Pretty", "Happy Carefree", "Benita" and "Home run") were collected from the South Tropical Garden in Kunming, China in May 2022.The healthy rose leaves were rinsed with tap water, then disinfected with 75% alcohol for 30 s, then rinsed with sterile water three times and placed on sterile filter paper to dry. The fungal blocks were punched at the edge of the colonies with a 5 mm diameter sterilized punch and set aside. Two layers of sterile filter paper were placed in a 9 cm diameter Petri dish and moistened with sterile water, the sterilized healthy rose leaves were placed on the filter paper, one compound leaf was placed in each Petri dish, minimally invasive wounds were made on the small leaves with a sterilized scalpel, then a 5 mm mycelial block was inoculated on the wound and the inoculation site was covered with a sterile wet cotton ball, three small leaves were treated with each compound leaf, and a control inoculation (inoculated with a 5 mm diameter PDA block) was set up, and each experimental group was repeated three times. After inoculation, the petri dishes were sealed with cling film and placed at room temperature, when symptoms appeared on the leaves, they were recorded and photographed immediately.

Inoculation in live leaves: the corresponding varieties of rose cuttings with relatively consistent leaf size were selected, and the surfaces of healthy rose leaves were first disinfected with skimmed cotton balls dipped in 75% alcohol, then the leaves were dipped in sterile water and wiped three times, and the parts to be inoculated were pricked with sterile inoculation needles, then a 5 mm mycelial block was inoculated on the wound and the inoculation site was covered with a sterile wet cotton ball, three replicates were treated for each rose variety, three small leaves were treated for each compound leaf, and set up control inoculation (inoculated with a 5 mm diameter PDA block), and covered the inoculated whole compound leaves with plastic bags, observed and recorded daily and sprayed water to moisture the inoculated leaves. After the symptoms appeared, recorded and photographed immediately.

According to Koch's rule, the tissues of rose leaves at the junction of diseased and healthy were taken for isolation and purification again, the re-isolated strains were compared with the original strains, and the same identification method as the original strains were used to test whether them were consistent with the original pathogenic fungus.

### Morphological identification of pathogenic fungus

The pathogenic fungus of rose black spot were inoculated onto PDA medium and incubated at 25 ℃ for 7–10 days. The colony morphology, color and growth rate were observed and recorded. Slides were prepared by picking mycelium with an inoculating needle, and morphological characteristics such as mycelium, color, spore-producing structure, conidia were observed under the optical microscope and photographed and recorded.

### Molecular biology and phylogenetic identification of pathogenic fungus

The pathogenic fungus were inoculated on PDA medium and incubated for 7 days, after the mycelium had grown all over the dishes, the total genomic DNA of the test strains were extracted and PCR amplification were performed using the fungal universal primers ITS(ITS1/ITS4), SSU(NS1/NS4), LSU(LSU1Fd/LR5), TEF(EF1-728F/EF1-986R) and TUB(T1/T2) (Table 1). The reaction system is shown in Table 2; the PCR amplification reaction procedure is shown in Table 3. The amplified PCR products were detected by electrophoresis on a 1% agarose gel, and if there were single and clear bands, sent it to Biotech Bioengineering Ltd. for sequencing. The sequencing results were analyzed by sequence comparison on the NCBI website using the BLAST tool, and reference to Muhammad Farhan^10^ and Ning Jiang^11^, the known sequences with high homology in GenBank were searched online as model strains. Based on the sequences of multiple gene combinations of rDNAITS, SSU, LSU, TEF1, and LSU, the phylogenetic tree was constructed using the Maximum Likelihood method using MEGA11.0 software to analyze the kinship of the black spot pathogenic strains of rose with the model strains and finally determine their taxonomic status^12^^,^^13^.

**Table 1:** Primers used for PCR amplification of black spot pathogens in rose.

Gene	Primer	Primer sequence	References
ITS	ITS1	5′-TCCGTAGGTGAACCTGCGG-3′	^27^
	ITS4	5′-TCCTCCGCTTATTGATATGC-3′	
SSU	NS1	5′-GTAGTCATATGCTTGTCTC-3′	White et al.
	NS4	5′-CTTCCGTCAATTCCTTTAAG-3′	
LSU	LSU1Fd	5′-GRATCAGGTAGGRATACCCG-3′	^28^
	LR5	5′-TCCTGAGGGAAACTTCG-3′	^29^
TEF1	EF1-728F	5′-CATCGAGAAGTTCGAGAAGG-3′	^30^
	EF1-986R	5′-TACTTGAAGGAACCCTTACC-3′	
TUB	T1	5′-AACATGCGTGAGATTGTAAGT-3′	^31^
	T2	5′-TAGTGACCCTTGGCCCAGTTG-3′

**Table 2:** PCR antithesis system.

ddH_2_O	9.5 μL
2× Taq PCR Mastermix	12.5 mL
Upstream primers(10 μmol/L)	1 μL
Downstream primers(10 μmol/L)	1 μL
DNA Template(100 ng/μL)	1 μL
Total volume	25 μL

**Table 3:** PCR protocol for primer pairs.

Primers	Pre-mutability temperature time (℃) (min)	Mutability	Annealing		Extension			Extension temperature (℃)	^Time^ (min)	Number of cycles
		Temperature (℃)	^Time^ (s)	Temperature (℃)	^Time^ (s)	Temperature (℃)	^Time^ (s)			
ITS1/ITS4	94 4	94	30	61	30	72	90	72	10	35
NS1/NS4	94 5	94	30	48	30	72	90	72	7	35
LSU1Fd/LR5	94 5	94	30	48	30	72	90	72	7	35
EF1-728F/EF1-986R	94 5	94	30	52	30	72	45	72	7	40
T1/T2	95 4	95	30	61	30	72	60	72	10	35

### IUCN policy statement

Experimental studies and field research on plants (cultivated or wild), including the collection of plant material, must comply with relevant institutional, national and international guidelines and legislation. We will strictly adhere to the IUCN Policy Statement on Research on Endangered Species and the Convention on Trade in Endangered Species of Wild Fauna and Flora. All specimens were collected with permission from South Tropical Garden, Kunming.We confirm compliance with the IUCN policy for plant.

## Results

### Symptoms of black spot disease of rose

**Figure 1:**
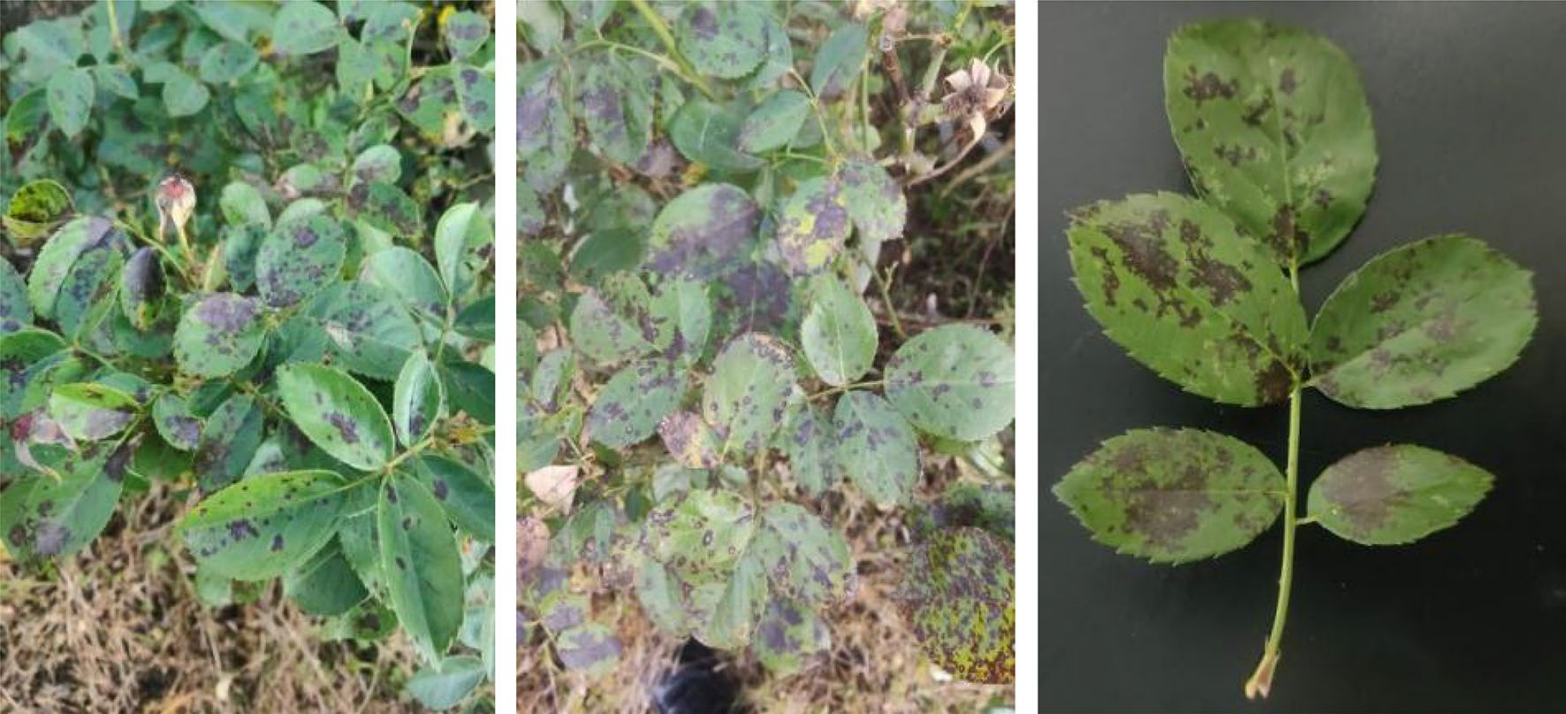
Field symptoms and diseased leaves of black spot of rose (this image was taken by Yanjie Li).

Through the survey on rose disease in the South Tropical Garden in Kunming, China, it was found that black spot was the most widespread and serious, with an incidence reached over 90%. Black spot pathogens mainly infect the leaves of rose (Fig. 1). At the beginning of the disease, brown spots appear on the front side of the leaves, then the spots gradually expand into round or irregular, they are purple-brown to dark brown; late in the disease, the tissue around the spots turns yellow, the lower and middle leaves of the plant all fall off, leaving only the top few leaves.

### Isolation and determination of the pathogenicity of the black spot pathogen fungus of rose

Eighteen fungal strains were isolated and purified from samples of diseased leaves of five different rose species ("Red Leonardo da Vinci"was isolated 4, "Sweet Pretty"was isolated 5, "Happy Carefree"was isolated 4, "Benita"was isolated 2 and "Home run"was isolated 3), these fungus mainly belong to genus of Gnomoniopsis, Alternaria and Nigrospora after DNA extraction, ITS amplification and sequencing, and preliminary comparison at NCBI. All fungus were inoculated separately on the ex vivo healthy rose leaves in the laboratory, and some of leaves started to show symptoms around 5 days, among which the healthy rose leaves inoculated with the strains of *Alternaria* and *Gnomoniopsis* sp. showed the most obvious symptoms (Table 4), while the other strains showed almost no symptoms in the ex vivo leaves after 7 days of inoculation. TMR3-3 (isolated from "Sweet Pretty") of the genus *Gnomoniopsis* and KLWY1-6-2 (isolated from "Happy Carefree") of the genus *Alternaria*, which caused black spot in ex vivo healthy rose leaves were selected as representative strains for inoculation in live leaves, and black spot symptoms appeared on the leaves around 7 days (Table 5). The pathogenic parts of inoculated rose leaves were isolated again, and the same identification method as the original strains was used. The final strains obtained could all correspond to the original strains, so TMR1-1-2, HSDFQ3-7-1, HSDFQ2-4, DDBX2-13, KLWY1-6-2; XP1-6, TMR3-3 are all pathogenic fungus that infest black spot of rose.

**Table 4:** Symptoms of 7-days incidence of pathogenic fungus on healthy moonflower leaves inoculated in vitro.

Strain number	Host	Genus	Inoculation of pathogenic fungus	Front	Back	Average lesion diameter (mm)
TMR1-1-2	"Sweet Pretty"	*Alternaria*				7.1
HSDFQ3-7-1	"Red Leonardo da Vinci"	*Alternaria*				22.1
HSDFQ2-4	"Red Leonardo da Vinci"	*Alternaria*				8.4
KLWY1-6-2	"Happy Carefree"	*Alternaria*				6.9
DDBX2-13	"Benita"	*Alternaria*				26.3
XP1-6	"Home run"	*Gnomoniopsis*				18.6
TMR3-3	"Sweet Pretty"	*Gnomoniopsis*				16.5
ck	—	PDA block				0

**Table 5:** Symptoms of 7d inoculation of healthy leaves with pathogenic fungus in vivo.

Strain number	Host	Genus	Inoculation of pathogenic fungus	Symptoms	Average lesion diameter (mm)
TMR3-3	"Sweet Pretty"	*Gnomoniopsis*			7.1
KLWY1-6-2	"Happy Carefree"	*Alternaria*			7.3
ck	–				0

### Morphological identification of the pathogenic fungus of black spot disease of rose

**Figure 2:**
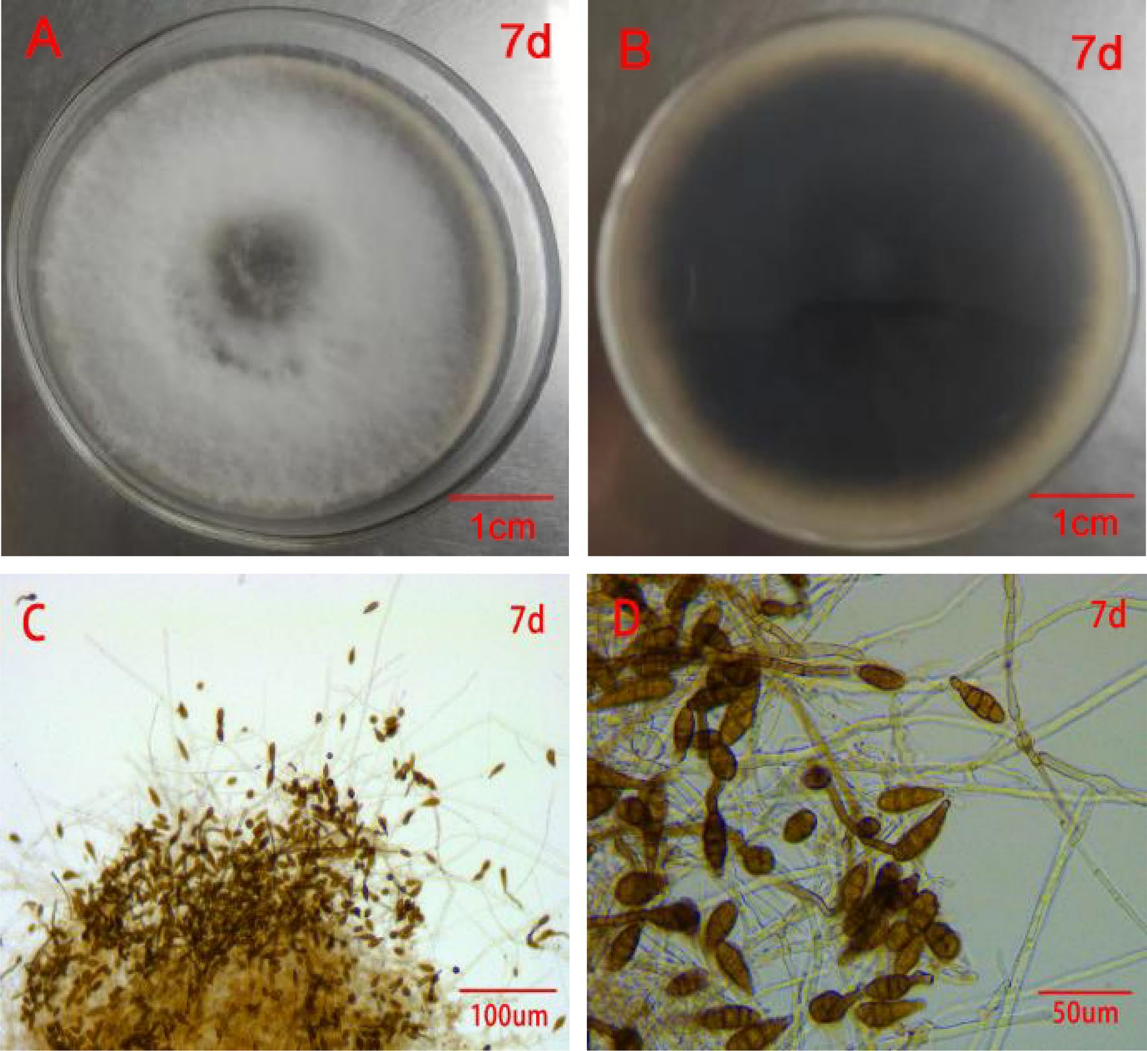
Colony and spore morphology of *Alternaria* *alternata.* *A.* *alternata* grow on PDA medium for 7 days ((**A**) front, (**B**) back). Ascocarp under anatomical microscope was cultured for 7 days (**C,D**).

**Figure 3:**
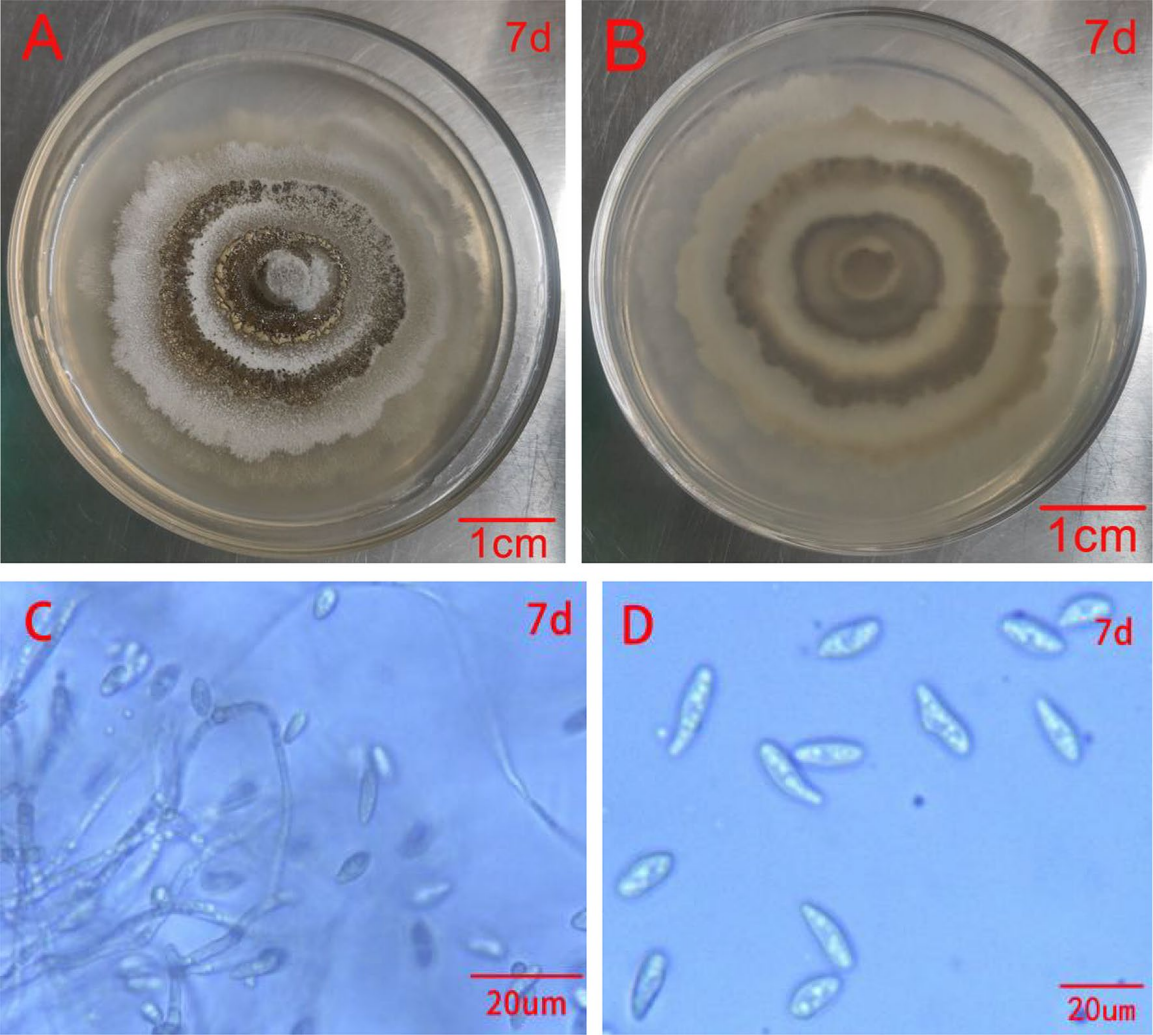
Colony and spore morphology of *Gnomoniopsis* *rosae* *G.* *rosae* grow on PDA medium for 7 days ((**A**) front, (**B**) back). Ascocarp under anatomical microscope was cultured for 7 days(**C,D**).

Five strains: TMR1-1-2 (isolated from "Sweet Pretty"), HSDFQ3-7-1、HSDFQ2-4 (isolated from "Red Leonardo da Vinci"), DDBX2-13 (isolated from "Benita"), KLWY1-6-2 (isolated from "Happy Carefree") of *Alternaria* sp. were incubated on PDA medium at 25 °C for 7–10 days. The morphology and color were consistent, with the initial colonies being grayish white on the front, then gradually turning grayish brown and blackish brown on the back, and the colonies growing over the entire petri dish for about 10 days (Fig. 2A, B). DDBX2-13 was selected as a representative strain and observed under the microscope, the conidia were chained, ovate, inverted pear-shaped or inverted clavate, brownish, with 0–3 longitudinal septa, 2–5 transverse septa, slightly narrowed at the separation, individual with columnar short beak, spore size was (12.1–41.0) μm × (7.0–19.0) μm; conidial peduncle was erect or curved, cespitose, with branches; mycelium was vigorous and velutinous, colorless, non-septate, and about (2.1–3.0) μm in diameter (Fig. 2C, D). These pathogenic fungus were initially identified as the genus Alternaria based on morphological characteristics^14^. Two fungal strains: XP1-6 (isolated from "Home run"), TMR3-3 (isolated from "Sweet Pretty") of the genus *Gnomoniopsis* were cultured at a constant temperature of 25 ℃ in PDA medium, and the morphological characteristics were also consistent. They grew in a ring, the initial mycelium is pure white, then the inner ring of mycelium gradually becomes gray-green, the middle ring is light green, the outermost ring is gray-white; later the colony as a whole is gray-green, the aerial mycelium is sparse, and finally the whole colony is ring-shaped and produces a large number of conidiophores with gray-green to black bases, the conidium is yellowish when it overflows; (Fig. 3A, B); the conidium is fusiform, transparent without septum, the surface is smooth, with droplets, with droplets, spores (7.5–10 × 3.5–4) μm; spore-producing cells are bottle-stemmed or clavate, degenerated from conidial peduncle, transparent, smooth, tip finer than the bottom (Fig. 3C, D).

### Molecular identification and phylogenetic analysis of pathogenic fungus of black spot disease of rose

**Figure 4:**
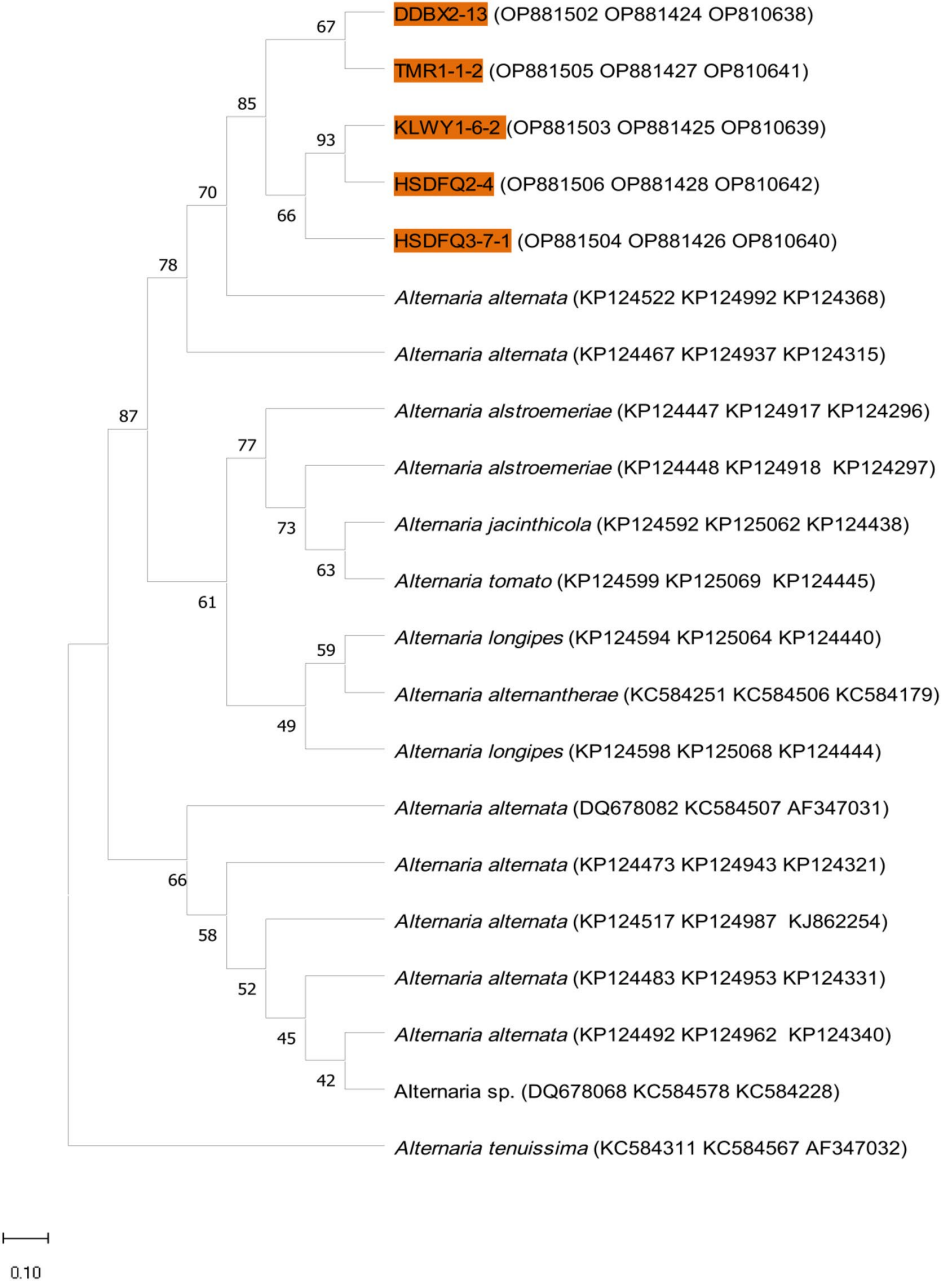
The maximum likelihood phylogenetic tree from combined LSU, SSU and ITS dataset of *Alternaria* *alternata* and its closed related species (the accession numbers of LSU, SSU and ITS genes are shown in parentheses in that order).

**Figure 5:**
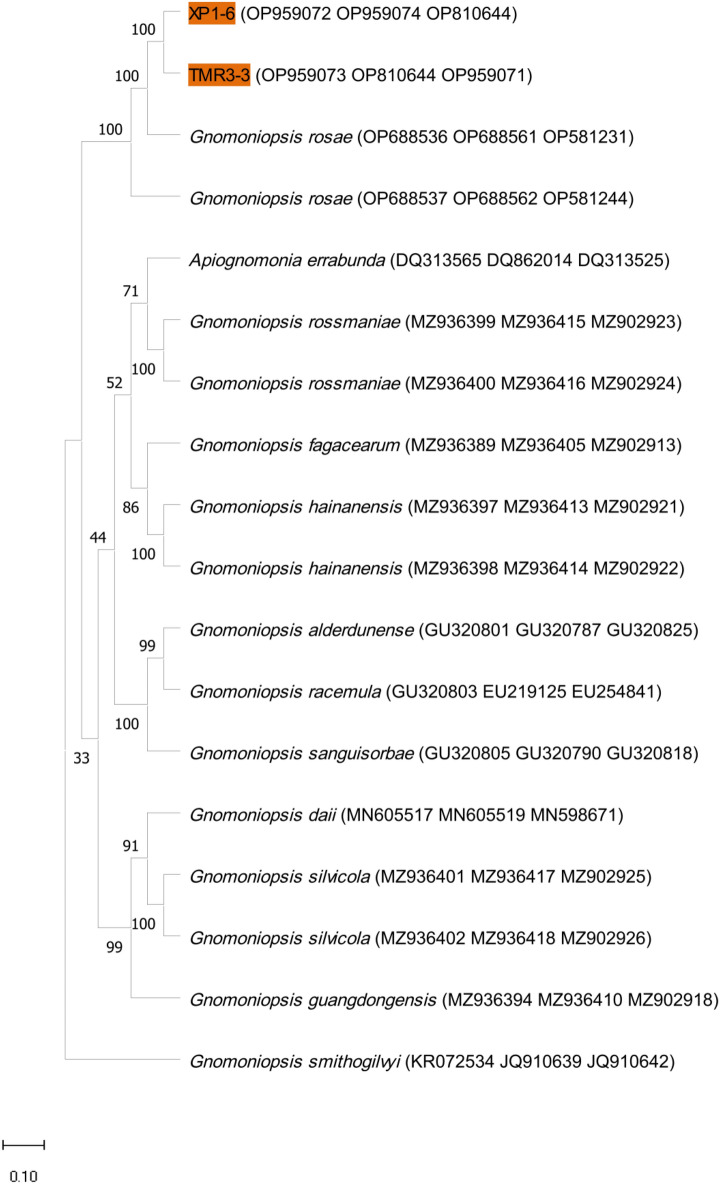
The maximum likelihood phylogenetic tree from combined TEF1, TUB and ITS dataset of *Gnomoniopsis* *rosae* and its closed related species (the accession numbers of TEF1, TUB and ITS genes are shown in parentheses in that order).

Genomic DNA of five strains of the genus *Alternaria* (TMR1-1-2, HSDFQ3-7-1, HSDFQ2-4, DDBX2-13, KLWY1-6-2) were amplified by PCR using the fungal universal primers ITS1/ITS4, NS1/NS4, LSU1Fd/LR5; while two strains of the genus *Gnomoniopsis*(XP1-6, TMR3-3) were amplified using primers of ITS1/ITS4, EF1-728F/EF1-986R and T1/T2. The single and clear bands were obtained after electrophoretic detection on 1% agarose gels. The above PCR amplification products were sent to the Kunming Branch of Beijing DynaScience Biotechnology Co., Ltd. for sequencing, and the sequencing results were uploaded to the NCBI website to obtain the registration numbers (Figs. 4, 5). The phylogenetic tree was constructed using the maximum likelihood method with MEGA11.0 software, and the results showed that the strains: DDBX2-13, KLWY1-6-2, HSDFQ3-7-1, TMR1-1-2 and HSDFQ2-4 clustered in one branch with *Alternaria* *alternata* (Fig. 4); XP1-6, TMR3-3 clustered in one branch with *Gnomoniopsis* *rosae* (Fig. 5). Finally, combined with the morphological characteristics identification, *Alternaria* *alternata* and *Gnomoniopsis* *rosae* were identified as the pathogenic fungus of black spot of rose.

## Discussion and conclusion

In this study, eighteen morphologically distinct fungal strains were isolated from the leaf samples with black spot of five rose varieties("Red Leonardo da Vinci", "Sweet Pretty", "Happy Carefree", "Benita" and "Home run"), through morphological identification, molecular biology identification combined with Koch's rule, a total of seven black spot pathogenic strains were obtained. The mycelium, spore-producing structure, conidia and other structures of these seven pathogenic strains were observed morphologically under a 40× microscope; the pathogenic DNA was extracted and sequenced by PCR amplification using the fungal universal primers ITS, SSU, LSU, TEF1 and TUB, and the phylogenetic tree was constructed by NCBI sequence comparison and downloading the pattern strain multi-gene association in GenBank, and finally TMR1-1-2, HSDFQ3-7-1, HSDFQ2-4, DDBX2-13 and KLWY1-6-2 were identified as *Alternaria* *alternata*; XP1-6 and TMR3-3 were identified as *Gnomoniopsis* *rosae*.

Most results of previous studies on rose black spot disease showed that the pathogenic fungi was *Marssinina* *rosae*, which was different from our research results. Peihong Fang et al. found the pathogenic fungi of rose black spot was *Alternaria* sp, on PDA plates, the colonies started as grayish white and gradually became black or brown, and the conidia were septate, ovate, pear-shaped or rod-shaped^15^, which was consistent with the morphology of *A.* *alternata* in this study. The other pathogenic fungi, *Gnomoniopsis* *rosae*, isolated and identified in this study, has not been reported on rose black spot.The reason for this may be due to the different geographical locations and climatic conditions of different rose varieties cultivated in different regions, so there are also differences in the species of black spot pathogens.

*G.* *rosae* was first reported as a new record species in China by Ning Jiang et al.^16^, who also reported that rose was a new host record for *G.* *rosae*. However, it was different from our research, *G.* *rosae* was isolated from healthy branches of the rose by Ning Jiang et al. and used as a endophytic fungi for subsequent studies on the identification and characterization of species of the genus *Gnomoniopsis*. In this study, *G.* *rosae* was isolated from the leaf samples with black spot of two rose varieties ("Sweet Pretty" and "Home run", which were verified by Koch's rule and identified by morphology and molecular Biology, and finally identified as the pathogenic fungi of rose black spot disease. The hosts of the fungus of the *Gnomoniopsis* spp. are mainly plants of the families *Crustaceae* and *Rosaceae*, and 25 species of *Gnomoniopsis* have been reported, of which ten species are distributed in China, nine species have been reported on plants of the family *Crustaceae*, and one has been reported on *R.* *chinensis* of the family *Rosaceae*^11^. Several species within the *Gnomoniopsis* spp. have been reported as important plant pathogens^17^^,^^18^. *Gnomoniopsis* *daii* has been reported as the causal agent of leaf spot and solid rot of Chinese chestnut^19^^,^^20^, *Gnomoniopsis* *fragariae* caused leaf spot disease of strawberry^21^, *Gnomoniopsis* *chinensis* caused severe ulcer disease of chestnut in Hebei, causing serious economic losses locally^22^, *Gnomoniopsis* *clavulata* caused leaf spot of white oak and red mistletoe in North America^23^, *Gnomoniopsis* *smithogilvyi* caused severe solid rot of European chestnut in Europe, Australia and India^24^^,^^25^^,^^26^.

In this study, a new pathogenic fungi of rose black spot—*Gnomoniopsis* *rosae*, which belongs to the phylum *Ascomycota*, order *Diaporthales*, family *Gnomoniaceae*, and genus *Gnomoniopsis*, was identified for the black spot of rose. This is the first report of *G.* *rosae* causing black spot of *R.* *chinensis*. The identification results can provide a reference basis for further research and control of black spot of rose (Supplementary Information).

## Supplementary Information

Supplementary Information.

## Data Availability

The data that support the findings of this study are openly available in the supplementary material of this article.

## References

[1] Wu LJ (2014). Research on the Culture of Rose (in Chinese).

[2] Lou XY, Wang XY, Pei DL (2021). Identification and biological characteristics of pathogenic bacteria of anthracnose of rose in Shangqiu, Henan (in Chinese). Jiangsu Agric. Sci..

[3] Youren F, Baosheng L, Penghua B (2015). Identification and biological characterization of a novel leaf blight pathogen of rose in Tianjin (in Chinese). Northern Hortic..

[4] Fries, E.M. *Observationes**Mycologicae*. 1–230 (1815).

[5] Zhu JH, Zhang HZ, Chen QIR, Xiong XY, Zhong XH, Li YL (2017). Research progress on the occurrence and damage of black spot disease and resistance breeding of rose (in Chinese). J. Hunan Agric. Univ. (Nat. Sci. Ed.).

[6] Abbas MF, Aziz-ud-Din RK, Qadir A, Rashid A, Qamar MI, Rafique M, Gleason ML (2017). First report of Alternaria black spot of rose caused by *Alternaria*
*alternata* in Pakistan. Plant Dis..

[7] Xu LL, Tao GR, Yao WW (2013). Isolation of endophytic fungi and screening of active strains against black spot disease in rose (in Chinese). Anhui Agric. Sci..

[8] Feng BZ, Li PQ (2019). Identification of pathogenic bacteria and preliminary screening of indoor agents for black spot disease of rose (in Chinese). J. Plant Protect..

[9] Zhang CY (1992). Fungal Diseases of Ornamental Plants (in Chinese).

[10] Farhan, M. *Phylogenetic**Relationships**of**Streptomyces**spp.**Fungi**on**Medicinal**Plants**in**Southwest**China*. 10.27047/d.cnki.ggudu.2021.000611 (Guizhou University, 2021).

[11] Jiang N, Voglmayr H, Bian DR (2021). Morphology and phylogeny of *Gnomoniopsis* (Gnomoniaceae, Diaporthales) from fagaceae leaves in China. J. Fungi.

[12] Xu CN, Zhang HJ, Zhou ZS, Hu TL, Wang ST, Wang YN, Cao KQ (2015). Identification and distribution of Botryosphaeriaceae species associated with blueberry stem blight in China. Eur. J. Plant Pathol..

[13] Zhou Y, Gong GS, Cui YL (2015). Identification of *Botryosphaeriaceae* species causing kiwifruit rot in Sichuan Province, China. Plant Dis..

[14] Wei JC (1979). Handbook of Fungal Identification.

[15] Fang P, Shi S, Liu X, Zhang Z (2020). First report of *Alternaria* black spot of rose caused by *Alternaria*
*alternata* in China. J. Plant Pathol..

[16] Jiang N, Xue H, Piao CG (2022). Characterization and identification of *Gnomoniopsis* species (in Chinese). Terrestrial Ecosyst. Conserv..

[17] Stevanović M, Ristić D, Živković S (2019). Characterization of *Gnomoniopsis*
*idaeicola*, the causal agent of canker and wilting of Blackberry in Serbia. Plant Dis..

[18] Visentin I, Gentile S, Valentino D (2012). *Gnomoniopsis*
*castanea* sp. nov. (Gnomoniaceae, Diaporthales) as the causal agent of nut rot in sweet chestnut. J. Plant Pathol..

[19] Jiang N, Tian CM (2019). An emerging pathogen from rotted chestnut in China: *Gnomoniopsis*
*daii* sp. nov. Forests.

[20] Jiang N, Fan XL, Tian CM (2021). Identification and characterization of leaf-inhabiting fungi from Castanea plantations in China. J. Fungi.

[21] Udayanga D, Miriyagalla SD, Manamgoda DS (2021). Molecular reassessment of *Diaporthalean* fungi associated with strawberry, including the leaf blight fungus, *Paraphomopsis*
*obscurans* gen. et comb. Nov. (Melanconiellaceae). IMA Fungus.

[22] Jiang N, Liang LY, Tian CM (2020). *Gnomoniopsis*
*chinensis* (Gnomoniaceae, Diaporthales): A new fungus causing canker of Chinese chestnut in Hebei Province, China. MycoKeys.

[23] Sogonov MV, Castlebury LA, Rossman AY, White JF (2007). The type species of *Apiognomonia*, *A.*
*veneta*, with its Discula anamorph is distinct from *A.*
*errabunda*. Mycol. Res..

[24] Lione G, Danti R, Fernandez-Conradi P (2019). The emerging pathogen of chestnut *Gnomoniopsis*
*castaneae*: The challenge posed by a versatile fungus. Eur. J. Plant Pathol..

[25] Shuttleworth LA, Walker DM, Guest DI (2016). The chestnut pathogen *Gnomoniopsis*
*smithogilvyi* (Gnomoniaceae, Diaporthales) and its synonyms. Mycotaxon.

[26] Shuttleworth LA, Guest DI (2017). The infection process of chestnut rot, an important disease caused by *Gnomoniopsis*
*smithogilvyi* (Gnomoniaceae, Diaporthales) in Oceania and Europe. Aust. Plant Pathol..

[27] White, T.J., Bruns, T., Lee, S., & Taylor, J. Amplification and direct sequencing of fungal ribosomal RNA genes for phylogenetics. in *PCR**Protocols*. Vol. 2(3). 315–322 (Elsevier, 2022).

[28] Crous PW, Braun U, Wingfield MJ, Wood AR, Shin HD, Summerell BA, Alfenas AC, Cumagun CJR, Groenewald JZ (2009). Phylogeny and taxonomy of obscure genera of microfungi. Persoonia Mol. Phylogeny Evol. Fungi.

[29] Vilgalys R, Hester M (1990). Rapid genetic identification and mapping of enzymatically amplified ribosomal DNA from several *Cryptococcus* species. J. Bacteriol..

[30] Carbone I, Kohn LM (1999). A method for designing primer sets for speciation studies in filamentous ascomycetes Cochliobolu. Mycologia.

[31] O’Donnell K, Nirenberg HI, Aoki T (2000). A Multigene phylogeny of the *Gibberellafujikuroi* species complex: Detection of additional phylogenetically distinct species. Mycoscience.

