# Supplementary material for: Research on the isolation and identification of black spot disease of Rosa chinensis in Kunming, China
Source: Sci Rep. 2023 May 23;13:8299. doi: 10.1038/s41598-023-35295-1 (PMC10206151; doi:10.1038/s41598-023-35295-1)
Supplement: Supplementary file 1 — Supplementary Information. [file 41598_2023_35295_MOESM1_ESM.docx]

Alternaria（ITS）

>DDBX2-13

AATTAATTTAATTCCTTCCGGCTTATGATATGCTTAAGTTCAGCGGGGATCCCTACCTGATCCGAGGTCAAAAGTTGAAAAAAAGGCTTAATGGATGCTAGACCTTTGCTGATAGAGAGTGCGACTTGTGCTGCGCTCCGAAACCAGTAGGCCGGCTGCCAATTACTTTAAGGCGAGTCTCCAGCAAAGCTAGAGACAAGACGCCCAACACCAAGCAAAGCTTGAGGGTACAAATGACGCTCGAACAGGCATGCCCTTTGGAATACCAAAGGGCGCAATGTGCGTTCAAAGATTCGATGATTCACTGAATTCTGCAATTCACACTACTTATCGCATTTCGCTGCGTTCTTCATCGATGCCAGAACCAAGAGATCCGTTGTTGAAAGTTGTAATTATTAATTTGTTACTGACGCTGATTGCAATTACAAAAGGTTTATGTTTGTCCTAGTGGTGGGCGAACCCACCAAGGAAACAAGAAGTACGCAAAAGACAAGGGTGAATAATTCAGCAAGGCTGTAACCCCGAGAGGTTCCAGCCCGCCTTCATATTGAAATAACGATCCCTCCGCAGGCCCCCCTAACGGGAGGGGG

>KLWY1-6-2

AAGAATTTTTTTTTCCTCCGCCTTATTGATATGCTTAAGTTCAGAGGGGAACACTACCTGATCCGAGGTCAAAAGTTGAAAAAAAGGCTTAATGGATGCTAGACCTTTGCTGATAGAGAGTGCGACTTGTGCTGCGCTCCGAAACCAGTAGGCCGGCTGCCAATTACTTTAAGGCGAGTCTCCAGCAAAGCTAGAGACAAGACGCCCAACACCAAGCAAAGCTTGAGGGTACAAATGACGCTCGAACAGGCATGCCCTTTGGAATACCAAAGGGCGCAATGTGCGTTCAAAGATTCGATGATTCACTGAATTCTGCAATTCACACTACTTATCGCATTTCGCTGCGTTCTTCATCGATGCCAGAACCAAGAGATCCGTTGTTGAAAGTTGTAATTATTAATTTGTTACTGACGCTGATTGCAATTACAAAAGGTTTATGTTTGTCCTAGTGGTGGGCGAACCCACCAAGGAAACAAGAAGTACGCAAAAGACAAGGGTGAATAATTCAGCAAGGCTGTAACCCCGAGAGGTTCCAGCCCGCCTCAAAAGGAAACCAATGATCCCTCCGCAGGTCACCCTACGGAGGCGAGAACCATAACC

>HSDFQ3-7-1

TTCCTTCGGCCTTATGATATGCTTAAGTTCACCGGGGATCCCTACCTGATCCGAGGTCAAAAGTTGAAAAAAAGGCTTAATGGATGCTAGACCTTTGCTGATAGAGAGTGCGACTTGTGCTGCGCTCCGAAACCAGTAGGCCGGCTGCCAATTACTTTAAGGCGAGTCTCCAGCAAAGCTAGAGACAAGACGCCCAACACCAAGCAAAGCTTGAGGGTACAAATGACGCTCGAACAGGCATGCCCTTTGGAATACCAAAGGGCGCAATGTGCGTTCAAAGATTCGATGATTCACTGAATTCTGCAATTCACACTACTTATCGCATTTCGCTGCGTTCTTCATCGATGCCAGAACCAAGAGATCCGTTGTTGAAAGTTGTAATTATTAATTTGTTACTGACGCTGATTGCAATTACAAAAGGTTTATGTTTGTCCTAGTGGTGGGCGAACCCACCAAGGAAACAAGAAGTACGCAAAAGACAAGGGTGAATAATTCAGCAAGGCTGTAACCCCGAGAGGTTCCAGCCCGCCTTCATATTTAAGCAACAATCCCTCCGCAGGTCACCCTACGGAGGGGGGATCACTCCCC

>TMR1-1-2

AATCCCCAAATTCCTTCCTCCCGCCTTTTGATATGCTTAAGTTCAGCGGGGATCCCTACCTGATCCGAGGTCAAAAGTTGAAAAAAGGCTTAATGGATGCTAGACCTTTGCTGATAGAGAGTGCGACTTGTGCTGCGCTCCGAAACCAGTAGGCCGGCTGCCAATTACTTTAAGGCGAGTCTCCAGCAAAGCTAGAGACAAGACGCCCAACACCAAGCAAAGCTTGAGGGTACAAATGACGCTCGAACAGGCATGCCCTTTGGAATACCAAAGGGCGCAATGTGCGTTCAAAGATTCGATGATTCACTGAATTCTGCAATTCACACTACTTATCGCATTTCGCTGCGTTCTTCATCGATGCCAGAACCAAGAGATCCGTTGTTGAAAGTTGTAATTATTAATTTGTTACTGACGCTGATTGCAATTACAAAAGGTTTATGTTTGTCCTAGTGGTGGGCGAACCCACCAAGGAAACAAGAAGTACGCAAAAGACAAGGGTGAATAATTCAGCAAGGCTGTAACCCCGAGAGGTTCCAGCCCGCCTTCATATTTGAAGAACGATCCCTCCGCAGGCACCCCTTACGGAAGGGGGGATCCTTTCCTGAT

>HSDFQ2-4

CACATTTTTTTTCCCCCCCCGTTTTGTATGCTTAAGTTCAGCGGGGATCCCTACCTGATCCGAGGTCAAAAGTTGAAAAAAGGCTTAATGGATGCTAGACCTTTGCTGATAGAGAGTGCGACTTGTGCTGCGCTCCGAAACCAGTAGGCCGGCTGCCAATTACTTTAAGGCGAGTCTCCAGCAAAGCTAGAGACAAGACGCCCAACACCAAGCAAAGCTTGAGGGTACAAATGACGCTCGAACAGGCATGCCCTTTGGAATACCAAAGGGCGCAATGTGCGTTCAAAGATTCGATGATTCACTGAATTCTGCAATTCACACTACTTATCGCATTTCGCTGCGTTCTTCATCGATGCCAGAACCAAGAGATCCGTTGTTGAAAGTTGTAATTATTAATTTGTTACTGACGCTGATTGCAATTACAAAAGGTTTATGTTTGTCCTAGTGGTGGGCGAACCCACCAAGGAAACAAGAAGTACGCAAAAGACAAGGGTGAATAATTCAGCAAGGCTGTAACCCCGAGAGGTTCCAGCCCGCCTTCATATGTAGGTAATGATCCCTCCGAGTCCCCCTTTTTGGGGAAAGGGGTTTTTTACCCATTTTTAAGGCGGGCTGGAACCTCTCGGGGTTACGGCCTTGCTGAATTATTCACCCTTGTCTTTTGCGTACTTCTTGTTTCCTTGGTGGGTTCGCCCACCACTAGGACAAACATAAACCTTTTTGTAATTGCAATCAGCGTCAGTAACAAATTAATAATTACAACTTTCAACAACGGGATCTCTTGGGTTCGGGCATCGATTCAAGAACGCAGCGAAAATGCGAATAAGTTAGTGGTGAAATTGCAGAAATTCGGTGAAATCATCGAAATCTTTGGAACGCACATTTGCGCCCCTTTGGGGTATTTCCAAAGGGGCATGGCCTGTTTCGAAGCGTCATTTTGGTACCCCCCAGGCTTTTGGCTTGGGGTGATTGGGGCGGTCTTTGGTCCTCTAGGCTTTTGCCTGGGAGAACTCGCCCTTAAAAGGTAAATTGGGGCAGCCCGGGCCTAACGGGGGTTTTCGGGAAGGCGCAACCCC

>KP124296 Alternaria alstroemeriae

ATCATTACACAAATATGAAGGCGGGCTGGAACCTCTCGGGGTTACAGCCTTGCTGAATTATTCACCCTTGTCTTTTGCGTACTTCTTGTTTCCTTGGTGGGTTCGCCCACCACTAGGACAAACATAAACCTTTTGTAATTGCAATCAGCGTCAGTAACAAATTAATAATTACAACTTTCAACAACGGATCTCTTGGTTCTGGCATCGATGAAGAACGCAGCGAAATGCGATAAGTAGTGTGAATTGCAGAATTCAGTGAATCATCGAATCTTTGAACGCACATTGCGCCCTTTGGTATTCCAAAGGGCATGCCTGTTCGAGCGTCATTTGTACCCTCAAGCTTTGCTTGGTGTTGGGCGTCTTGTCTCTAGCTTTGCTGGAGACTCGCCTTAAAGTAATTGGCAGCCGGCCTACTGGTTTCGGAGCGCAGCACAAGTCGCACTCTCTATCAGCAAAGGTCTAGCATCCATTAAGCCTTTTTTCAACTTTTGACCTCGGATCAGGTAGGGATACC

>KP124297 Alternaria alstroemeriae

ATCATTACACAAATATGAAGGCGGGCTGGAACCTCTCGGGGTTACAGCCTTGCTGAATTATTCACCCTTGTCTTTTGCGTACTTCTTGTTTCCTTGGTGGGTTCGCCCACCACTAGGACAAACATAAACCTTTTGTAATTGCAATCAGCGTCAGTAACAAATTAATAATTACAACTTTCAACAACGGATCTCTTGGTTCTGGCATCGATGAAGAACGCAGCGAAATGCGATAAGTAGTGTGAATTGCAGAATTCAGTGAATCATCGAATCTTTGAACGCACATTGCGCCCTTTGGTATTCCAAAGGGCATGCCTGTTCGAGCGTCATTTGTACCCTCAAGCTTTGCTTGGTGTTGGGCGTCTTGTCTCTAGCTTTGCTGGAGACTCGCCTTAAAGTAATTGGCAGCCGGCCTACTGGTTTCGGAGCGCAGCACAAGTCGCACTCTCTATCAGCAAAGGTCTAGCATCCATTAAGCCTTTTTTCAACTTTTGACCTCGGATCAGGTAGGGATACC

>KC584179 Alternaria alternantherae

ATCATTACACAGATATGAAGGCGGGGCTGGAACCTCTCGGGGTTGCAGTCTTGCTGAATTATTCACCCGTGTCTTTTGCGTACTTCTTGTTTCCTGGGTGGGTTCGCCCACCACCAGGACAAACCATGAACCTTTTGTAATTGCAATCAGCGTCAGTAACAACACAATCATTTACAACTTTCAACAACGGATCTCTTGGTTCTGGCATCGATGAAGAACGCAGCGAAATGCGATAAGTAGTGTGAATTGCAGAATTCAGTGAATCATCGAATCTTTGAACGCACATTGCGCCCTTTGGCATTCCAAAGGGCATGCCTGTTCGAGCGTCATTTGTACCCTCAAGCTTTGCTTGGTGTTGGGCGTCTTTGTCTCTGGCTTTGCTGGAGACTCGCCTTAAAGGAATTGGCAGCCGGCCTACTGGTTTCGGAGCGCAGCACAAGTCGCACTCTCTTCCAGCCACGGTCTGGCATCCATGAAGCCTTTTTTTTTTCAACCTTTGACCTCGGATCAGGTAGGGATACC

>AF347031 Alternaria alternata

AGGGATCATTACACAAATATGAAGGCGGGCTGGAACCTCTCGGGGTTACAGCCTTGCTGAATTATTCACCCTTGTCTTTTGCGTACTTCTTGTTTCCTTGGTGGGTTCGCCCACCACTAGGACAAACATAAACCTTTTGTAATTGCAATCAGCGTCAGTAACAAATTAATAATTACAACTTTCAACAACGGATCTCTTGGTTCTGGCATCGATGAAGAACGCAGCGAAATGCGATAAGTAGTGTGAATTGCAGAATTCAGTGAATCATCGAATCTTTGAACGCACATTGCGCCCTTTGGTATTCCAAAGGGCATGCCTGTTCGAGCGTCATTTGTACCCTCAAGCTTTGCTTGGTGTTGGGCGTCTTGTCTCTAGCTTTGCTGGAGACTCGCCTTAAAGTAATTGGCAGCCGGCCTACTGGTTTCGGAGCGCAGCACAAGTCGCACTCTCTATCAGCAAAGGTCTAGCATCCATTAAGCCTTTTTTTCAACTTTTGACCTCGGATCAGGTAGGGATACCCGCTGAACTTAA

>KC584228 Alternaria sp.

ATCATTACACAAATATGAAGGCGGGCTGGAACCTCTCGGGGTTACAGCCTTGCTGAATTATTCACCCTTGTCTTTTGCGTACTTCTTGTTTCCTTGGTGGGTTCGCCCACCACTAGGACAAACATAAACCTTTTGTAATTGCAATCAGCGTCAGTAACAAATTAATAATTACAACTTTCAACAACGGATCTCTTGGTTCTGGCATCGATGAAGAACGCAGCGAAATGCGATAAGTAGTGTGAATTGCAGAATTCAGTGAATCATCGAATCTTTGAACGCACATTGCGCCCTTTGGTATTCCAAAGGGCATGCCTGTTCGAGCGTCATTTGTACCCTCAAGCTTTGCTTGGTGTTGGGCGTCTTGTCTCTAGCTTTGCTGGAGACTCGCCTTAAAGTAATTGGCAGCCGGCCTACTGGTTTCGGAGCGCAGCACAAGTCGCACTCTCTATCAGCAAAGGTCTAGCATCCATTAAGCCTTTTTTTCAACTTTTGACCTCGGATCAGGTAGGGATACC

>KP124368 Alternaria alternata

ATCATTACACAAATATGAAGGCGGGCTGGAACCTCTCGGGGTTACAGCCTTGCTGAATTATTCACCCTTGTCTTTTGCGTACTTCTTGTTTCCTTGGTGGGTTCGCCCACCACTAGGACAAACATAAACCTTTTGTAATTGCAATCAGCGTCAGTAACAAATTAATAATTACAACTTTCAACAACGGATCTCTTGGTTCTGGCATCGATGAAGAACGCAGCGAAATGCGATAAGTAGTGTGAATTGCAGAATTCAGTGAATCATCGAATCTTTGAACGCACATTGCGCCCTTTGGTATTCCAAAGGGCATGCCTGTTCGAGCGTCATTTGTACCCTCAAGCTTTGCTTGGTGTTGGGCGTCTTGTCTCTAGCTTTGCTGGAGACTCGCCTTAAAGTAATTGGCAGCCGGCCTACTGGTTTCGGAGCGCAGCACAAGTCGCACTCTCTATCAGCAAAGGTCTAGCATCCACTAAGCCTTTTTTTCAACTTTTGACCTCGGATCAGGTAGGGATACC

>KP124438 Alternaria jacinthicola

ATCATTACACAAATATGAAGGCGGGCTGGAATCTCTCGGGGTTACAGCCTTGCTGAATTATTCACCCTTGTCTTTTGCGTACTTCTTGTTTCCTTGGTGGGTTCGCCCACCACTAGGACAAACATAAACCTTTTGTAATTGCAATCAGCGTCAGTAACAAATTAATAATTACAACTTTCAACAACGGATCTCTTGGTTCTGGCATCGATGAAGAACGCAGCGAAATGCGATAAGTAGTGTGAATTGCAGAATTCAGTGAATCATCGAATCTTTGAACGCACATTGCGCCCTTTGGTATTCCAAAGGGCATGCCTGTTCGAGCGTCATTTGTACCCTCAAGCTTTGCTTGGTGTTGGGCGTCTTGTCTCTAGCTTTGCTGGAGACTCGCCTTAAAGTAATTGGCAGCCGGCCTACTGGTTTCGGAGCGCAGCACAAGTCGCACTCTCTATCAGCAAAGGTCTAGCATCCATTAAGCCTTTTTTCAACTTTTGACCTCGGATCAGGTAGGGATACC

>KP124440 Alternaria longipes

ATCATTACACAAATATGAAGGCGGGCTGGAACCTCTCGGGGTTACAGCCTTGCTGAATTATTCACCCTTGTCTTTTGCGTACTTCTTGTTTCCTTGGTGGGTTCGCCCACCACTAGGACAAACATAAACCTTTTGTAATTGCAATCAGCGTCAGTAACAAATTAATAATTACAACTTTCAACAACGGATCTCTTGGTTCTGGCATCGATGAAGAACGCAGCGAAATGCGATAAGTAGTGTGAATTGCAGAATTCAGTGAATCATCGAATCTTTGAACGCACATTGCGCCCTTTGGTATTCCAAAGGGCATGCCTGTTCGAGCGTCATTTGTACCCTCAAGCTTTGCTTGGTGTTGGGCGTCTTGTCTCTAGCTTTGCTGGAGACTCGCCTTAAAGTAATTGGCAGCCGGCCTACTGGTTTCGGAGCGCAGCACAAGTCGCACTCTCTATCAGCAAAGGTCTAGCATCCATTAAGCCTTTTTTCCAACTTTTGACCTCGGATCAGGTAGGGATACC

>KP124444 Alternaria longipes

ATCATTACACAAATATGAAGGCGGGCTGGAACCTCTCGGGGTTACAGCCTTGCTGAATTATTCACCCTTGTCTTTTGCGTACTTCTTGTTTCCTTGGTGGGTTCGCCCACCACTAGGACAAACATAAACCTTTTGTAATTGCAATCAGCGTCAGTAACAAATTAATAATTACAACTTTCAACAACGGATCTCTTGGTTCTGGCATCGATGAAGAACGCAGCGAAATGCGATAAGTAGTGTGAATTGCAGAATTCAGTGAATCATCGAATCTTTGAACGCACATTGCGCCCTTTGGTATTCCAAAGGGCATGCCTGTTCGAGCGTCATTTGTACCCTCAAGCTTTGCTTGGTGTTGGGCGTCTTGTCTCTAGCTTTGCTGGAGACTCGCCTTAAAGTAATTGGCAGCCGGCCTACTGGTTTCGGAGCGCAGCACAAGTCGCACTCTCTATCAGCAAAGGTCTAGCATCCATTAAGCCTTTTTTCCAACTTTTGACCTCGGATCAGGTAGGGATACC

>KJ862254.1 Alternaria palandui isolate CNU131062

TCGTAACAAGGTCTCCGTAGGTGAACCTGCGGAGGGATCATTACACAAATATGAAGGCGGGCTGGAACCTCTCGGGGTTACAGCCTTGCTGAATTATTCACCCTTGTCTTTTGCGTACTTCTTGTTTCCTTGGTGGGTTCGCCCACCACTAGGACAAACATAAACCTTTTGTAATTGCAATCAGCGTCAGTAACAAATTAATAATTACAACTTTCAACAACGGATCTCTTGGTTCTGGCATCGATGAAGAACGCAGCGAAATGCGATAAGTAGTGTGAATTGCAGAATTCAGTGAATCATCGAATCTTTGAACGCACATTGCGCCCTTTGGTATTCCAAAGGGCATGCCTGTTCGAGCGTCATTTGTACCCTCAAGCTTTGCTTGGTGTTGGGCGTCTTGTCTCTAGCTTTGCTGGAGACTCGCCTTAAAGTAATTGGCAGCCGGCCTACTGGTTTCGGAGCGCAGCACAAGTCGCACTCTCTATCAGCAAAGGTCTAGCATCCATTAAGCCTTTTTTTCAACTTTTGACCTCGGATCAGGTAG

>AF347032 Alternaria tenuissima

AGGGATCATTACACAAATATGAAGGCGGGCTGGAACCTCTCGGGGTTACAGCCTTGCTGAATTATTCACCCTTGTCTTTTGCGTACTTCTTGTTTCCTTGGTGGGTTCGCCCACCACTAGGACAAACATAAACCTTTTGTAATTGCAATCAGCGTCAGTAACAAATTAATAATTACAACTTTCAACAACGGATCTCTTGGTTCTGGCATCGATGAAGAACGCAGCGAAATGCGATAAGTAGTGTGAATTGCAGAATTCAGTGAATCATCGAATCTTTGAACGCACATTGCGCCCTTTGGTATTCCAAAGGGCATGCCTGTTCGAGCGTCATTTGTACCCTCAAGCTTTGCTTGGTGTTGGGCGTCTTGTCTCTAGCTTTGCTGGAGACTCGCCTTAAAGTAATTGGCAGCCGGCCTACTGGTTTCGGAGCGCAGCACAAGTCGCACTCTCTATCAGCAAAGGTCTAGCATCCATTAAGCCTTTTTTTCAACTTTTGACCTCGGATCAGGTAGGGATACCCGCTGAACTTAA

>KP124315 Alternaria alternata

ATCATTACACAAATATGAAGGCGGGCTGGAACCTCTCGGGGTTACAGCCTTGCTGAATTATTCACCCTTGTCTTTTGCGTACTTCTTGTTTCCTTGGTGGGTTCGCCCACCACTAGGACAAACATAAACCTTTTGTAATTGCAATCAGCGTCAGTAACAAATTAATAATTACAACTTTCAACAACGGATCTCTTGGTTCTGGCATCGATGAAGAACGCAGCGAAATGCGATAAGTAGTGTGAATTGCAGAATTCAGTGAATCATCGAATCTTTGAACGCACATTGCGCCCTTTGGTATTCCAAAGGGCATGCCTGTTCGAGCGTCATTTGTACCCTCAAGCTTTGCTTGGTGTTGGGCGTCTTGTCTCTAGCTTTGCTGGAGACTCGCCTTAAAGTAATTGGCAGCCGGCCTACTGGTTTCGGAGCGCAGCACAAGTCGCACTCTCTATCAGCAAAGGTCTAGCATCCATTAAGCCTTTTTTTTCAACTTTTGACCTCGGATCAGGTAGGGATACC

>KP124321 Alternaria alternata

ATCATTACACAAATATGAAGGCGGGCTGGAACCTCTCGGGGTTACAGCCTTGCTGAATTATTCACCCTTGTCTTTTGCGTACTTCTTGTTTCCTTGGTGGGTTCGCCCACCACTAGGACAAACATAAACCTTTTGTAATTGCAATCAGCGTCAGTAACAAATTAATAATTACAACTTTCAACAACGGATCTCTTGGTTCTGGCATCGATGAAGAACGCAGCGAAATGCGATAAGTAGTGTGAATTGCAGAATTCAGTGAATCATCGAATCTTTGAACGCACATTGCGCCCTTTGGTATTCCAAAGGGCATGCCTGTTCGAGCGTCATTTGTACCCTCAAGCTTTGCTTGGTGTTGGGCGTCTTGTCTCTAGCTTTGCTGGAGACTCGCCTTAAAGTAATTGGCAGCCGGCCTACTGGTTTCGGAGCGCAGCACAAGTCGCACTCTCTATCAGCAAAGGTCTAGCATCCATTAAGCCTTTTTTTCAACTTTTGACCTCGGATCAGGTAGGGATACC

>KP124340 Alternaria alternata

ATCATTACACAAATATGAAGGCGGGCTGGAACCTCTCGGGGTTACAGCCTTGCTGAATTATTCACCCTTGTCTTTTGCGTACTTCTTGTTTCCTTGGTGGGTTCGCCCACCACTAGGACAAACATAAACCTTTTGTAATTGCAATCAGCGTCAGTAACAAATTAATAATTACAACTTTCAACAACGGATCTCTTGGTTCTGGCATCGATGAAGAACGCAGCGAAATGCGATAAGTAGTGTGAATTGCAGAATTCAGTGAATCATCGAATCTTTGAACGCACATTGCGCCCTTTGGTATTCCAAAGGGCATGCCTGTTCGAGCGTCATTTGTACCCTCAAGCTTTGCTTGGTGTTGGGCGTCTTGTCTCTAGCTTTGCTGGAGACTCGCCTTAAAGTAATTGGCAGCCGGCCTACTGGTTTCGGAGCGCAGCACAAGTCGCACTCTCTATCAGCAAAGGTCTAGCATCCATTAAGCCTTTTTTTCAACTTTTGACCTCGGATCAGGTAGGGATACC

>KP124445 Alternaria tomato

ATCATTACACAAATATGAAGGCGGGCTGGAATCTCTCGGGGTTACAGCCTTGCTGAATTATTCACCCTTGTCTTTTGCGTACTTCTTGTTTCCTTGGTGGGTTCGCCCACCACTAGGACAAACATAAACCTTTTGTAATTGCAATCAGCGTCAGTAACAAATTAATAATTACAACTTTCAACAACGGATCTCTTGGTTCTGGCATCGATGAAGAACGCAGCGAAATGCGATAAGTAGTGTGAATTGCAGAATTCAGTGAATCATCGAATCTTTGAACGCACATTGCGCCCTTTGGTATTCCAAAGGGCATGCCTGTTCGAGCGTCATTTGTACCCTCAAGCTTTGCTTGGTGTTGGGCGTCTTGTCTCTAGCTTTGCTGGAGACTCGCCTTAAAGTAATTGGCAGCCGGCCTACTGGTTTCGGAGCGCAGCACAAGTCGCACTCTCTATCAGCAAAGGTCTAGCATCCATTAAGCCTTTTTTCAACTTTTGACCTCGGATCAGGTAGGGATACC

>KP124331 Alternaria alternata

ATCATTACACAAATATGAAGGCGGGCTGGAACCTCTCGGGGTTACAGCCTTGCTGAATTATTCACCCTTGTCTTTTGCGTACTTCTTGTTTCCTTGGTGGGTTCGCCCACCACTAGGACAAACATAAACCTTTTGTAATTGCAATCAGCGTCAGTAACAAATTAATAATTACAACTTTCAACAACGGATCTCTTGGTTCTGGCATCGATGAAGAACGCAGCGAAATGCGATAAGTAGTGTGAATTGCAGAATTCAGTGAATCATCGAATCTTTGAACGCACATTGCGCCCTTTGGTATTCCAAAGGGCATGCCTGTTCGAGCGTCATTTGTACCCTCAAGCTTTGCTTGGTGTTGGGCGTCTTGTCTCTAGCTTTGCTGGAGACTCGCCTTAAAGTAATTGGCAGCCGGCCTACTGGTTTCGGAGCGCAGCACAAGTCGCACTCTCTATCAGCAAAGGTCTAGCATCCATTAAGCCTTTTTTTCAACTTTTGACCTCGGATCAGGTAGGGATACC

Alternaria（LSU）

>DDBX2-13

GAAAAGAACCCAACAGGGATTGCCCTAGTAACGGCGAGTGAAGCGGCAACAGCTCAAATTTGAAATCTGGCTCTTTTAGAGTCCGAGTTGTAATTTGCAGAGGGCGCTTTGGCTTTGGCAGCGGTCCAAGTTCCTTGGAACAGGACGTCACAGAGGGTGAGAATCCCGTACGTGGTCGCTGGCTATTGCCGTGTAAAGCCCCTTCGACGAGTCGAGTTGTTTGGGAATGCAGCTCTAAATGGGAGGTACATTTCTTCTAAAGCTAAATATTGGCCAGAGACCGATAGCGCACAAGTAGAGTGATCGAAAGATGAAAAGCACTTTGGAAAGAGAGTCAAACAGCACGTGAAATTGTTGAAAGGGAAGCGCTTGCAGCCAGACTTGCTTACAGTTGCTCATCCGGGTTTCTACCCGGTGCACTCTTCTGTAGGCAGGCCAGCATCAGTTTGGGCGGTAGGATAAAGGTCTCTGTCACGTACCTCCTTTCGGGGAGGCCTTATAGGGGAGACGACATACTACCAGCCTGGACTGAGGTCCGCGCATCTGCTAGGATGCTGGCGTAATGGCTGTAAGCGGCCCGTCTTGAAACACGGACCAAGGAGTCTAACATCTATGCGAGTGTTTGGGTGTCAAGCCCGAGCGCGTAATGAAAGTGAACGGAGGTGGGAACCCGCAAGGGTGCACCATCGACCGATCCTGATGTCTTCGGAAGGATTTGAGTAAGAGCATGGCTGTTGGGACCCGAAAGATGGTGAACTATGCTTGAATAGGGTGAAGCCAGAGGAAACTCTGGTGGAGGCTCGCAGCGGTTCTGACGTGCAAATCGATCGTCAAATTTGGGCATAGGGGCGAAAGACTAATCGAA

>KLWY1-6-2

CGATTAGTCTTTCGCCCCTATGCCCAAATTTGACGATCGATTTGCACGTCAGAACCGCTGCGAGCCTCCACCAGAGTTTCCTCTGGCTTCACCCTATTCAAGCATAGTTCACCATCTTTCGGGTCCCAACAGCCATGCTCTTACTCAAATCCTTCCGAAGACATCAGGATCGGTCGATGGTGCACCCTTGCGGGTTCCCACCTCCGTTCACTTTCATTACGCGCTCGGGCTTGACACCCAAACACTCGCATAGATGTTAGACTCCTTGGTCCGTGTTTCAAGACGGGCCGCTTACAGCCATTACGCCAGCATCCTAGCAGATGCGCGGACCTCAGTCCAGGCTGGTAGTATGTCGTCTCCCCTATAAGGCCTCCCCGAAAGGAGGTACGTGACAGAGACCTTTATCCTACCGCCCAAACTGATGCTGGCCTGCCTACAGAAGAGTGCACCGGGTAGAAACCCGGATGAGCAACTGTAAGCAAGTCTGGCTGCAAGCGCTTCCCTTTCAACAATTTCACGTGCTGTTTGACTCTCTTTCCAAAGTGCTTTTCATCTTTCGATCACTCTACTTGTGCGCTATCGGTCTCTGGCCAATATTTAGCTTTAGAAGAAATGTACCTCCCATTTAGAGCTGCATTCCCAAACAACTCGACTCGTCGAAGGGGCTTTACACGGCAATAGCCAGCGACCACGTACGGGATTCTCACCCTCTGTGACGTCCTGTTCCAAGGAACTTGGACCGCTGCCAAAGCCAAAGCGCCCTCTGCAAATTACAACTCGGACTCTAAAAGAGCCAGATTTCAAATTTGAGCTGTTGCCGCTTCACTCGCCGTTACTAGGGCAATCCCTGGTGGGTTCTT

>HSDFQ3-7-1

GAAAAGAACCCACCAGGGATTGCCCTAGTAACGGCGAGTGAAGCGGCAACAGCTCAAATTTGAAATCTGGCTCTTTTAGAGTCCGAGTTGTAATTTGCAGAGGGCGCTTTGGCTTTGGCAGCGGTCCAAGTTCCTTGGAACAGGACGTCACAGAGGGTGAGAATCCCGTACGTGGTCGCTGGCTATTGCCGTGTAAAGCCCCTTCGACGAGTCGAGTTGTTTGGGAATGCAGCTCTAAATGGGAGGTACATTTCTTCTAAAGCTAAATATTGGCCAGAGACCGATAGCGCACAAGTAGAGTGATCGAAAGATGAAAAGCACTTTGGAAAGAGAGTCAAACAGCACGTGAAATTGTTGAAAGGGAAGCGCTTGCAGCCAGACTTGCTTACAGTTGCTCATCCGGGTTTCTACCCGGTGCACTCTTCTGTAGGCAGGCCAGCATCAGTTTGGGCGGTAGGATAAAGGTCTCTGTCACGTACCTCCTTTCGGGGAGGCCTTATAGGGGAGACGACATACTACCAGCCTGGACTGAGGTCCGCGCATCTGCTAGGATGCTGGCGTAATGGCTGTAAGCGGCCCGTCTTGAAACACGGACCAAGGAGTCTAACATCTATGCGAGTGTTTGGGTGTCAAGCCCGAGCGCGTAATGAAAGTGAACGGAGGTGGGAACCCGCAAGGGTGCACCATCGACCGATCCTGATGTCTTCGGAAGGATTTGAGTAAGAGCATGGCTGTTGGGACCCGAAAGATGGTGAACTATGCTTGAATAGGGTGAAGCCAGAGGAAACTCTGGTGGAGGCTCGCAGCGGTTCTGACGTGCAAATCGATCGTCAAATTTGGGCATAGGGGCGAAAGACTAATC

>TMR1-1-2

CAATAAGCGGAGGAAAAGAAACCAACAGGGATTGCCCTAGTAACGGCGAGTGAAGCGGCAACAGCTCAAATTTGAAATCTGGCTCTTTTAGAGTCCGAGTTGTAATTTGCAGAGGGCGCTTTGGCTTTGGCAGCGGTCCAAGTTCCTTGGAACAGGACGTCACAGAGGGTGAGAATCCCGTACGTGGTCGCTGGCTATTGCCGTGTAAAGCCCCTTCGACGAGTCGAGTTGTTTGGGAATGCAGCTCTAAATGGGAGGTACATTTCTTCTAAAGCTAAATATTGGCCAGAGACCGATAGCGCACAAGTAGAGTGATCGAAAGATGAAAAGCACTTTGGAAAGAGAGTCAAACAGCACGTGAAATTGTTGAAAGGGAAGCGCTTGCAGCCAGACTTGCTTGCAGTTGCTCATCCGGGTTTTTACCCGGTGCACTCTTCTGTAGGCAGGCCAGCATCAGTTTGGGCGGTAGGATAAAGGTCTCTGTCACGTACCTCCTTTCGGGGAGGCCTTATAGGGGAGACGACATACTACCAGCCTGGACTGAGGTCCGCGCATCTGCTAGGATGCTGGCGTAATGGCTGTAAGCGGCCCGTCTTGAAACACGGACCAAGGAGTCTAACATCTATGCGAGTGTTTGGGTGTCAAGCCCGAGCGCGTAATGAAAGTGAACGGAGGTGGGAACCCGCAAGGGTGCACCATCGACCGATCCTGATGTCTTCGGAAGGATTTGAGTAAGAGCATGGCTGTTGGGACCCGAAAGATGGTGAACTATGCTTGAATAGGGTGAAGCCAGAGGAAACTCTGGTGGAGGCTCGCAGCGGTTCTGACGTGCAAATCGATCGTCAAATTTGGGCATAGGGGCGAAAGACTAATCG

>HSDFQ2-4

CGATTAGTCTTTCGCCCCTATGCCCAAATTTGACGATCGATTTGCACGTCAGAACCGCTGCGAGCCTCCACCAGAGTTTCCTCTGGCTTCACCCTATTCAAGCATAGTTCACCATCTTTCGGGTCCCAACAGCCATGCTCTTACTCAAATCCTTCCGAAGACATCAGGATCGGTCGATGGTGCACCCTTGCGGGTTCCCACCTCCGTTCACTTTCATTACGCGCTCGGGCTTGACACCCAAACACTCGCATAGATGTTAGACTCCTTGGTCCGTGTTTCAAGACGGGCCGCTTACAGCCATTACGCCAGCATCCTAGCAGATGCGCGGACCTCAGTCCAGGCTGGTAGTATGTCGTCTCCCCTATAAGGCCTCCCCGAAAGGAGGTACGTGACAGAGACCTTTATCCTACCGCCCAAACTGATGCTGGCCTGCCTACAGAAGAGTGCACCGGGTAAAAACCCGGATGAGCAACTGCAAGCAAGTCTGGCTGCAAGCGCTTCCCTTTCAACAATTTCACGTGCTGTTTGACTCTCTTTCCAAAGTGCTTTTCATCTTTCGATCACTCTACTTGTGCGCTATCGGTCTCTGGCCAATATTTAGCTTTAGAAGAAATGTACCTCCCATTTAGAGCTGCATTCCCAAACAACTCGACTCGTCGAAGGGGCTTTACACGGCAATAGCCAGCGACCACGTACGGGATTCTCACCCTCTGTGACGTCCTGTTCCAAGGAACTTGGACCGCTGCCAAAGCCAAAGCGCCCTCTGCAAATTACAACTCGGACTCTAAAAGAGCCAGATTTCAAATTTGAGCTGTTGCCGCTTCACTCGCCGTTACTAGGGCAATCCCTGGTGGGTTCTT

>DQ678082 Alternaria alternata

AGCGGAGGAAAAGAAACCANCAGGGATTGCCCTAGTAACGGCGAGTGAAGCGGCAACAGCTCAAATTTGAAATCTGGCTCTTTTAGAGTCCGAGTTGTAATTTGCAGAGGGCGCTTTGGCTTTGGCAGCGGTCCAAGTTCCTTGGAACAGGACGTCACAGAGGGTGAGAATCCCGTACGTGGTCGCTGGCTATTGCCGTGTAAAGCCCCTTCGACGAGTCGAGTTGTTTGGGAATGCAGCTCTAAATGGGAGGTACATTTCTTCTAAAGCTAAATATTGGCCAGAGACCGATAGCGCACAAGTAGAGTGATCGAAAGATGAAAAGCACTTTGGAAAGAGAGTCAAACAGCACGTGAAATTGTTGAAAGGGAAGCGCTTGCAGCCAGACTTGCTTACAGTTGCTCATCCGGGTTTCTACCCGGTGCACTCTTCTGTAGGCAGGCCAGCATCAGTTTGGGCGGTAGGATAAAGGTCTCTGTCACGTACCTCCTTTCGGGGAGGCCTTATAGGGGAGACGACATACTACCAGCCTGGACTGAGGTCCGCGCATCTGCTAGGATGCTGGCGTAATGGCTGTAAGCGGCCCGTCTTGAAACACGGACCAAGGAGTCTAACATCTATGCGAGTGTTTGGGTGTCAAGCCCGAGCGCGTAATGAAAGTGAACGGAGGTGGGAACCCGCAAGGGTGCACCATCGACCGATCCTGATGTCTTCGGAAGGATTTGAGTAAGAGCATGGCTGTTGGGACCCGAAAGATGGTGAACTATGCTTGAATAGGGTGAAGCCAGAGGAAACTCTGGTGGAGGCTCGCAGCGGTTCTGACGTGCAAATCGATCGTCAAATTTGGGCATAGGGGCGAAAGACTAATCGAACTATCTAGTAGCTGGTTCCTGCCGAAGTTTCCCTCAGGATAGCAGTAACGTATTCAGTTTTATGAGGTAAAGCGAATGATTAGAGGCCTGGGGGTTGAAACAACCTTCACCTATTCTCAAACTTTAAATATGTAAGAAGTCCTTGTTACTTAATTGAACGTGGACAGTTGAATGAAACGTTATTAGTGGGCCATTTTTGGTAAGCAGAACTGGCGATGCGGGATGAACCGAACGAGGGGTTAAAGTGCCGGAATATACGCTCATCAGACACCACAAAAGGTGTTGGTTCATCTAGACAGCAGGACGGTGGCCATGGAAGTCGGAATCCGCTAAGGAGTGTGTAACAACTCACCTGCCGAATGAACTAGCCCTGAAAATGGATGGCGCTCAAGCGTGTTACTTATACCCCTCCGCTGGGGCAAAATTTACGCCCCAGCGAGTAGGCAGGCGTGGAGGTCCGTGACGAA

>KC584311 Alternaria tenuissima

ACAGGGATTGCCCTAGTAACGGCGAGTGAAGCGGCAACAGCTCAAATTTGAAATCTGGCTCTTTTAGAGTCCGAGTTGTAATTTGCAGAGGGCGCTTTGGCTTTGGCAGCGGTCCAAGTTCCTTGGAACAGGACGTCACAGAGGGTGAGAATCCCGTACGTGGTCGCTGGCTATTGCCGTGTAAAGCCCCTTCGACGAGTCGAGTTGTTTGGGAATGCAGCTCTAAATGGGAGGTACATTTCTTCTAAAGCTAAATATTGGCCAGAGACCGATAGCGCACAAGTAGAGTGATCGAAAGATGAAAAGCACTTTGGAAAGAGAGTCAAACAGCACGTGAAATTGTTGAAAGGGAAGCGCTTGCAGCCAGACTTGCTTACAGTTGCTCATCCGGGTTTCTACCCGGTGCACTCTTCTGTAGGCAGGCCAGCATCAGTTTGGGCGGTAGGATAAAGGTCTCTGTCACGTACCTCCTTTCGGGGAGGCCTTATAGGGGAGACGACATACTACCAGCCTGGACTGAGGTCCGCGCATCTGCTAGGATGCTGGCGTAATGGCTGTAAGCGGCCCGTCTTGAAACACGGACCAAGGAGTCTAACATCTATGCGAGTGTTTGGGTGTCAAGCCCGAGCGCGTAATGAAAGTGAACGGAGGTGGGAACCCGCAAGGGTGCACCATCGACCGATCCTGATGTCTTCGGAAGGATTTGAGTAAGAGCATGGCTGTTGGGACCCGAAAGATGGTGAACTATGCTTGAATAGGGTGAAGCCAGAGGAAACTCTGGTGGAGGCTCGCAGCGGTTCTGACGTGCAAATCGATCGTCAAATTTGGGCATAGGGGCGAAAGACTAAT

>KP124522 Alternaria alternata

ACAGGGATTGCCCTAGTAACGGCGAGTGAAGCGGCAACAGCTCAAATTTGAAATCTGGCTCTTTTAGAGTCCGAGTTGTAATTTGCAGAGGGCGCTTTGGCTTTGGCAGCGGTCCAAGTTCCTTGGAACAGGACGTCACAGAGGGTGAGAATCCCGTACGTGGTCGCTGGCTATTGCCGTGTAAAGCCCCTTCGACGAGTCGAGTTGTTTGGGAATGCAGCTCTAAATGGGAGGTACATTTCTTCTAAAGCTAAATATTGGCCAGAGACCGATAGCGCACAAGTAGAGTGATCGAAAGATGAAAAGCACTTTGGAAAGAGAGTCAAACAGCACGTGAAATTGTTGAAAGGGAAGCGCTTGCAGCCAGACTTGCTTACAGTTGCTCATCCGGGTTTCTACCCGGTGCACTCTTCTGTAGGCAGGCCAGCATCAGTTTGGGCGGTAGGATAAAGGTCTCTGTCACGTACCTCCTTTCGGGGAGGCCTTATAGGGGAGACGACATACTACCAGCCTGGACTGAGGTCCGCGCATCTGCTAGGATGCTGGCGTAATGGCTGTAAGCGGCCCGTCTTGAAACACGGACCAAGGAGTCTAACATCTATGCGAGTGTTTGGGTGTCAAGCCCGAGCGCGTAATGAAAGTGAACGGAGGTGGGAACCCGCAAGGGTGCACCATCGACCGATCCTGATGTCTTCGGAAGGATTTGAGTAAGAGCATGGCTGTTGGGACCCGAAAGATGGTGAACTATGCTTGAATAGGGTGAAGCCAGAGGAAACTCTGGTGGAGGCTCGCAGCGGTTCTGACGTGCAAATCGATCGTCAAATTTGGGCATAGGGGCGAAAGACTAAT

>KP124473 Alternaria alternata

ACAGGGATTGCCCTAGTAACGGCGAGTGAAGCGGCAACAGCTCAAATTTGAAATCTGGCTCTTTTAGAGTCCGAGTTGTAATTTGCAGAGGGCGCTTTGGCTTTGGCAGCGGTCCAAGTTCCTTGGAACAGGACGTCACAGAGGGTGAGAATCCCGTACGTGGTCGCTGGCTATTGCCGTGTAAAGCCCCTTCGACGAGTCGAGTTGTTTGGGAATGCAGCTCTAAATGGGAGGTACATTTCTTCTAAAGCTAAATATTGGCCAGAGACCGATAGCGCACAAGTAGAGTGATCGAAAGATGAAAAGCACTTTGGAAAGAGAGTCAAACAGCACGTGAAATTGTTGAAAGGGAAGCGCTTGCAGCCAGACTTGCTTACAGTTGCTCATCCGGGTTTCTACCCGGTGCACTCTTCTGTAGGCAGGCCAGCATCAGTTTGGGCGGTAGGATAAAGGTCTCTGTCACGTACCTCCTTTCGGGGAGGCCTTATAGGGGAGACGACATACTACCAGCCTGGACTGAGGTCCGCGCATCTGCTAGGATGCTGGCGTAATGGCTGTAAGCGGCCCGTCTTGAAACACGGACCAAGGAGTCTAACATCTATGCGAGTGTTTGGGTGTCAAGCCCGAGCGCGTAATGAAAGTGAACGGAGGTGGGAACCCGCAAGGGTGCACCATCGACCGATCCTGATGTCTTCGGAAGGATTTGAGTAAGAGCATGGCTGTTGGGACCCGAAAGATGGTGAACTATGCTTGAATAGGGTGAAGCCAGAGGAAACTCTGGTGGAGGCTCGCAGCGGTTCTGACGTGCAAATCGATCGTCAAATTTGGGCATAGGGGCGAAAGACTAAT

>KP124517 Alternaria alternata

ACAGGGATTGCCCTAGTAACGGCGAGTGAAGCGGCAACAGCTCAAATTTGAAATCTGGCTCTTTTAGAGTCCGAGTTGTAATTTGCAGAGGGCGCTTTGGCTTTGGCAGCGGTCCAAGTTCCTTGGAACAGGACGTCACAGAGGGTGAGAATCCCGTACGTGGTCGCTGGCTATTGCCGTGTAAAGCCCCTTCGACGAGTCGAGTTGTTTGGGAATGCAGCTCTAAATGGGAGGTACATTTCTTCTAAAGCTAAATATTGGCCAGAGACCGATAGCGCACAAGTAGAGTGATCGAAAGATGAAAAGCACTTTGGAAAGAGAGTCAAACAGCACGTGAAATTGTTGAAAGGGAAGCGCTTGCAGCCAGACTTGCTTACAGTTGCTCATCCGGGTTTCTACCCGGTGCACTCTTCTGTAGGCAGGCCAGCATCAGTTTGGGCGGTAGGATAAAGGTCTCTGTCACGTACCTCCTTTCGGGGAGGCCTTATAGGGGAGACGACATACTACCAGCCTGGACTGAGGTCCGCGCATCTGCTAGGATGCTGGCGTAATGGCTGTAAGCGGCCCGTCTTGAAACACGGACCAAGGAGTCTAACATCTATGCGAGTGTTTGGGTGTCAAGCCCGAGCGCGTAATGAAAGTGAACGGAGGTGGGAACCCGCAAGGGTGCACCATCGACCGATCCTGATGTCTTCGGAAGGATTTGAGTAAGAGCATGGCTGTTGGGACCCGAAAGATGGTGAACTATGCTTGAATAGGGTGAAGCCAGAGGAAACTCTGGTGGAGGCTCGCAGCGGTTCTGACGTGCAAATCGATCGTCAAATTTGGGCATAGGGGCGAAAGACTAAT

>KP124483 Alternaria alternata

ACAGGGATTGCCCTAGTAACGGCGAGTGAAGCGGCAACAGCTCAAATTTGAAATCTGGCTCTTTTAGAGTCCGAGTTGTAATTTGCAGAGGGCGCTTTGGCTTTGGCAGCGGTCCAAGTTCCTTGGAACAGGACGTCACAGAGGGTGAGAATCCCGTACGTGGTCGCTGGCTATTGCCGTGTAAAGCCCCTTCGACGAGTCGAGTTGTTTGGGAATGCAGCTCTAAATGGGAGGTACATTTCTTCTAAAGCTAAATATTGGCCAGAGACCGATAGCGCACAAGTAGAGTGATCGAAAGATGAAAAGCACTTTGGAAAGAGAGTCAAACAGCACGTGAAATTGTTGAAAGGGAAGCGCTTGCAGCCAGACTTGCTTACAGTTGCTCATCCGGGTTTCTACCCGGTGCACTCTTCTGTAGGCAGGCCAGCATCAGTTTGGGCGGTAGGATAAAGGTCTCTGTCACGTACCTCCTTTCGGGGAGGCCTTATAGGGGAGACGACATACTACCAGCCTGGACTGAGGTCCGCGCATCTGCTAGGATGCTGGCGTAATGGCTGTAAGCGGCCCGTCTTGAAACACGGACCAAGGAGTCTAACATCTATGCGAGTGTTTGGGTGTCAAGCCCGAGCGCGTAATGAAAGTGAACGGAGGTGGGAACCCGCAAGGGTGCACCATCGACCGATCCTGATGTCTTCGGAAGGATTTGAGTAAGAGCATGGCTGTTGGGACCCGAAAGATGGTGAACTATGCTTGAATAGGGTGAAGCCAGAGGAAACTCTGGTGGAGGCTCGCAGCGGTTCTGACGTGCAAATCGATCGTCAAATTTGGGCATAGGGGCGAAAGACTAAT

>KP124467 Alternaria alternata

ACAGGGATTGCCCTAGTAACGGCGAGTGAAGCGGCAACAGCTCAAATTTGAAATCTGGCTCTTTTAGAGTCCGAGTTGTAATTTGCAGAGGGCGCTTTGGCTTTGGCAGCGGTCCAAGTTCCTTGGAACAGGACGTCACAGAGGGTGAGAATCCCGTACGTGGTCGCTGGCTATTGCCGTGTAAAGCCCCTTCGACGAGTCGAGTTGTTTGGGAATGCAGCTCTAAATGGGAGGTACATTTCTTCTAAAGCTAAATATTGGCCAGAGACCGATAGCGCACAAGTAGAGTGATCGAAAGATGAAAAGCACTTTGGAAAGAGAGTCAAACAGCACGTGAAATTGTTGAAAGGGAAGCGCTTGCAGCCAGACTTGCTTACAGTTGCTCATCCGGGTTTCTACCCGGTGCACTCTTCTGTAGGCAGGCCAGCATCAGTTTGGGCGGTAGGATAAAGGTCTCTGTCACGTACCTCCTTTCGGGGAGGCCTTATAGGGGAGACGACATACTACCAGCCTGGACTGAGGTCCGCGCATCTGCTAGGATGCTGGCGTAATGGCTGTAAGCGGCCCGTCTTGAAACACGGACCAAGGAGTCTAACATCTATGCGAGTGTTTGGGTGTCAAGCCCGAGCGCGTAATGAAAGTGAACGGAGGTGGGAACCCGCAAGGGTGCACCATCGACCGATCCTGATGTCTTCGGAAGGATTTGAGTAAGAGCATGGCTGTTGGGACCCGAAAGATGGTGAACTATGCTTGAATAGGGTGAAGCCAGAGGAAACTCTGGTGGAGGCTCGCAGCGGTTCTGACGTGCAAATCGATCGTCAAATTTGGGCATAGGGGCGAAAGACTAAT

>KP124492 Alternaria alternata

ACAGGGATTGCCCTAGTAACGGCGAGTGAAGCGGCAACAGCTCAAATTTGAAATCTGGCTCTTTTAGAGTCCGAGTTGTAATTTGCAGAGGGCGCTTTGGCTTTGGCAGCGGTCCAAGTTCCTTGGAACAGGACGTCACAGAGGGTGAGAATCCCGTACGTGGTCGCTGGCTATTGCCGTGTAAAGCCCCTTCGACGAGTCGAGTTGTTTGGGAATGCAGCTCTAAATGGGAGGTACATTTCTTCTAAAGCTAAATATTGGCCAGAGACCGATAGCGCACAAGTAGAGTGATCGAAAGATGAAAAGCACTTTGGAAAGAGAGTCAAACAGCACGTGAAATTGTTGAAAGGGAAGCGCTTGCAGCCAGACTTGCTTACAGTTGCTCATCCGGGTTTCTACCCGGTGCACTCTTCTGTAGGCAGGCCAGCATCAGTTTGGGCGGTAGGATAAAGGTCTCTGTCACGTACCTCCTTTCGGGGAGGCCTTATAGGGGAGACGACATACTACCAGCCTGGACTGAGGTCCGCGCATCTGCTAGGATGCTGGCGTAATGGCTGTAAGCGGCCCGTCTTGAAACACGGACCAAGGAGTCTAACATCTATGCGAGTGTTTGGGTGTCAAGCCCGAGCGCGTAATGAAAGTGAACGGAGGTGGGAACCCGCAAGGGTGCACCATCGACCGATCCTGATGTCTTCGGAAGGATTTGAGTAAGAGCATGGCTGTTGGGACCCGAAAGATGGTGAACTATGCTTGAATAGGGTGAAGCCAGAGGAAACTCTGGTGGAGGCTCGCAGCGGTTCTGACGTGCAAATCGATCGTCAAATTTGGGCATAGGGGCGAAAGACTAAT

>DQ678068 Alternaria sp.

TTAAGCATATCAATAAGCGGAGGAAAAGAAACCAACAGGGATTGCCCTAGTAACGGCGAGTGAAGCGGCAACAGCTCAAATTTGAAATCTGGCTCTTTTAGAGTCCGAGTTGTAATTTGCAGAGGGCGCTTTGGCTTTGGCAGCGGTCCAAGTTCCTTGGAACAGGACGTCACAGAGGGTGAGAATCCCGTACGTGGTCGCTGGCTATTGCCGTGTAAAGCCCCTTCGACGAGTCGAGTTGTTTGGGAATGCAGCTCTAAATGGGAGGTACATTTCTTCTAAAGCTAAATATTGGCCAGAGACCGATAGCGCACAAGTAGAGTGATCGAAAGATGAAAAGCACTTTGGAAAGAGAGTCAAACAGCACGTGAAATTGTTGAAAGGGAAGCGCTTGCAGCCAGACTTGCTTACAGTTGCTCATCCGGGTTTCTACCCGGTGCACTCTTCTGTAGGCAGGCCAGCATCAGTTTGGGCGGTAGGATAAAGGTCTCTGTCACGTACCTCCTTTCGGGGAGGCCTTATAGGGGAGACGACATACTACCAGCCTGGACTGAGGTCCGCGCATCTGCTAGGATGCTGGCGTAATGGCTGTAAGCGGCCCGTCTTGAAACACGGACCAAGGAGTCTAACATCTATGCGAGTGTTTGGGTGTCAAGCCCGAGCGCGTAATGAAAGTGAACGGAGGTGGGAACCCGCAAGGGTGCACCATCGACCGATCCTGATGTCTTCGGAAGGATTTGAGTAAGAGCATGGCTGTTGGGACCCGAAAGATGGTGAACTATGCTTGAATAGGGTGAAGCCAGAGGAAACTCTGGTGGAGGCTCGCAGCGGTTCTGACGTGCAAATCGATCGTCAAATTTGGGCATAGGGGCGAAAGACTAATCGAACTATCTAGTAGCTGGTTCCTGCCGAAGTTTCCCTCAGGATAGCAGTAACGTATTCAGTTTTATGAGGTAAAGCGAATGATTAGAGGCCTGGGGGTTGAAACAACCTTCACCTATTCTCAAACTTTAAATATGTAAGAAGTCCTTGTTACTTAATTGAACGTGGACAGTTGAATGAAACGTTATTAGTGGGCCATTTTTGGTAAGCAGAACTGGCGATGCGGGATGAACCGAACGAGGGGTTAAAGTGCCGGAATATACGCTCATCAGACACCACAAAAGGTGTTGGTTCATCTAGACAGCAGGACGGTGGCCATGGAAGTCGGAATCCGCTAAGGAGTGTGTAACAACTCACCTGCCGAATGAACTAGCCCTGAAAATGGATGGCGCTCAAGCGTGTTACTTATACCCCTCCGCTGGGGCAAAATTTACGCCCCA

>KP124447 Alternaria alstroemeriae

ACAGGGATTGCCCTAGTAACGGCGAGTGAAGCGGCAACAGCTCAAATTTGAAATCTGGCTCTTTTAGAGTCCGAGTTGTAATTTGCAGAGGGCGCTTTGGCTTTGGCAGCGGTCCAAGTTCCTTGGAACAGGACGTCACAGAGGGTGAGAATCCCGTACGTGGTCGCTGGCTATTGCCGTGTAAAGCCCCTTCGACGAGTCGAGTTGTTTGGGAATGCAGCTCTAAATGGGAGGTACATTTCTTCTAAAGCTAAATATTGGCCAGAGACCGATAGCGCACAAGTAGAGTGATCGAAAGATGAAAAGCACTTTGGAAAGAGAGTCAAACAGCACGTGAAATTGTTGAAAGGGAAGCGCTTGCAGCCAGACTTGCTTACAGTTGCTCATCCGGGTTTTTACCCGGTGCACTCTTCTGTAGGCAGGCCAGCATCAGTTTGGGCGGTAGGATAAAGGTCTCTGTCACGTACCTCCTTTCGGGGAGGCCTTATAGGGGAGACGACATACTACCAGCCTGGACTGAGGTCCGCGCATCTGCTAGGATGCTGGCGTAATGGCTGTAAGCGGCCCGTCTTGAAACACGGACCAAGGAGTCTAACATCTATGCGAGTGTTTGGGTGTCAAGCCCGAGCGCGTAATGAAAGTGAACGGAGGTGGGAACCCGCAAGGGTGCACCATCGACCGATCCTGATGTCTTCGGAAGGATTTGAGTAAGAGCATGGCTGTTGGGACCCGAAAGATGGTGAACTATGCTTGAATAGGGTGAAGCCAGAGGAAACTCTGGTGGAGGCTCGCAGCGGTTCTGACGTGCAAATCGATCGTCAAATTTGGGCATAGGGGCGAAAGACTAAT

>KP124448 Alternaria alstroemeriae

ACAGGGATTGCCCTAGTAACGGCGAGTGAAGCGGCAACAGCTCAAATTTGAAATCTGGCTCTTTTAGAGTCCGAGTTGTAATTTGCAGAGGGCGCTTTGGCTTTGGCAGCGGTCCAAGTTCCTTGGAACAGGACGTCACAGAGGGTGAGAATCCCGTACGTGGTCGCTGGCTATTGCCGTGTAAAGCCCCTTCGACGAGTCGAGTTGTTTGGGAATGCAGCTCTAAATGGGAGGTACATTTCTTCTAAAGCTAAATATTGGCCAGAGACCGATAGCGCACAAGTAGAGTGATCGAAAGATGAAAAGCACTTTGGAAAGAGAGTCAAACAGCACGTGAAATTGTTGAAAGGGAAGCGCTTGCAGCCAGACTTGCTTACAGTTGCTCATCCGGGTTTTTACCCGGTGCACTCTTCTGTAGGCAGGCCAGCATCAGTTTGGGCGGTAGGATAAAGGTCTCTGTCACGTACCTCCTTTCGGGGAGGCCTTATAGGGGAGACGACATACTACCAGCCTGGACTGAGGTCCGCGCATCTGCTAGGATGCTGGCGTAATGGCTGTAAGCGGCCCGTCTTGAAACACGGACCAAGGAGTCTAACATCTATGCGAGTGTTTGGGTGTCAAGCCCGAGCGCGTAATGAAAGTGAACGGAGGTGGGAACCCGCAAGGGTGCACCATCGACCGATCCTGATGTCTTCGGAAGGATTTGAGTAAGAGCATGGCTGTTGGGACCCGAAAGATGGTGAACTATGCTTGAATAGGGTGAAGCCAGAGGAAACTCTGGTGGAGGCTCGCAGCGGTTCTGACGTGCAAATCGATCGTCAAATTTGGGCATAGGGGCGAAAGACTAAT

>KP124592 Alternaria jacinthicola

ACAGGGATTGCCCTAGTAACGGCGAGTGAAGCGGCAACAGCTCAAATTTGAAATCTGGCTCTTTTAGAGTCCGAGTTGTAATTTGCAGAGGGCGCTTTGGCTTTGGCAGCGGTCCAAGTTCCTTGGAACAGGACGTCACAGAGGGTGAGAATCCCGTACGTGGTCGCTGGCTATTGCCGTGTAAAGCCCCTTCGACGAGTCGAGTTGTTTGGGAATGCAGCTCTAAATGGGAGGTACATTTCTTCTAAAGCTAAATATTGGCCAGAGACCGATAGCGCACAAGTAGAGTGATCGAAAGATGAAAAGCACTTTGGAAAGAGAGTCAAACAGCACGTGAAATTGTTGAAAGGGAAGCGCTTGCAGCCAGACTTGCTTACAGTTGCTCATCCGGGTTTTTACCCGGTGCACTCTTCTGTAGGCAGGCCAGCATCAGTTTGGGCGGTAGGATAAAGGTCTCTGTCACGTACCTCCTTTCGGGGAGGCCTTATAGGGGAGACGACATACTACCAGCCTGGACTGAGGTCCGCGCATCTGCTAGGATGCTGGCGTAATGGCTGTAAGCGGCCCGTCTTGAAACACGGACCAAGGAGTCTAACATCTATGCGAGTGTTTGGGTGTCAAGCCCGAGCGCGTAATGAAAGTGAACGGAGGTGGGAACCCGCAAGGGTGCACCATCGACCGATCCTGATGTCTTCGGAAGGATTTGAGTAAGAGCATGGCTGTTGGGACCCGAAAGATGGTGAACTATGCTTGAATAGGGTGAAGCCAGAGGAAACTCTGGTGGAGGCTCGCAGCGGTTCTGACGTGCAAATCGATCGTCAAATTTGGGCATAGGGGCGAAAGACTAAT

>KP124599 Alternaria tomato

ACAGGGATTGCCCTAGTAACGGCGAGTGAAGCGGCAACAGCTCAAATTTGAAATCTGGCTCTTTTAGAGTCCGAGTTGTAATTTGCAGAGGGCGCTTTGGCTTTGGCAGCGGTCCAAGTTCCTTGGAACAGGACGTCACAGAGGGTGAGAATCCCGTACGTGGTCGCTGGCTATTGCCGTGTAAAGCCCCTTCGACGAGTCGAGTTGTTTGGGAATGCAGCTCTAAATGGGAGGTACATTTCTTCTAAAGCTAAATATTGGCCAGAGACCGATAGCGCACAAGTAGAGTGATCGAAAGATGAAAAGCACTTTGGAAAGAGAGTCAAACAGCACGTGAAATTGTTGAAAGGGAAGCGCTTGCAGCCAGACTTGCTTACAGTTGCTCATCCGGGTTTTTACCCGGTGCACTCTTCTGTAGGCAGGCCAGCATCAGTTTGGGCGGTAGGATAAAGGTCTCTGTCACGTACCTCCTTTCGGGGAGGCCTTATAGGGGAGACGACATACTACCAGCCTGGACTGAGGTCCGCGCATCTGCTAGGATGCTGGCGTAATGGCTGTAAGCGGCCCGTCTTGAAACACGGACCAAGGAGTCTAACATCTATGCGAGTGTTTGGGTGTCAAGCCCGAGCGCGTAATGAAAGTGAACGGAGGTGGGAACCCGCAAGGGTGCACCATCGACCGATCCTGATGTCTTCGGAAGGATTTGAGTAAGAGCATGGCTGTTGGGACCCGAAAGATGGTGAACTATGCTTGAATAGGGTGAAGCCAGAGGAAACTCTGGTGGAGGCTCGCAGCGGTTCTGACGTGCAAATCGATCGTCAAATTTGGGCATAGGGGCGAAAGACTAAT

>KP124594 Alternaria longipes

ACAGGGATTGCCCTAGTAACGGCGAGTGAAGCGGCAACAGCTCAAATTTGAAATCTGGCTCTTTTAGAGTCCGAGTTGTAATTTGCAGAGGGCGCTTTGGCTTTGGCAGCGGTCCAAGTTCCTTGGAACAGGACGTCACAGAGGGTGAGAATCCCGTACGTGGTCGCTGGCTATTGCCGTGTAAAGCCCCTTCGACGAGTCGAGTTGTTTGGGAATGCAGCTCTAAATGGGAGGTACATTTCTTCTAAAGCTAAATATTGGCCAGAGACCGATAGCGCACAAGTAGAGTGATCGAAAGATGAAAAGCACTTTGGAAAGAGAGTCAAACAGCACGTGAAATTGTTGAAAGGGAAGCGCTTGCAGCCAGACTTGCTTGCAGTTGCTCATCCGGGTTTTTACCCGGTGCACTCTTCTGTAGGCAGGCCAGCATCAGTTTGGGCGGTAGGATAAAGGTCTCTGTCACGTACCTCCTTTCGGGGAGGCCTTATAGGGGAGACGACATACTACCAGCCTGGACTGAGGTCCGCGCATCTGCTAGGATGCTGGCGTAATGGCTGTAAGCGGCCCGTCTTGAAACACGGACCAAGGAGTCTAACATCTATGCGAGTGTTTGGGTGTCAAGCCCGAGCGCGTAATGAAAGTGAACGGAGGTGGGAACCCGCAAGGGTGCACCATCGACCGATCCTGATGTCTTCGGAAGGATTTGAGTAAGAGCATGGCTGTTGGGACCCGAAAGATGGTGAACTATGCTTGAATAGGGTGAAGCCAGAGGAAACTCTGGTGGAGGCTCGCAGCGGTTCTGACGTGCAAATCGATCGTCAAATTTGGGCATAGGGGCGAAAGACTAAT

>KP124598 Alternaria longipes

ACAGGGATTGCCCTAGTAACGGCGAGTGAAGCGGCAACAGCTCAAATTTGAAATCTGGCTCTTTTAGAGTCCGAGTTGTAATTTGCAGAGGGCGCTTTGGCTTTGGCAGCGGTCCAAGTTCCTTGGAACAGGACGTCACAGAGGGTGAGAATCCCGTACGTGGTCGCTGGCTATTGCCGTGTAAAGCCCCTTCGACGAGTCGAGTTGTTTGGGAATGCAGCTCTAAATGGGAGGTACATTTCTTCTAAAGCTAAATATTGGCCAGAGACCGATAGCGCACAAGTAGAGTGATCGAAAGATGAAAAGCACTTTGGAAAGAGAGTCAAACAGCACGTGAAATTGTTGAAAGGGAAGCGCTTGCAGCCAGACTTGCTTGCAGTTGCTCATCCGGGTTTTTACCCGGTGCACTCTTCTGTAGGCAGGCCAGCATCAGTTTGGGCGGTAGGATAAAGGTCTCTGTCACGTACCTCCTTTCGGGGAGGCCTTATAGGGGAGACGACATACTACCAGCCTGGACTGAGGTCCGCGCATCTGCTAGGATGCTGGCGTAATGGCTGTAAGCGGCCCGTCTTGAAACACGGACCAAGGAGTCTAACATCTATGCGAGTGTTTGGGTGTCAAGCCCGAGCGCGTAATGAAAGTGAACGGAGGTGGGAACCCGCAAGGGTGCACCATCGACCGATCCTGATGTCTTCGGAAGGATTTGAGTAAGAGCATGGCTGTTGGGACCCGAAAGATGGTGAACTATGCTTGAATAGGGTGAAGCCAGAGGAAACTCTGGTGGAGGCTCGCAGCGGTTCTGACGTGCAAATCGATCGTCAAATTTGGGCATAGGGGCGAAAGACTAAT

>KC584251 Alternaria alternantherae

ACAGGGATTGCCCTAGTAACGGCGAGTGAAGCGGCAACAGCTCAAATTTGAAATCTGGCTCTTTTAGAGTCCGAGTTGTAATTTGCAGAGGGCGCTTTGGCTTTGGCAGCGGTCCAAGTTCCTTGGAACAGGACGTCACAGAGGGTGAGAATCCCGTACGTGGTCGCTGGCTATTGCCGTGTAAAGCCCCTTCGACGAGTCGAGTTGTTTGGGAATGCAGCTCTAAATGGGAGGTACATTTCTTCTAAAGCTAAATATTGGCCAGAGACCGATAGCGCACAAGTAGAGTGATCGAAAGATGAAAAGCACTTTGGAAAGAGAGTCAAACAGCACGTGAAATTGTTGAAAGGGAAGCGCTTGCAGCCAGACTTGCTTGCAGTTGCTCATCCGGGCTTTTGCCCGGTGCACTCTTCTGTAGGCAGGCCAGCATCAGTTTGGGCGGTAGGATAAAGGTCTCTGTCACGTACCTCCTTTCGGGGAGGCCTTATAGGGGAGGCGACATACTACCAGCCTGGACTGAGGTCCGCGCATCTGCTAGGATGCTGGCGTAATGGCTGTAAGCGGCCCGTCTTGAAACACGGACCAAGGAGTCTAACATCTATGCGAGTGTTTGGGTGTCAAGCCCGAGCGCGTAATGAAAGTGAACGGAGGTGGGAACCCGCAAGGGTGCACCATCGACCGATCCTGATGTCTTCGGAAGGATTTGAGTAAGAGCATGGCTGTTGGGACCCGAAAGATGGTGAACTATGCTTGAATAGGGTGAAGCCAGAGGAAACTCTGGTGGAGGCTCGCAGCGGTTCTGACGTGCAAATCGATCGTCAAATTTGGGCATAGGGGCGAAAGACTAAT

Alternaria（SSU）

>DDBX2-13

TTATACCGTGAAACTGCGAATGGCTCATTAAATCAGTTATCGTTTATTTGATAATACCTTACTACTTGGATAACCGTGGTAATTCTAGAGCTAATACATGCTGAAAATCCCGACTTCGGAAGGGATGTGTTTATTAGATAAAAAACCAATGCCCTTCGGGGCTTTTTGGTGATTCATGATAACTTTACGGATCGCATAGCCTTGCGCTGGCGACGGTTCATTCAAATTTCTGCCCTATCAACTTTCGATGGTAAGGTATTGGCTTACCATGGTTTCAACGGGTAACGGGGAATTAGGGTTCGATTCCGGAGAGGGAGCCTGAGAAACGGCTACCACATCCAAGGAAGGCAGCAGGCGCGCAAATTACCCAATCCCGACACGGGGAGGTAGTGACAATAAATACTGATACAGGGCTCTTTTGGGTCTTGTAATTGGAATGAGTACAATTTAAACCTCTTAACGAGGAACAATTGGAGGGCAAGTCTGGTGCCAGCAGCCGCGGTAATTCCAGCTCCAATAGCGTATATTAAAGTTGTTGCAGTTAAAAAGCTCGTAGTTGAAACTTGGGCCTGGCTGGCGGGTCCGCCTCACCGCGTGCACTCGTCCGGCCGGGCCTTCCTTCTGAAGAACCTCATGCCCTTCACTGGGCGTGCTGGGGAATCAGGACTTTTACTTTGAAAAAATTAGAGTGTTCAAAGCAGGCCTTTGCTCGAATACGTTAGCATGGAATAATAAAATAGGGCGTGCGTTTCTATTTTGTTGGTTTCTAGAGACGCCGCAATGATTAACAGGAACAGTCGGGGGCATCAGTATTCAGTTGTCAGAGGTGAAATTCTTGGATTTACTGAAGACTAACTACTGCGAAAGCATTTGCCAAGGATGTTTTCATTAATCAGTGAACGAAAGTTAGGGGATCGAAGACGATCAGATACCGTCGTAGTCTTAACCGTAAACTATGCCGACTAGGGATCGGGCGATGTTCTTTTTCTGACTCGCTCGGCACCTTACGAGAAATCAAAGTTTTT

>KLWY1-6-2

ATACCGTGAAACTGCGAATGGCTCATTAAATCAGTTATCGTTTATTTGATAATACCTTACTACTTGGATAACCGTGGTAATTCTAGAGCTAATACATGCTGAAAATCCCGACTTCGGAAGGGATGTGTTTATTAGATAAAAAACCAATGCCCTTCGGGGCTTTTTGGTGATTCATGATAACTTTACGGATCGCATAGCCTTGCGCTGGCGACGGTTCATTCAAATTTCTGCCCTATCAACTTTCGATGGTAAGGTATTGGCTTACCATGGTTTCAACGGGTAACGGGGAATTAGGGTTCGATTCCGGAGAGGGAGCCTGAGAAACGGCTACCACATCCAAGGAAGGCAGCAGGCGCGCAAATTACCCAATCCCGACACGGGGAGGTAGTGACAATAAATACTGATACAGGGCTCTTTTGGGTCTTGTAATTGGAATGAGTACAATTTAAACCTCTTAACGAGGAACAATTGGAGGGCAAGTCTGGTGCCAGCAGCCGCGGTAATTCCAGCTCCAATAGCGTATATTAAAGTTGTTGCAGTTAAAAAGCTCGTAGTTGAAACTTGGGCCTGGCTGGCGGGTCCGCCTCACCGCGTGCACTCGTCCGGCCGGGCCTTCCTTCTGAAGAACCTCATGCCCTTCACTGGGCGTGCTGGGGAATCAGGACTTTTACTTTGAAAAAATTAGAGTGTTCAAAGCAGGCCTTTGCTCGAATACGTTAGCATGGAATAATAAAATAGGGCGTGCGTTTCTATTTTGTTGGTTTCTAGAGACGCCGCAATGATTAACAGGAACAGTCGGGGGCATCAGTATTCAGTTGTCAGAGGTGAAATTCTTGGATTTACTGAAGACTAACTACTGCGAAAGCATTTGCCAAGGATGTTTTCATTAATCAGTGAACGAAAGTTAGGGGATCGAAGACGATCAGATACCGTCGTAGTCTTAACCGTAAACTATGCCGACTAGGGATCGGGCGATGTTCTTTTTCTGACTCGCTCGGCACCTTACGAGAAATCAAAGTTTTTGGGTT

>HSDFQ3-7-1

TTATACCGTGAAACTGCGAATGGCTCATTAAATCAGTTATCGTTTATTTGATAATACCTTACTACTTGGATAACCGTGGTAATTCTAGAGCTAATACATGCTGAAAATCCCGACTTCGGAAGGGATGTGTTTATTAGATAAAAAACCAATGCCCTTCGGGGCTTTTTGGTGATTCATGATAACTTTACGGATCGCATAGCCTTGCGCTGGCGACGGTTCATTCAAATTTCTGCCCTATCAACTTTCGATGGTAAGGTATTGGCTTACCATGGTTTCAACGGGTAACGGGGAATTAGGGTTCGATTCCGGAGAGGGAGCCTGAGAAACGGCTACCACATCCAAGGAAGGCAGCAGGCGCGCAAATTACCCAATCCCGACACGGGGAGGTAGTGACAATAAATACTGATACAGGGCTCTTTTGGGTCTTGTAATTGGAATGAGTACAATTTAAACCTCTTAACGAGGAACAATTGGAGGGCAAGTCTGGTGCCAGCAGCCGCGGTAATTCCAGCTCCAATAGCGTATATTAAAGTTGTTGCAGTTAAAAAGCTCGTAGTTGAAACTTGGGCCTGGCTGGCGGGTCCGCCTCACCGCGTGCACTCGTCCGGCCGGGCCTTCCTTCTGAAGAACCTCATGCCCTTCACTGGGCGTGCTGGGGAATCAGGACTTTTACTTTGAAAAAATTAGAGTGTTCAAAGCAGGCCTTTGCTCGAATACGTTAGCATGGAATAATAAAATAGGGCGTGCGTTTCTATTTTGTTGGTTTCTAGAGACGCCGCAATGATTAACAGGAACAGTCGGGGGCATCAGTATTCAGTTGTCAGAGGTGAAATTCTTGGATTTACTGAAGACTAACTACTGCGAAAGCATTTGCCAAGGATGTTTTCATTAATCAGTGAACGAAAGTTAGGGGATCGAAGACGATCAGATACCGTCGTAGTCTTAACCGTAAACTATGCCGACTAGGGATCGGGCGATGTTCTTTTTCTGACTCGCTCGGCACCTTACGAGAAATCAAAGTTTTTGGGTTCTGGGGGG

>TMR1-1-2

GAACCCAAAAACTTTGATTTCTCGTAAGGTGCCGAGCGAGTCAGAAAAAGAACATCGCCCGATCCCTAGTCGGCATAGTTTACGGTTAAGACTACGACGGTATCTGATCGTCTTCGATCCCCTAACTTTCGTTCACTGATTAATGAAAACATCCTTGGCAAATGCTTTCGCAGTAGTTAGTCTTCAGTAAATCCAAGAATTTCACCTCTGACAACTGAATACTGATGCCCCCGACTGTTCCTGTTAATCATTGCGGCGTCTCTAGAAACCAACAAAATAGAAACGCACGCCCTATTTTATTATTCCATGCTAACGTATTCGAGCAAAGGCCTGCTTTGAACACTCTAATTTTTTCAAAGTAAAAGTCCTGATTCCCCAGCACGCCCAGTGAAGGGCATGAGGTTCTTCAGAAGGAAGGCCCGGCCGGACGAGTGCACGCGGTGAGGCGGACCCGCCAGCCAGGCCCAAGTTTCAACTACGAGCTTTTTAACTGCAACAACTTTAATATACGCTATTGGAGCTGGAATTACCGCGGCTGCTGGCACCAGACTTGCCCTCCAATTGTTCCTCGTTAAGAGGTTTAAATTGTACTCATTCCAATTACAAGACCCAAAAGAGCCCTGTATCAGTATTTATTGTCACTACCTCCCCGTGTCGGGATTGGGTAATTTGCGCGCCTGCTGCCTTCCTTGGATGTGGTAGCCGTTTCTCAGGCTCCCTCTCCGGAATCGAACCCTAATTCCCCGTTACCCGTTGAAACCATGGTAAGCCAATACCTTACCATCGAAAGTTGATAGGGCAGAAATTTGAATGAACCGTCGCCAGCGCAAGGCTATGCGATCCGTAAAGTTATCATGAATCACCAAAAAGCCCCGAAGGGCATTGGTTTTTTATCTAATAAACACATCCCTTCCGAAGTCGGGATTTTCAGCATGTATTAGCTCTAGAATTACCACGGTTATCCAAGTAGTAAGGTATTATCAAATAAACGATAACTGATTTAATGAGCCATTCGCAGTTTCACGGTATAAT

>HSDFQ2-4

TTATACCGTGAAACTGCGAATGGCTCATTAAATCAGTTATCGTTTATTTGATAATACCTTACTACTTGGATAACCGTGGTAATTCTAGAGCTAATACATGCTGAAAATCCCGACTTCGGAAGGGATGTGTTTATTAGATAAAAAACCAATGCCCTTCGGGGCTTTTTGGTGATTCATGATAACTTTACGGATCGCATAGCCTTGCGCTGGCGACGGTTCATTCAAATTTCTGCCCTATCAACTTTCGATGGTAAGGTATTGGCTTACCATGGTTTCAACGGGTAACGGGGAATTAGGGTTCGATTCCGGAGAGGGAGCCTGAGAAACGGCTACCACATCCAAGGAAGGCAGCAGGCGCGCAAATTACCCAATCCCGACACGGGGAGGTAGTGACAATAAATACTGATACAGGGCTCTTTTGGGTCTTGTAATTGGAATGAGTACAATTTAAACCTCTTAACGAGGAACAATTGGAGGGCAAGTCTGGTGCCAGCAGCCGCGGTAATTCCAGCTCCAATAGCGTATATTAAAGTTGTTGCAGTTAAAAAGCTCGTAGTTGAAACTTGGGCCTGGCTGGCGGGTCCGCCTCACCGCGTGCACTCGTCCGGCCGGGCCTTCCTTCTGAAGAACCTCATGCCCTTCACTGGGCGTGCTGGGGAATCAGGACTTTTACTTTGAAAAAATTAGAGTGTTCAAAGCAGGCCTTTGCTCGAATACGTTAGCATGGAATAATAAAATAGGGCGTGCGTTTCTATTTTGTTGGTTTCTAGAGACGCCGCAATGATTAACAGGAACAGTCGGGGGCATCAGTATTCAGTTGTCAGAGGTGAAATTCTTGGATTTACTGAAGACTAACTACTGCGAAAGCATTTGCCAAGGATGTTTTCATTAATCAGTGAACGAAAGTTAGGGGATCGAAGACGATCAGATACCGTCGTAGTCTTAACCGTAAACTATGCCGACTAGGGATCGGGCGATGTTCTTTTTCTGACTCGCTCGGCACCTTACGAGAAATCAAAGTTTTT

>KP124917 Alternaria alstroemeriae

TATACCGTGAAACTGCGAATGGCTCATTAAATCAGTTATCGTTTATTTGATAATACCTTACTACTTGGATAACCGTGGTAATTCTAGAGCTAATACATGCTGAAAATCCCGACTTCGGAAGGGATGTGTTTATTAGATAAAAAACCAATGCCCTTCGGGGCTTTTTGGTGATTCATGATAACTTTACGGATCGCATAGCCTTGCGCTGGCGACGGTTCATTCAAATTTCTGCCCTATCAACTTTCGATGGTAAGGTATTGGCTTACCATGGTTTCAACGGGTAACGGGGAATTAGGGTTCGATTCCGGAGAGGGAGCCTGAGAAACGGCTACCACATCCAAGGAAGGCAGCAGGCGCGCAAATTACCCAATCCCGACACGGGGAGGTAGTGACAATAAATACTGATACAGGGCTCTTTTGGGTCTTGTAATTGGAATGAGTACAATTTAAACCTCTTAACGAGGAACAATTGGAGGGCAAGTCTGGTGCCAGCAGCCGCGGTAATTCCAGCTCCAATAGCGTATATTAAAGTTGTTGCAGTTAAAAAGCTCGTAGTTGAAACTTGGGCCTGGCTGGCGGGTCCGCCTCACCGCGTGCACTCGTCCGGCCGGGCCTTCCTTCTGAAGAACCTCATGCCCTTCACTGGGCGTGCTGGGGAATCAGGACTTTTACTTTGAAAAAATTAGAGTGTTCAAAGCAGGCCTTTGCTCGAATACGTTAGCATGGAATAATAAAATAGGGCGTGCGTTTCTATTTTGTTGGTTTCTAGAGACGCCGCAATGATTAACAGGAACAGTCGGGGGCATCAGTATTCAGTTGTCAGAGGTGAAATTCTTGGATTTACTGAAGACTAACTACTGCGAAAGCATTTGCCAAGGATGTTTTCATTAATCAGTGAACGAAAGTTAGGGGATCGAAGACGATCAGATACCGTCGTAGTCTTAACCGTAAACTATGCCGACTAGGGATCGGGCGATGTTCTTTTTCTGACTCGCTCGGCACCTTACGAGAAATCAAAGT

>KP124918 Alternaria alstroemeriae

TATACCGTGAAACTGCGAATGGCTCATTAAATCAGTTATCGTTTATTTGATAATACCTTACTACTTGGATAACCGTGGTAATTCTAGAGCTAATACATGCTGAAAATCCCGACTTCGGAAGGGATGTGTTTATTAGATAAAAAACCAATGCCCTTCGGGGCTTTTTGGTGATTCATGATAACTTTACGGATCGCATAGCCTTGCGCTGGCGACGGTTCATTCAAATTTCTGCCCTATCAACTTTCGATGGTAAGGTATTGGCTTACCATGGTTTCAACGGGTAACGGGGAATTAGGGTTCGATTCCGGAGAGGGAGCCTGAGAAACGGCTACCACATCCAAGGAAGGCAGCAGGCGCGCAAATTACCCAATCCCGACACGGGGAGGTAGTGACAATAAATACTGATACAGGGCTCTTTTGGGTCTTGTAATTGGAATGAGTACAATTTAAACCTCTTAACGAGGAACAATTGGAGGGCAAGTCTGGTGCCAGCAGCCGCGGTAATTCCAGCTCCAATAGCGTATATTAAAGTTGTTGCAGTTAAAAAGCTCGTAGTTGAAACTTGGGCCTGGCTGGCGGGTCCGCCTCACCGCGTGCACTCGTCCGGCCGGGCCTTCCTTCTGAAGAACCTCATGCCCTTCACTGGGCGTGCTGGGGAATCAGGACTTTTACTTTGAAAAAATTAGAGTGTTCAAAGCAGGCCTTTGCTCGAATACGTTAGCATGGAATAATAAAATAGGGCGTGCGTTTCTATTTTGTTGGTTTCTAGAGACGCCGCAATGATTAACAGGAACAGTCGGGGGCATCAGTATTCAGTTGTCAGAGGTGAAATTCTTGGATTTACTGAAGACTAACTACTGCGAAAGCATTTGCCAAGGATGTTTTCATTAATCAGTGAACGAAAGTTAGGGGATCGAAGACGATCAGATACCGTCGTAGTCTTAACCGTAAACTATGCCGACTAGGGATCGGGCGATGTTCTTTTTCTGACTCGCTCGGCACCTTACGAGAAATCAAAGT

>KC584506 Alternaria alternantherae

TATACCGTGAAACTGCGAATGGCTCATTAAATCAGTTATCGTTTATTTGATAATACCTTACTACTTGGATAACCGTGGTAATTCTAGAGCTAATACATGCTGAAAATCCCGACTTCGGAAGGGATGTGTTTATTAGATAAAAAACCAATGCCCTTCGGGGCTTTTTGGTGATTCATGATAACTTTACGGATCGCATAGCCTTGCGCTGGCGACGGTTCATTCAAATTTCTGCCCTATCAACTTTCGATGGTAAGGTATTGGCTTACCATGGTTTCAACGGGTAACGGGGAATTAGGGTTCGATTCCGGAGAGGGAGCCTGAGAAACGGCTACCACATCCAAGGAAGGCAGCAGGCGCGCAAATTACCCAATCCCGACACGGGGAGGTAGTGACAATAAATACTGATACAGGGCTCTTTTGGGTCTTGTAATTGGAATGAGTACAATTTAAACCTCTTAACGAGGAACAATTGGAGGGCAAGTCTGGTGCCAGCAGCCGCGGTAATTCCAGCTCCAATAGCGTATATTAAAGTTGTTGCAGTTAAAAAGCTCGTAGTTGAAACTTGGGCCTGGCTGGCGGGTCCGCCTCACCGCGTGCACTCGTCCGGCCGGGCCTTCCTTCTGAAGAACCTCATGCCCTTCACTGGGCGTGCTGGGGAATCAGGACTTTTACTTTGAAAAAATTAGAGTGTTCAAAGCAGGCCTTTGCTCGAATACGTTAGCATGGAATAATAAAATAGGGCGTGCGTTTCTATTTTGTTGGTTTCTAGAGACGCCGCAATGATTAACAGGAACAGTCGGGGGCATCAGTATTCAGTTGTCAGAGGTGAAATTCTTGGATTTACTGAAGACTAACTACTGCGAAAGCATTTGCCAAGGATGTTTTCATTAATCAGTGAACGAAAGTTAGGGGATCGAAGACGATCAGATACCGTCGTAGTCTTAACCGTAAACTATGCCGACTAGGGATCGGGCGATGTTCTTTTTCTGACTCGCTCGGCACCTTACGAGAAATCAAAGT

>KC584507 Alternaria alternata

TATACCGTGAAACTGCGAATGGCTCATTAAATCAGTTATCGTTTATTTGATAATACCTTACTACTTGGATAACCGTGGTAATTCTAGAGCTAATACATGCTGAAAATCCCGACTTCGGAAGGGATGTGTTTATTAGATAAAAAACCAATGCCCTTCGGGGCTTTTTGGTGATTCATGATAACTTTACGGATCGCATAGCCTTGCGCTGGCGACGGTTCATTCAAATTTCTGCCCTATCAACTTTCGATGGTAAGGTATTGGCTTACCATGGTTTCAACGGGTAACGGGGAATTAGGGTTCGATTCCGGAGAGGGAGCCTGAGAAACGGCTACCACATCCAAGGAAGGCAGCAGGCGCGCAAATTACCCAATCCCGACACGGGGAGGTAGTGACAATAAATACTGATACAGGGCTCTTTTGGGTCTTGTAATTGGAATGAGTACAATTTAAACCTCTTAACGAGGAACAATTGGAGGGCAAGTCTGGTGCCAGCAGCCGCGGTAATTCCAGCTCCAATAGCGTATATTAAAGTTGTTGCAGTTAAAAAGCTCGTAGTTGAAACTTGGGCCTGGCTGGCGGGTCCGCCTCACCGCGTGCACTCGTCCGGCCGGGCCTTCCTTCTGAAGAACCTCATGCCCTTCACTGGGCGTGCTGGGGAATCAGGACTTTTACTTTGAAAAAATTAGAGTGTTCAAAGCAGGCCTTTGCTCGAATACGTTAGCATGGAATAATAAAATAGGGCGTGCGTTTCTATTTTGTTGGTTTCTAGAGACGCCGCAATGATTAACAGGAACAGTCGGGGGCATCAGTATTCAGTTGTCAGAGGTGAAATTCTTGGATTTACTGAAGACTAACTACTGCGAAAGCATTTGCCAAGGATGTTTTCATTAATCAGTGAACGAAAGTTAGGGGATCGAAGACGATCAGATACCGTCGTAGTCTTAACCGTAAACTATGCCGACTAGGGATCGGGCGATGTTCTTTTTCTGACTCGCTCGGCACCTTACGAGAAATCAAAGT

>KC584578 Alternaria sp.

TATACCGTGAAACTGCGAATGGCTCATTAAATCAGTTATCGTTTATTTGATAATACCTTACTACTTGGATAACCGTGGTAATTCTAGAGCTAATACATGCTGAAAATCCCGACTTCGGAAGGGATGTGTTTATTAGATAAAAAACCAATGCCCTTCGGGGCTTTTTGGTGATTCATGATAACTTTACGGATCGCATAGCCTTGCGCTGGCGACGGTTCATTCAAATTTCTGCCCTATCAACTTTCGATGGTAAGGTATTGGCTTACCATGGTTTCAACGGGTAACGGGGAATTAGGGTTCGATTCCGGAGAGGGAGCCTGAGAAACGGCTACCACATCCAAGGAAGGCAGCAGGCGCGCAAATTACCCAATCCCGACACGGGGAGGTAGTGACAATAAATACTGATACAGGGCTCTTTTGGGTCTTGTAATTGGAATGAGTACAATTTAAACCTCTTAACGAGGAACAATTGGAGGGCAAGTCTGGTGCCAGCAGCCGCGGTAATTCCAGCTCCAATAGCGTATATTAAAGTTGTTGCAGTTAAAAAGCTCGTAGTTGAAACTTGGGCCTGGCTGGCGGGTCCGCCTCACCGCGTGCACTCGTCCGGCCGGGCCTTCCTTCTGAAGAACCTCATGCCCTTCACTGGGCGTGCTGGGGAATCAGGACTTTTACTTTGAAAAAATTAGAGTGTTCAAAGCAGGCCTTTGCTCGAATACGTTAGCATGGAATAATAAAATAGGGCGTGCGTTTCTATTTTGTTGGTTTCTAGAGACGCCGCAATGATTAACAGGAACAGTCGGGGGCATCAGTATTCAGTTGTCAGAGGTGAAATTCTTGGATTTACTGAAGACTAACTACTGCGAAAGCATTTGCCAAGGATGTTTTCATTAATCAGTGAACGAAAGTTAGGGGATCGAAGACGATCAGATACCGTCGTAGTCTTAACCGTAAACTATGCCGACTAGGGATCGGGCGATGTTCTTTTTCTGACTCGCTCGGCACCTTACGAGAAATCAAAGT

>KP124992 Alternaria alternata

TATACCGTGAAACTGCGAATGGCTCATTAAATCAGTTATCGTTTATTTGATAATACCTTACTACTTGGATAACCGTGGTAATTCTAGAGCTAATACATGCTGAAAATCCCGACTTCGGAAGGGATGTGTTTATTAGATAAAAAACCAATGCCCTTCGGGGCTTTTTGGTGATTCATGATAACTTTACGGATCGCATAGCCTTGCGCTGGCGACGGTTCATTCAAATTTCTGCCCTATCAACTTTCGATGGTAAGGTATTGGCTTACCATGGTTTCAACGGGTAACGGGGAATTAGGGTTCGATTCCGGAGAGGGAGCCTGAGAAACGGCTACCACATCCAAGGAAGGCAGCAGGCGCGCAAATTACCCAATCCCGACACGGGGAGGTAGTGACAATAAATACTGATACAGGGCTCTTTTGGGTCTTGTAATTGGAATGAGTACAATTTAAACCTCTTAACGAGGAACAATTGGAGGGCAAGTCTGGTGCCAGCAGCCGCGGTAATTCCAGCTCCAATAGCGTATATTAAAGTTGTTGCAGTTAAAAAGCTCGTAGTTGAAACTTGGGCCTGGCTGGCGGGTCCGCCTCACCGCGTGCACTCGTCCGGCCGGGCCTTCCTTCTGAAGAACCTCATGCCCTTCACTGGGCGTGCTGGGGAATCAGGACTTTTACTTTGAAAAAATTAGAGTGTTCAAAGCAGGCCTTTGCTCGAATACGTTAGCATGGAATAATAAAATAGGGCGTGCGTTTCTATTTTGTTGGTTTCTAGAGACGCCGCAATGATTAACAGGAACAGTCGGGGGCATCAGTATTCAGTTGTCAGAGGTGAAATTCTTGGATTTACTGAAGACTAACTACTGCGAAAGCATTTGCCAAGGATGTTTTCATTAATCAGTGAACGAAAGTTAGGGGATCGAAGACGATCAGATACCGTCGTAGTCTTAACCGTAAACTATGCCGACTAGGGATCGGGCGATGTTCTTTTTCTGACTCGCTCGGCACCTTACGAGAAATCAAAGT

>KP125062 Alternaria jacinthicola

TATACCGTGAAACTGCGAATGGCTCATTAAATCAGTTATCGTTTATTTGATAATACCTTACTACTTGGATAACCGTGGTAATTCTAGAGCTAATACATGCTGAAAATCCCGACTTCGGAAGGGATGTGTTTATTAGATAAAAAACCAATGCCCTTCGGGGCTTTTTGGTGATTCATGATAACTTTACGGATCGCATAGCCTTGCGCTGGCGACGGTTCATTCAAATTTCTGCCCTATCAACTTTCGATGGTAAGGTATTGGCTTACCATGGTTTCAACGGGTAACGGGGAATTAGGGTTCGATTCCGGAGAGGGAGCCTGAGAAACGGCTACCACATCCAAGGAAGGCAGCAGGCGCGCAAATTACCCAATCCCGACACGGGGAGGTAGTGACAATAAATACTGATACAGGGCTCTTTTGGGTCTTGTAATTGGAATGAGTACAATTTAAACCTCTTAACGAGGAACAATTGGAGGGCAAGTCTGGTGCCAGCAGCCGCGGTAATTCCAGCTCCAATAGCGTATATTAAAGTTGTTGCAGTTAAAAAGCTCGTAGTTGAAACTTGGGCCTGGCTGGCGGGTCCGCCTCACCGCGTGCACTCGTCCGGCCGGGCCTTCCTTCTGAAGAACCTCATGCCCTTCACTGGGCGTGCTGGGGAATCAGGACTTTTACTTTGAAAAAATTAGAGTGTTCAAAGCAGGCCTTTGCTCGAATACGTTAGCATGGAATAATAAAATAGGGCGTGCGTTTCTATTTTGTTGGTTTCTAGAGACGCCGCAATGATTAACAGGAACAGTCGGGGGCATCAGTATTCAGTTGTCAGAGGTGAAATTCTTGGATTTACTGAAGACTAACTACTGCGAAAGCATTTGCCAAGGATGTTTTCATTAATCAGTGAACGAAAGTTAGGGGATCGAAGACGATCAGATACCGTCGTAGTCTTAACCGTAAACTATGCCGACTAGGGATCGGGCGATGTTCTTTTTCTGACTCGCTCGGCACCTTACGAGAAATCAAAGT

>KP125064 Alternaria longipes

TATACCGTGAAACTGCGAATGGCTCATTAAATCAGTTATCGTTTATTTGATAATACCTTACTACTTGGATAACCGTGGTAATTCTAGAGCTAATACATGCTGAAAATCCCGACTTCGGAAGGGATGTGTTTATTAGATAAAAAACCAATGCCCTTCGGGGCTTTTTGGTGATTCATGATAACTTTACGGATCGCATAGCCTTGCGCTGGCGACGGTTCATTCAAATTTCTGCCCTATCAACTTTCGATGGTAAGGTATTGGCTTACCATGGTTTCAACGGGTAACGGGGAATTAGGGTTCGATTCCGGAGAGGGAGCCTGAGAAACGGCTACCACATCCAAGGAAGGCAGCAGGCGCGCAAATTACCCAATCCCGACACGGGGAGGTAGTGACAATAAATACTGATACAGGGCTCTTTTGGGTCTTGTAATTGGAATGAGTACAATTTAAACCTCTTAACGAGGAACAATTGGAGGGCAAGTCTGGTGCCAGCAGCCGCGGTAATTCCAGCTCCAATAGCGTATATTAAAGTTGTTGCAGTTAAAAAGCTCGTAGTTGAAACTTGGGCCTGGCTGGCGGGTCCGCCTCACCGCGTGCACTCGTCCGGCCGGGCCTTCCTTCTGAAGAACCTCATGCCCTTCACTGGGCGTGCTGGGGGAATCAGGACTTTTACTTTGAAAAAATTAGAGTGTTCAAAGCAGGCCTTTGCTCGAATACGTTAGCATGGAATAATAAAATAGGGCGTGCGTTTCTATTTTGTTGGTTTCTAGAGACGCCGCAATGATTAACAGGAACAGTCGGGGGCATCAGTATTCAGTTGTCAGAGGTGAAATTCTTGGATTTACTGAAGACTAACTACTGCGAAAGCATTTGCCAAGGATGTTTTCATTAATCAGTGAACGAAAGTTAGGGGATCGAAGACGATCAGATACCGTCGTAGTCTTAACCGTAAACTATGCCGACTAGGGATCGGGCGATGTTCTTTTTCTGACTCGCTCGGCACCTTACGAGAAATCAAAGT

>KP125068 Alternaria longipes

TATACCGTGAAACTGCGAATGGCTCATTAAATCAGTTATCGTTTATTTGATAATACCTTACTACTTGGATAACCGTGGTAATTCTAGAGCTAATACATGCTGAAAATCCCGACTTCGGAAGGGATGTGTTTATTAGATAAAAAACCAATGCCCTTCGGGGCTTTTTGGTGATTCATGATAACTTTACGGATCGCATAGCCTTGCGCTGGCGACGGTTCATTCAAATTTCTGCCCTATCAACTTTCGATGGTAAGGTATTGGCTTACCATGGTTTCAACGGGTAACGGGGAATTAGGGTTCGATTCCGGAGAGGGAGCCTGAGAAACGGCTACCACATCCAAGGAAGGCAGCAGGCGCGCAAATTACCCAATCCCGACACGGGGAGGTAGTGACAATAAATACTGATACAGGGCTCTTTTGGGTCTTGTAATTGGAATGAGTACAATTTAAACCTCTTAACGAGGAACAATTGGAGGGCAAGTCTGGTGCCAGCAGCCGCGGTAATTCCAGCTCCAATAGCGTATATTAAAGTTGTTGCAGTTAAAAAGCTCGTAGTTGAAACTTGGGCCTGGCTGGCGGGTCCGCCTCACCGCGTGCACTCGTCCGGCCGGGCCTTCCTTCTGAAGAACCTCATGCCCTTCACTGGGCGTGCTGGGGGAATCAGGACTTTTACTTTGAAAAAATTAGAGTGTTCAAAGCAGGCCTTTGCTCGAATACGTTAGCATGGAATAATAAAATAGGGCGTGCGTTTCTATTTTGTTGGTTTCTAGAGACGCCGCAATGATTAACAGGAACAGTCGGGGGCATCAGTATTCAGTTGTCAGAGGTGAAATTCTTGGATTTACTGAAGACTAACTACTGCGAAAGCATTTGCCAAGGATGTTTTCATTAATCAGTGAACGAAAGTTAGGGGATCGAAGACGATCAGATACCGTCGTAGTCTTAACCGTAAACTATGCCGACTAGGGATCGGGCGATGTTCTTTTTCTGACTCGCTCGGCACCTTACGAGAAATCAAAGT

>KP124987 Alternaria alternata

TATACCGTGAAACTGCGAATGGCTCATTAAATCAGTTATCGTTTATTTGATAATACCTTACTACTTGGATAACCGTGGTAATTCTAGAGCTAATACATGCTGAAAATCCCGACTTCGGAAGGGATGTGTTTATTAGATAAAAAACCAATGCCCTTCGGGGCTTTTTGGTGATTCATGATAACTTTACGGATCGCATAGCCTTGCGCTGGCGACGGTTCATTCAAATTTCTGCCCTATCAACTTTCGATGGTAAGGTATTGGCTTACCATGGTTTCAACGGGTAACGGGGAATTAGGGTTCGATTCCGGAGAGGGAGCCTGAGAAACGGCTACCACATCCAAGGAAGGCAGCAGGCGCGCAAATTACCCAATCCCGACACGGGGAGGTAGTGACAATAAATACTGATACAGGGCTCTTTTGGGTCTTGTAATTGGAATGAGTACAATTTAAACCTCTTAACGAGGAACAATTGGAGGGCAAGTCTGGTGCCAGCAGCCGCGGTAATTCCAGCTCCAATAGCGTATATTAAAGTTGTTGCAGTTAAAAAGCTCGTAGTTGAAACTTGGGCCTGGCTGGCGGGTCCGCCTCACCGCGTGCACTCGTCCGGCCGGGCCTTCCTTCTGAAGAACCTCATGCCCTTCACTGGGCGTGCTGGGGAATCAGGACTTTTACTTTGAAAAAATTAGAGTGTTCAAAGCAGGCCTTTGCTCGAATACGTTAGCATGGAATAATAAAATAGGGCGTGCGTTTCTATTTTGTTGGTTTCTAGAGACGCCGCAATGATTAACAGGAACAGTCGGGGGCATCAGTATTCAGTTGTCAGAGGTGAAATTCTTGGATTTACTGAAGACTAACTACTGCGAAAGCATTTGCCAAGGATGTTTTCATTAATCAGTGAACGAAAGTTAGGGGATCGAAGACGATCAGATACCGTCGTAGTCTTAACCGTAAACTATGCCGACTAGGGATCGGGCGATGTTCTTTTTCTGACTCGCTCGGCACCTTACGAGAAATCAAAGT

>KC584567 Alternaria tenuissima

TATACCGTGAAACTGCGAATGGCTCATTAAATCAGTTATCGTTTATTTGATAATACCTTACTACTTGGATAACCGTGGTAATTCTAGAGCTAATACATGCTGAAAATCCCGACTTCGGAAGGGATGTGTTTATTAGATAAAAAACCAATGCCCTTCGGGGCTTTTTGGTGATTCATGATAACTTTACGGATCGCATAGCCTTGCGCTGGCGACGGTTCATTCAAATTTCTGCCCTATCAACTTTCGATGGTAAGGTATTGGCTTACCATGGTTTCAACGGGTAACGGGGAATTAGGGTTCGATTCCGGAGAGGGAGCCTGAGAAACGGCTACCACATCCAAGGAAGGCAGCAGGCGCGCAAATTACCCAATCCCGACACGGGGAGGTAGTGACAATAAATACTGATACAGGGCTCTTTTGGGTCTTGTAATTGGAATGAGTACAATTTAAACCTCTTAACGAGGAACAATTGGAGGGCAAGTCTGGTGCCAGCAGCCGCGGTAATTCCAGCTCCAATAGCGTATATTAAAGTTGTTGCAGTTAAAAAGCTCGTAGTTGAAACTTGGGCCTGGCTGGCGGGTCCGCCTCACCGCGTGCACTCGTCCGGCCGGGCCTTCCTTCTGAAGAACCTCATGCCCTTCACTGGGCGTGCTGGGGAATCAGGACTTTTACTTTGAAAAAATTAGAGTGTTCAAAGCAGGCCTTTGCTCGAATACGTTAGCATGGAATAATAAAATAGGGCGTGCGTTTCTATTTTGTTGGTTTCTAGAGACGCCGCAATGATTAACAGGAACAGTCGGGGGCATCAGTATTCAGTTGTCAGAGGTGAAATTCTTGGATTTACTGAAGACTAACTACTGCGAAAGCATTTGCCAAGGATGTTTTCATTAATCAGTGAACGAAAGTTAGGGGATCGAAGACGATCAGATACCGTCGTAGTCTTAACCGTAAACTATGCCGACTAGGGATCGGGCGATGTTCTTTTTCTGACTCGCTCGGCACCTTACGAGAAATCAAAGT

>KP124937 Alternaria alternata

TATACCGTGAAACTGCGAATGGCTCATTAAATCAGTTATCGTTTATTTGATAATACCTTACTACTTGGATAACCGTGGTAATTCTAGAGCTAATACATGCTGAAAATCCCGACTTCGGAAGGGATGTGTTTATTAGATAAAAAACCAATGCCCTTCGGGGCTTTTTGGTGATTCATGATAACTTTACGGATCGCATAGCCTTGCGCTGGCGACGGTTCATTCAAATTTCTGCCCTATCAACTTTCGATGGTAAGGTATTGGCTTACCATGGTTTCAACGGGTAACGGGGAATTAGGGTTCGATTCCGGAGAGGGAGCCTGAGAAACGGCTACCACATCCAAGGAAGGCAGCAGGCGCGCAAATTACCCAATCCCGACACGGGGAGGTAGTGACAATAAATACTGATACAGGGCTCTTTTGGGTCTTGTAATTGGAATGAGTACAATTTAAACCTCTTAACGAGGAACAATTGGAGGGCAAGTCTGGTGCCAGCAGCCGCGGTAATTCCAGCTCCAATAGCGTATATTAAAGTTGTTGCAGTTAAAAAGCTCGTAGTTGAAACTTGGGCCTGGCTGGCGGGTCCGCCTCACCGCGTGCACTCGTCCGGCCGGGCCTTCCTTCTGAAGAACCTCATGCCCTTCACTGGGCGTGCTGGGGAATCAGGACTTTTACTTTGAAAAAATTAGAGTGTTCAAAGCAGGCCTTTGCTCGAATACGTTAGCATGGAATAATAAAATAGGGCGTGCGTTTCTATTTTGTTGGTTTCTAGAGACGCCGCAATGATTAACAGGAACAGTCGGGGGCATCAGTATTCAGTTGTCAGAGGTGAAATTCTTGGATTTACTGAAGACTAACTACTGCGAAAGCATTTGCCAAGGATGTTTTCATTAATCAGTGAACGAAAGTTAGGGGATCGAAGACGATCAGATACCGTCGTAGTCTTAACCGTAAACTATGCCGACTAGGGATCGGGCGATGTTCTTTTTCTGACTCGCTCGGCACCTTACGAGAAATCAAAGT

>KP124943 Alternaria alternata

TATACCGTGAAACTGCGAATGGCTCATTAAATCAGTTATCGTTTATTTGATAATACCTTACTACTTGGATAACCGTGGTAATTCTAGAGCTAATACATGCTGAAAATCCCGACTTCGGAAGGGATGTGTTTATTAGATAAAAAACCAATGCCCTTCGGGGCTTTTTGGTGATTCATGATAACTTTACGGATCGCATAGCCTTGCGCTGGCGACGGTTCATTCAAATTTCTGCCCTATCAACTTTCGATGGTAAGGTATTGGCTTACCATGGTTTCAACGGGTAACGGGGAATTAGGGTTCGATTCCGGAGAGGGAGCCTGAGAAACGGCTACCACATCCAAGGAAGGCAGCAGGCGCGCAAATTACCCAATCCCGACACGGGGAGGTAGTGACAATAAATACTGATACAGGGCTCTTTTGGGTCTTGTAATTGGAATGAGTACAATTTAAACCTCTTAACGAGGAACAATTGGAGGGCAAGTCTGGTGCCAGCAGCCGCGGTAATTCCAGCTCCAATAGCGTATATTAAAGTTGTTGCAGTTAAAAAGCTCGTAGTTGAAACTTGGGCCTGGCTGGCGGGTCCGCCTCACCGCGTGCACTCGTCCGGCCGGGCCTTCCTTCTGAAGAACCTCATGCCCTTCACTGGGCGTGCTGGGGAATCAGGACTTTTACTTTGAAAAAATTAGAGTGTTCAAAGCAGGCCTTTGCTCGAATACGTTAGCATGGAATAATAAAATAGGGCGTGCGTTTCTATTTTGTTGGTTTCTAGAGACGCCGCAATGATTAACAGGAACAGTCGGGGGCATCAGTATTCAGTTGTCAGAGGTGAAATTCTTGGATTTACTGAAGACTAACTACTGCGAAAGCATTTGCCAAGGATGTTTTCATTAATCAGTGAACGAAAGTTAGGGGATCGAAGACGATCAGATACCGTCGTAGTCTTAACCGTAAACTATGCCGACTAGGGATCGGGCGATGTTCTTTTTCTGACTCGCTCGGCACCTTACGAGAAATCAAAGT

>KP124962 Alternaria alternata

TATACCGTGAAACTGCGAATGGCTCATTAAATCAGTTATCGTTTATTTGATAATACCTTACTACTTGGATAACCGTGGTAATTCTAGAGCTAATACATGCTGAAAATCCCGACTTCGGAAGGGATGTGTTTATTAGATAAAAAACCAATGCCCTTCGGGGCTTTTTGGTGATTCATGATAACTTTACGGATCGCATAGCCTTGCGCTGGCGACGGTTCATTCAAATTTCTGCCCTATCAACTTTCGATGGTAAGGTATTGGCTTACCATGGTTTCAACGGGTAACGGGGAATTAGGGTTCGATTCCGGAGAGGGAGCCTGAGAAACGGCTACCACATCCAAGGAAGGCAGCAGGCGCGCAAATTACCCAATCCCGACACGGGGAGGTAGTGACAATAAATACTGATACAGGGCTCTTTTGGGTCTTGTAATTGGAATGAGTACAATTTAAACCTCTTAACGAGGAACAATTGGAGGGCAAGTCTGGTGCCAGCAGCCGCGGTAATTCCAGCTCCAATAGCGTATATTAAAGTTGTTGCAGTTAAAAAGCTCGTAGTTGAAACTTGGGCCTGGCTGGCGGGTCCGCCTCACCGCGTGCACTCGTCCGGCCGGGCCTTCCTTCTGAAGAACCTCATGCCCTTCACTGGGCGTGCTGGGGAATCAGGACTTTTACTTTGAAAAAATTAGAGTGTTCAAAGCAGGCCTTTGCTCGAATACGTTAGCATGGAATAATAAAATAGGGCGTGCGTTTCTATTTTGTTGGTTTCTAGAGACGCCGCAATGATTAACAGGAACAGTCGGGGGCATCAGTATTCAGTTGTCAGAGGTGAAATTCTTGGATTTACTGAAGACTAACTACTGCGAAAGCATTTGCCAAGGATGTTTTCATTAATCAGTGAACGAAAGTTAGGGGATCGAAGACGATCAGATACCGTCGTAGTCTTAACCGTAAACTATGCCGACTAGGGATCGGGCGATGTTCTTTTTCTGACTCGCTCGGCACCTTACGAGAAATCAAAGT

>KP125069 Alternaria tomato

TATACCGTGAAACTGCGAATGGCTCATTAAATCAGTTATCGTTTATTTGATAATACCTTACTACTTGGATAACCGTGGTAATTCTAGAGCTAATACATGCTGAAAATCCCGACTTCGGAAGGGATGTGTTTATTAGATAAAAAACCAATGCCCTTCGGGGCTTTTTGGTGATTCATGATAACTTTACGGATCGCATAGCCTTGCGCTGGCGACGGTTCATTCAAATTTCTGCCCTATCAACTTTCGATGGTAAGGTATTGGCTTACCATGGTTTCAACGGGTAACGGGGAATTAGGGTTCGATTCCGGAGAGGGAGCCTGAGAAACGGCTACCACATCCAAGGAAGGCAGCAGGCGCGCAAATTACCCAATCCCGACACGGGGAGGTAGTGACAATAAATACTGATACAGGGCTCTTTTGGGTCTTGTAATTGGAATGAGTACAATTTAAACCTCTTAACGAGGAACAATTGGAGGGCAAGTCTGGTGCCAGCAGCCGCGGTAATTCCAGCTCCAATAGCGTATATTAAAGTTGTTGCAGTTAAAAAGCTCGTAGTTGAAACTTGGGCCTGGCTGGCGGGTCCGCCTCACCGCGTGCACTCGTCCGGCCGGGCCTTCCTTCTGAAGAACCTCATGCCCTTCACTGGGCGTGCTGGGGAATCAGGACTTTTACTTTGAAAAAATTAGAGTGTTCAAAGCAGGCCTTTGCTCGAATACGTTAGCATGGAATAATAAAATAGGGCGTGCGTTTCTATTTTGTTGGTTTCTAGAGACGCCGCAATGATTAACAGGAACAGTCGGGGGCATCAGTATTCAGTTGTCAGAGGTGAAATTCTTGGATTTACTGAAGACTAACTACTGCGAAAGCATTTGCCAAGGATGTTTTCATTAATCAGTGAACGAAAGTTAGGGGATCGAAGACGATCAGATACCGTCGTAGTCTTAACCGTAAACTATGCCGACTAGGGATCGGGCGATGTTCTTTTTCTGACTCGCTCGGCACCTTACGAGAAATCAAAGT

>KP124953 Alternaria alternata

TATACCGTGAAACTGCGAATGGCTCATTAAATCAGTTATCGTTTATTTGATAATACCTTACTACTTGGATAACCGTGGTAATTCTAGAGCTAATACATGCTGAAAATCCCGACTTCGGAAGGGATGTGTTTATTAGATAAAAAACCAATGCCCTTCGGGGCTTTTTGGTGATTCATGATAACTTTACGGATCGCATAGCCTTGCGCTGGCGACGGTTCATTCAAATTTCTGCCCTATCAACTTTCGATGGTAAGGTATTGGCTTACCATGGTTTCAACGGGTAACGGGGAATTAGGGTTCGATTCCGGAGAGGGAGCCTGAGAAACGGCTACCACATCCAAGGAAGGCAGCAGGCGCGCAAATTACCCAATCCCGACACGGGGAGGTAGTGACAATAAATACTGATACAGGGCTCTTTTGGGTCTTGTAATTGGAATGAGTACAATTTAAACCTCTTAACGAGGAACAATTGGAGGGCAAGTCTGGTGCCAGCAGCCGCGGTAATTCCAGCTCCAATAGCGTATATTAAAGTTGTTGCAGTTAAAAAGCTCGTAGTTGAAACTTGGGCCTGGCTGGCGGGTCCGCCTCACCGCGTGCACTCGTCCGGCCGGGCCTTCCTTCTGAAGAACCTCATGCCCTTCACTGGGCGTGCTGGGGAATCAGGACTTTTACTTTGAAAAAATTAGAGTGTTCAAAGCAGGCCTTTGCTCGAATACGTTAGCATGGAATAATAAAATAGGGCGTGCGTTTCTATTTTGTTGGTTTCTAGAGACGCCGCAATGATTAACAGGAACAGTCGGGGGCATCAGTATTCAGTTGTCAGAGGTGAAATTCTTGGATTTACTGAAGACTAACTACTGCGAAAGCATTTGCCAAGGATGTTTTCATTAATCAGTGAACGAAAGTTAGGGGATCGAAGACGATCAGATACCGTCGTAGTCTTAACCGTAAACTATGCCGACTAGGGATCGGGCGATGTTCTTTTTCTGACTCGCTCGGCACCTTACGAGAAATCAAAGT

Gnomoniopsis（ITS）

>XP1-6

GGTTGTTCCTTCCCGCTTTAGATATGCTTAAGTTCAGCCGGCATTCCTACCTGATCCGAGGTCAATTTTCAGAAAAGTTGGGGGGTTTTACGGCAAGAACATCACTAGTCTTTACAAACGAGGTATAAAAATTACTACGCTCAAAATTCTAGCGAGCCCGCCACTAAATTTCAGAGGATACTCTTTTACAAGTATTCCTCCAACACCAAAACAGAAGTTTTGAGGGTTGAAATGACGCTCGAACAGGCATGCCCGCTGGAATACCAGCGGGCGCAATGTGCGTTCAAAGATTCGATGATTCACTGAATTCTGCAATTCACATTACTTATCGCATTTCGCTGCGTTCTTCATCGATGCCAGAACCAAGAGATCCGTTGTTAAAAGTTTTGATTCATTTATAGTTTTTTTTACTCAGAAGATACATGGTATAAAAACAAGAGTTTCAGGGCCACCGGCCGACCTGCTCCTCTCTGTCTTCAACGCGTAAAACACATTGAATACTCGAGGGACCTTGCTAAAGAAAGCCAGTCAATGCCGAGGCAACAATAAGTATAATTCACAAAGGGTTTCTGGGTAGCACCCGTGAGGGCGTTTGTTCCAGCAATGATCCCTCCGAGGCCCCCCCAAAAGGGAGGGGAT

>TMR3-3

GGAGTTCCTCCCCCCTTTAGATATGCTTAAGTTCAGCGGGTATTCCTACCTGATCCGAGGTCAATTTTCAGAAAAGTTGGGGGGTTTTACGGCAAGAACATCACTAGTCTTTACAAACGAGGTATAAAAATTACTACGCTCAAAATTCTAGCGAGCCCGCCACTAAATTTCAGAGGATACTCTTTTACAAGTATTCCTCCAACACCAAAACAGAAGTTTTGAGGGTTGAAATGACGCTCGAACAGGCATGCCCGCTGGAATACCAGCGGGCGCAATGTGCGTTCAAAGATTCGATGATTCACTGAATTCTGCAATTCACATTACTTATCGCATTTCGCTGCGTTCTTCATCGATGCCAGAACCAAGAGATCCGTTGTTAAAAGTTTTGATTCATTTATAGTTTTTTTTACTCAGAAGATACATGGTATAAAAACAAGAGTTTCAGGGCCACCGGCCGACCTGCTCCTCTCTGTCTTCAACGCGTAAAACACATTGAATACTCGAGGGACCTTGCTAAAGAAAGCCAGTCAATGCCGAGGCAACAATAAGTATAATTCACAAAGGGTTTCTGGGTAGCACCCGTGAGGGCGTTGGAACCACCAATGATCCCTCCGAGGTCCCCCCCCCCCGGGGGGGGAT

>OP581244 Gnomoniopsis rosae GUCC 408.7

TAGTAACGGCGAGTGAAGCGGCAACAGCTCAAATTTGAAATCTGGCTTCGGCCCGAGTTGTAATTTGCAGAGGATGTTTATGGTGCGGTACCTTCCGAGTTCCCTGGAACGGGACGCCACAGAGGGTGAGAGCCCCGTCTGGTTGGATACCAAACCTGTGTTAAACTCCTTCAACGAGTCGAGTAGTTTGGGAATGCTGCTCTAAATGGGAGGTAAATCTCTTCTAAAGCTAAATACCGGCCAGAGACCGATAGCGCACAAGTAGAGTGATCGAAAGATGAAAAGCACCTTGAAAAGGGGGTTAAACAGTACGTGAAATTGTTAAAAGGGAAGCGTTTATGACCAGACTTGTGTCGTGTGGCTCATCCGAGGTTCTCCCCGGTGCACTCCACACGGCTCAGGCCAACATCGGTTCTCGTTGGGGGATAAGAACAGTAGGAACGTGGCCCTCTTCGGAGGGTGTTATAGCCTGCTGTACGATACCCTGATGGGGACCGAGGACCGCGCTTCGGCTAGGATGTTGGCGTAATGGTCATTAGCGACCCGTCTTGAAACACGGACCAAGGAGTCGTCCATTAGAGCGAGCGTTTGGGTGTAAAACCCGCACGCGTAATGAAAGTGAAATTAGGTGAGAGCTTCGGCGCATCATCGACCGATCCTGAAGTTTACGGATGGATTTGAGTAAGAGTTTTAACGGACGGACCCGAAAGACAGTGAACTATGCTTGAATAGGGTGAAGCCAGAGGAAACTCTGGTGGAGGCTCGCAGCGGTTCTGACGTGCAAATCGATCGTCAAATTTGAGCATGGGGGCGAAAGACTAATCGAACTGTCTAGTAGCT

>OP581231 rosae GUCC 408.7

GCGTAACAAGGTCTCCGTTGGTGAACCAGCGGAGGGATCATTGCTGGAACAAACGCCCTCACGGGTGCTACCCAGAAACCCTTTGTGAATTATACTTATTGTTGCCTCGGCATTGACTGGCTTTCTTTAGCAAGGTCCCTCGAGTATTCAATGTGTTTTACGCGTTGAAGACAGAGAGGAGCAGGTCGGCCGGTGGCCCTGAAACTCTTGTTTTTATACCATGTATCTTCTGAGTAAAACAACTATAAATGAATCAAAACTTTTAACAACGGATCTCTTGGTTCTGGCATCGATGAAGAACGCAGCGAAATGCGATAAGTAATGTGAATTGCAGAATTCAGTGAATCATCGAATCTTTGAACGCACATTGCGCCCGCTGGTATTCCAGCGGGCATGCCTGTTCGAGCGTCATTTCAACCCTCAAAACTTCTGTTTTGGTGTTGGAGGAATACTTGTAAAAGAGTATCCTCTGAAATTTAGTGGCGGGCTCGCTAGAATTTTGAGCGTAGTAATTTTTATACCTCGTTTGTAAAGACTAGTGATGTTCTTGCCGTAAAACCCCCCAACTTTTCTGAAAATTGACCTCGGATCAGGTAGGAATACCCGCTGAACTTAAGCATATC

>DQ313525.1 Apiognomonia errabunda strain AR 2813

TCATTGCTGGAACAAACGCCCTCACGGGTGCTACCCAGAAACCCTTTGTGAATACTACCTAAAATGTTGCCTCGGCATTGGTTGGCCTCTTTGAGGTCCCTTTCCCTCGGGGAAGGAGCAGACCGGCCGGTGGCCCTATAAACTCTTGTTTTTGTAATATCATCTGAGTAAAAAACATAAATAAATCAAAACTTTCAACAACGGATCTCTTGGTTCTGGCATCGATGAAGAACGCAGCGAAATGCGATAAGTAATGTGAATTGCAGAATTCAGTGAATCATCGAATCTTTGAACGCACATTGCGCCCGGTGGTATTCCACCGGGCATGCCTGTTCGAGCGTCATTTCAACCCTCAAAGCTTGCTTTGGTGTTGGAGGAATACCCAGTAAAACGGGTACCCTCTGAAATTTAGTGGCGGGCTCGCTAGAATTTTGAGCGTAGTAATTTTACCTCGTTTTTAAAGACTAGTGGGACTTCTTGCCGTAAAACCCCCAACTTTCTGAAAATTGACCT

>GU320825.1 Gnomoniopsis alderdunense strain CBS 125680

TCATTGCTGGAACAAACGCCCCCACGGGTGCTACCCAGAAACCCTTTGTGAATTCTTCTCTATTGTTGCCTCGGCGCAGACTGGCTTCGTACGAAGTCCCTTGTTGGCAACAGCAAGGAGCAGGTCGGCCGGTGGCCCTATAAACTCTATGTTTTTCATGTATCTTCTGAGTAAACAATTATAAATGAATCAAAACTTTTAACAACGGATCTCTTGGTTCTGGCATCGATGAAGAACGCAGCGAAATGCGATAAGTAATGTGAATTGCAGAATTCAGTGAATCATCGAATCTTTGAACGCACATTGCGCCCGCTGGTATTCCAGCGGGCATGCCTGTTCGAGCGTCATTTCAACCCTCAAAACCTCGGTTTTGGTGTTGGAGGACTACGCAGTAAAATGCGTAGCCTCTGAAATTCAGTGGCGGGCTCGCTAGAATTTTGAGCGTAGTAATTTATACCTCGTTTGTAAAGACTAGTGATGCTTTTCTTGCCGTAAAACCCCC

>MN598671.1 Gnomoniopsis daii isolate CMF002A

TCATTGCTGGAATACACGCTCGCAAGAGTGCTACCCAGAAACCCTTTGTGAATTATTCTCAAAACGTTGCCTCGGCAGTGACTGGCTTTCTTAGGAAGTCCCTCTTCGGAGGAGCAGGTCGGCCGGTGGCCCTATAAACTCTTTGTTTTTTACAATGTATCTTCTGAGTAAAACAAATATAAATGAATCAAAACTTTTAACAACGGATCTCTTGGTTCTGGCATCGATGAAGAACGCAGCGAAATGCGATAAGTAATGTGAATTGCAGAATTCAGTGAATCATCGAATCTTTGAACGCACATTGCGCCCGCTGGTATTCCAGCGGGCATGCCTGTTCGAGCGTCATTTCAACCCTCAAAACTTCTGTTTTGGTGTTGGAGGACTACCTGTAAAAGGGTAGCCTCTGAAATTTAGTGGCGGGCTCGCTAGAATTTTGAGCGTAGTAATTTATACCTCGTTTAGGAAGACTAGTGATGTTCTTTGCCGTAAAACCCCCCAACTTTCTGA

>MZ902913.1 Gnomoniopsis sp. CFCC 54288

ATTGCTGGAACAAACGCCCTCACGGGTGCTACCCAGAAACCCTTTGTGAACTTTCTATAACACTGTTGCCTCGGCATGGACTGGCCCTTCTATGAGGGTCCCTCTTTTCGGAGAGGAGCAGGTCGGCCGGTGGCCCTATAAACTCTTTGTTTTTACACTGTATCTTCTGAGTAAACAACTATAAATGAATCAAAACTTTTAACAACGGATCTCTTGGTTCTGGCATCGATGAAGAACGCAGCGAAATGCGATAAGTAATGTGAATTGCAGAATTCAGTGAATCATCGAATCTTTGAACGCACATTGCGCCCGCTGGTATTCCAGCGGGCATGCCTGTTCGAGCGTCATTTCAACCCTCAAAACCTCGGTTTTGGTGTTGGAGGAACACCTGTCAAAGGGTGCCCTCTGAAATTTAGTGGCGGGCTCGCTAGAATTTTGAGCGTAGTAATTTTATACCTCGTTTGTAAAGACTAGCGGTTGCTTCTTGCCGTAAAACCCCC

>MZ902918 Gnomoniopsis sp.

TTGCTGGACAAACGCCCTCACGGGTGCTACCCAGAAACCCTTTGTGAATTATTCTCAAACGTTGCCTCGGCAGTTGACTGGCTTTGTAAAAAGTCCCTTTCTTGTAAAAGAAAGGAGCAGGTCGGCCGGTGGCCCTATAAACTCTTTGTTTTTACCATGTATCTTCTGAGTAAACAAAAAAAAAATGAATCAAAACTTTTAACAACGGATCTCTTGGTTCTGGCATCGATGAAAAACGCAGCGAAATGCGATAAGTAATGTGAATTGCAAAATTCAGTGAATCATCGAATCTTTGAACGCACATTGCGCCCGCTGGTATTCCAGCGGGCATGCCTGTTCGAGCGTCATTTCAACCCTCAAAACATCTTTTTGTTTTGGTGTTGGAGGACTACCTGTAAAAGGGTAGCCTCTGAAATTTAGTGGCGGGCTCGCTAAAATTTTGAGCGTAGTAATTTTTATCTTCGTTTGTAAARACTAGCGGTGTTTCTTTGCCGTAAAACCCCC

>MZ902921 Gnomoniopsis sp.

ATTGCTGGAACAAACGCCCTCACGGGTGCTACCCAGAAACCCTTTGTGAACTTTCTATAACTGTTGCCTCGGCATTGACTGGCTTCTTTATGAGGTCCCTTTCTTCGGAAAGGAGCAGGTCGGCCGGTGGCCCTATAAACTCTTGTTTTTACACTGTATCTTCTGAGTAAAAAATTATAAATGAATCAAAACTTTTAACAACGGATCTCTTGGTTCTGGCATCGATGAAGAACGCAGCGAAATGCGATAAGTAATGTGAATTGCAGAATTCAGTGAATCATCGAATCTTTGAACGCACATTGCGCCCGCTGGTATTCCAGCGGGCATGCCTGTTCGAGCGTCATTTCAACCCTCAAAACTCCGGTTTTGGTGTTGGAGGAATACCTGTAAAAGGGTACCCTCTGAAATTTAGTGGCGGGCTCGCTAGAATTTTGAGCGTAGTAATTTATACCTCGTTTGTAAAGTCTAGCGGTTCTCTTGCCGTAAAACCCCC

>MZ902922 Gnomoniopsis sp.

ATTGCTGGAACAAACGCCCTCACGGGTGCTACCCAGAAACCCTTTGTGAACTTTCTATAACTGTTGCCTCGGCATTGACTGGCTTCTTTATGAGGTCCCTTTCTTCGGAAAGGAGCAGGTCGGCCGGTGGCCCTATAAACTCTTGTTTTTACACTGTATCTTCTGAGTAAAAAATTATAAATGAATCAAAACTTTTAACAACGGATCTCTTGGTTCTGGCATCGATGAAGAACGCAGCGAAATGCGATAAGTAATGTGAATTGCAGAATTCAGTGAATCATCGAATCTTTGAACGCACATTGCGCCCGCTGGTATTCCAGCGGGCATGCCTGTTCGAGCGTCATTTCAACCCTCAAAACTCCGGTTTTGGTGTTGGAGGAATACCTGTAAAAGGGTACCCTCTGAAATTTAGTGGCGGGCTCGCTAGAATTTTGAGCGTAGTAATTTATACCTCGTTTGTAAAGTCTAGCGGTTCTCTTGCCGTAAAACCCCC

>EU254841 Gnomoniopsis racemula

TCATTGCTGGAACAAACGCCCTCACGGGTGCTACCCAGAAACCCTTTGTGAATTCTTCTCTATTGTTGCCTCGGCGCAGACTGGCTTCTTCACGAAGCCCCTTTGGCAACAAAGGAGCAGGTCGGCCGGTGGCCCTATAAACTCTATGTTTTTCTTGTATTTTCTGAGTAAACAACTATAAATGAATCAAAACTTTTAACAACGGATCTCTTGGTTCTGGCATCGATGAAGAACGCAGCGAAATGCGATAAGTAATGTGAATTGCAGAATTCAGTGAATCATCGAATCTTTGAACGCACATTGCGCCCGCTGGTATTCCAGCGGGCATGCCTGTTCGAGCGTCATTTCAACCCTCAAAACTTCGGTTTTGGTGTTGGAGGACTACGCAGTAACATGCGTAGCCTCTGAAATTCAGTGGCGGGCTCGCTAGAATTTTGAGCGTAGTAATTTATACCTCGTTTGTAAAGACTAGTGATGTTCTTGCCGTAAAACCCCCAACTTCCTGA

>MZ902923 Gnomoniopsis sp.

TTGCTGGACAACGCCTTCACGGGCGCTACCCAGAAACCCTTTGTGAATTTTTCCACTTAGTTGCCTCGGCAGTGACTGGCTTTTTTATAGAAGGTCCCTTCTCTCGCAGAAGGAGCAGGTCGGCCGGTGGCCCTATAAACTCTTTGTTTTTACAGTGTATCTTCTGAGTAACAATAACCAAAATGAATCAAAACTTTTAACAACGGATCTCTTGGTTCTGGCATCGATGAAGAACGCAGCGAAATGCGATAAGTAATGTGAATTGCAGAATTCAGTGAATCATCGAATCTTTGAACGCACATTGCGCCCGCTGGTATTCCAGCGGGCATGCCTGTTCGAGCGTCATTTCAACCCTCAAAACTTCTGTTTTGGTGTTGGAGGAATACCTGTAACAGGGTACCCTCTGAAATTTAGTGGCGGGCTCGCTAGAATTTTGAGCGTAGTAATTTTATACCTCGTTTGTAAAGACTAGTGAATCTCTTGCCGTAAAACCCCC

>MZ902924 Gnomoniopsis sp.

TTGCTGGACAACGCCTTCACGGGCGCTACCCAGAAACCCTTTGTGAATTTTTCCACTTAGTTGCCTCGGCAGTGACTGGCTTTTTTATAGAAGGTCCCTTCTCTCGCAGAAGGAGCAGGTCGGCCGGTGGCCCTATAAACTCTTTGTTTTTACAGTGTATCTTCTGAGTAACAATAACCAAAATGAATCAAAACTTTTAACAACGGATCTCTTGGTTCTGGCATCGATGAAGAACGCAGCGAAATGCGATAAGTAATGTGAATTGCAGAATTCAGTGAATCATCGAATCTTTGAACGCACATTGCGCCCGCTGGTATTCCAGCGGGCATGCCTGTTCGAGCGTCATTTCAACCCTCAAAACTTCTGTTTTGGTGTTGGAGGAATACCTGTAACAGGGTACCCTCTGAAATTTAGTGGCGGGCTCGCTAGAATTTTGAGCGTAGTAATTTTATACCTCGTTTGTAAAGACTAGTGAATCTCTTGCCGTAAAACCCCC

>GU320818 Gnomoniopsis sanguisorbae

TCATTGCTGGAATAAACGCCCTCACGGGTGCTACCCAGAAACCCTTTGTGAATTCTTCTTTATTGTTGCCTCGGCATAGACTGGCTTCCTACGAAGCCCCTTTTTGCAAAAAGAGGAGCAGGTCGGCCGGTGGCCCTATAAATTCTCTGTTTTTTTAATTTGTATCTTCTGAGTAAACAACTAAAAATGAATCAAAACTTTTAACAACGGATCTCTTGGTTCTGGCATCGATGAAGAACGCAGCGAAATGCGATAAGTAATGTGAATTGCAGAATTCAGTGAATCATCGAATCTTTGAACGCACATTGCGCCCGCTGGTATTCCAGCGGGCATGCCTGTTCGAGCGTCATTTCAACCCTCAAAACCTCGGTTTTGGTGTTGGAGGACTACGCGGTAAAACGCGTAGCCTCTGAAATGTAGTGGCGGGCTCGCTAGAATTTTGAGCGTAGTAATTTATACCTCGTTTGTGAAGACTAGTGATGATCCTTGCCGTAAAACCCCC

>MZ902925 Gnomoniopsis sp.

ATTGCTGGATACACGCTCGCAAGAGCGCTACCCAGAAACCCTTTGTGAATTTTTCTCAAATGTTGCCTCGGCAGTGACTGGCTCTCTATAGAGTCCCTCTTTTCTTTTGGAGAAGAGGAGCAGGTCGGCCGGTGGCCCTATAAACTCTTTGTTTTTACACTGTATTTTCTGAGTTTTTAAAAAATAAATGAATCAAAACTTTTAACAACGGATCTCTTGGTTCTGGCATCGATGAAGAACGCAGCGAAATGCGATAAGTAATGTGAATTGCAGAATTCAGTGAATCATCGAATCTTTGAACGCACATTGCGCCCGCTGGTATTCCAGCGGGCATGCCTGTTCGAGCGTCATTTCAACCCTCAAAACTTCTTGTTTTGGTGTTGGAGGACTACCTGTAAAAGGGTAGCCTCTGAAATTTAGTGGCGGGCTCGCTAGAATTTTGAGCGTAGTAATTTATACCTCGTTTATGAAGACTAGTGATGTTTCCTTTGCCGTAAAACCCCC

>MZ902926 Gnomoniopsis sp.

ATTGCTGGAATACACGCTCGCAAGAGCGCTACCCAGAAACCCTTTGTGAATTTTTCTCAAATGTTGCCTCGGCAGTGACTGGCTCTCTATAGAGTCCCTCTTTTCTTTTGGAGAAGAGGAGCAGGTCGGCCGGTGGCCCTATAAACTCTTTGTTTTTACACTGTATTTTCTGAGTTTTTAAAAAATAAATGAATCAAAACTTTTAACAACGGATCTCTTGGTTCTGGCATCGATGAAGAACGCAGCGAAATGCGATAAGTAATGTGAATTGCAGAATTCAGTGAATCATCGAATCTTTGAACGCACATTGCGCCCGCTGGTATTCCAGCGGGCATGCCTGTTCGAGCGTCATTTCAACCCTCAAAACTTCTTGTTTTGGTGTTGGAGGACTACCTGTAAAAGGGTAGCCTCTGAAATTTAGTGGCGGGCTCGCTAGAATTTTGAGCGTAGTAATTTATACCTCGTTTATGAAGACTAGTGATGTTTCCTTTGCCGTAAAACCCCC

>JQ910642 Gnomoniopsis smithogilvyi

CCTCACGGGTGCTACCCAGAAACCCTTTGTGAATTCTTCTCATTGTTGCCTCGGCATGGACTGGCTTCCTATGGAAGTCCCTCTTCTTCGGAAGCGGAGCAGGTCGGCCGGTGGCCCTATAAACTCTTTGTTTTTACAGTGTATCTTCTGAGTAAACAACTATAAATGAATCAAAACTTTTAACAACGGATCTCTTGGTTCTGGCATCGATGAAGAACGCAGCGAAATGCGATAAGTAATGTGAATTGCAGAATTCAGTGAATCATCGAATCTTTGAACGCACATTGCGCCCGCTGGTATTCCAGCGGGCATGCCTGTTCGAGCGTCATTTCAACCCTCAAAACTTCGGTTTTGGTGTTGGAGGAATACTTGTAAAAGAGTACCCTCTGAAATTTAGTGGCGGGCTCGCTAGAATTTTGAGCGTAGTAATTTTATACCTCGTTTATAAAGACTGGCGGTTGCTCTTGCCGTAAA

Gnomoniopsis（TEF1）

>XP1-6

GGTAAGTTGTCATCTCCCCATCATCAAGATCATCATTGATGATGRRATCTACACCATATCTGCCCCTCAGTCACAGCTGCGATCGTAAGGTGGCGGCTGTTCTCCTCGGGGCGCAGTGGCCCGCCTTTCTTTTTTCTCGCCTACTGCACATTTTTTCCTCTGAGTGGTGCGGGGTTTGGTTCTCTTATCTGGGAGATAGACAAACGTGACCCACCTACCATCCTCCACTGCCCCTCCGTCATCTTCCATCCATCCTGAGCCAATGTATTGTCGCTGCCCTCCCTACACAAAAGTAATGACCTCGTTGCTGACGCAATTCTCATCACAGCTGCTGAGC

>TMR3-3

GTTCGAGAAGGAGGGTAAGTTGTCATCTCCCCATCATCAAGATCATCATTGATGATGAAATCTACACCATATCTGCCCCTCAGTCACAGCTGCGATCGTAAGGTGGCGGCTGTTCTCCTCGGGGCGCAGTGGCCCGCCTTTCTTTTTTCTCGCCTACTGCACATTTTTTCCTCTGAGTGGTGCGGGGTTTGGTTCTCTTATCTGGGAGATAGACAAACGTGACCCACCTACCATCCTCCACTGCCCCTCCGTCATCTTCCATCCATCCTGAGCCAATGTATTGTCGCTGCCCTCCCTACACAAAAGTAATGACCTCGTTGCTGACGCAATTCTCATCACAGCTGCTGAGCTCGGTAAGGGTT

>OP688537 Gnomoniopsis rosae GUCC 408.17

TCATCGAGAAGTTCGAGAAGGAGGGTAAGTTGTCATCTCCCCATCATCAAGATCATCATTGATGATGAAATCTACACCATATCTGCCCCTCAGTCACAGCTGCGATCGTAAGGTGGCGGCTGTTCTCCTCGGGGCGCAGTGGCCCGCCTTTCTTTTTTCTCGCCTACTGCACATTTTTTCCTCTGAGTGGTGCGGGGTTTGGTTCTCTTATCTGGGAGATAGACAAACGTGACCCACCTACCATCCTCCACTGCCCCTCCGTCATCTTCCATCCATCCTGAGCCAATGTATTGTCGCTGCCCTCCCTACACAAAAGTAATGACCTCGTTGCTGACGCAATTCTCATCACAGCTGCTGAGCTCGGTAAGGGTTCCTTCAAGTA

>OP688536 Gnomoniopsis rosae GUCC 408.7

CGAGAAGGAGGGTAAGTTGTCATCTCCCCATCATCAAGATCATCATTGATGATGRRATCTACACCATATCTGCCCCTCAGTCACAGCTGCGATCGTAAGGTGGCGGCTGTTCTCCTCGGGGCGCAGTGGCCCGCCTTTCTTTTTTCTCGCCTACTGCACATTTTTTCCTCTGAGTGGTGCGGGGTTTGGTTCTCTTATCTGGGAGATAGACAAACGTGACCCACCTACCATCCTCCACTGCCCCTCCGTCATCTTCCATCCATCCTGAGCCAATGTATTGTCGCTGCCCTCCCTACACAAAAGTAATGACCTCGTTGCTGACGCAATTCTCATCACAGCTGCTGAGCTCG

GTAAGGG

>DQ313565 Apiognomonia errabunda AR 2813

CATCGAGAAGTTCGAGAAGGAGGGTAAGTCACAATCTCCCAAGCTTCACAGACCTCTACACATCCCGAACGTCATAAGCGAGTTGCTGCAATTGCAAGATTGCGGCTGTGTGCTGTGGCGGGGCGGCTCTTTTTTCGCCCACTGCACATTTTTCGCTTTGGTGGTGCGGGGTTCGCCCGCCTCTTATCACCGACCAACGTGACCCACCACCAAAGCACCTCAGCACTCACGCACACATCACCGCCCTCAATCACTTTCTTGCTGCTCAACTGGCAATCATCATCATGCTAACTCAATTTCCCTCACAGCTGCTGAGCTCGGAAAGGGTTCTTTCAAGTAC

>GU320801 Gnomoniopsis alderdunense CBS 125680

CACAATCTCACCATCAATCCGATCTCCTTGATCATCGAAACCATCTCATCGCAGACAACCCTCGAACCTCAAGCTGCGACCGCAAGGTTGCGGCTGTCTGCTCGTGGGGCTGAGTGGGCTCGTCTTTTTTCGCCCTGTGCACAATTTTCGTTTTCGGTGGTGCGGGGTTTGTCGCTCTTATCTGGGAGATAGACAAACGTGACCCACCCCTGCGTTACTCAAGAGGGGCCATCTCGGCCAACCTCTACCCACCTTGACAGCCCGTGCCTATGCTCTCCTTCCCTTGGCCAATGAACCATGATACCGATGCTAACTCGATTGTTATCGCAGCTGCTGAGCTCGGAAAGGGTTCTTTCAAGTATGCCTGGGTTCTGGACAAGCTGAAGGCTGAGCGTGAGCGTGGAATCACCATCGACATTGCTCTCTGGAAGTTCGAGACTCCCAGGTACTACGTCACCGTCATTGGTAAGCTAGCCCCCATCATTGGCCATATCGTGCAGGTTCGACAGTCTTGCTGCCTGAACGCGCGCGATAGTGGTTTTGGCAATCACTTGATTTCACAACATCCGCTGACGCCTTGATTCTACAGACGCTCCCGGTCACCGTGATTTCATCAAGAACATGATCACTGGTACCTCCCAGGCCGACTGCGCTGTTCTCATCATTGCCTCCGGTACTGGTGAGTTCGAGGCTGGTATCTCCAAGGATGGCCAGACTCGTGAGCACGCTCTGCTCGCCTACACCCTCGGTGTCAAGCAGCTCATTGTTGCTTGCAACAAGATGGACACTGCCGAGTGGAAGCAGGCCCGTTTCGAGGAGATCCAGAAGGAGACCTCTACCTTCATCAAGAAGGTTGGCTACAACCCCAAGACCGTTGCCTTTGTCCCCATCTCTGGCTTCAACGGTGACAACATGATCGAGGGTGAGACTCTCGACCCCCGTGCCAAGGCCTGGTACAAGGGCTGGAAGAAGCTGGGTTCCGACGGCAAGGAGGTCTCTGGAAAGACCCTCCTCGACGCCATTGACGCCATTGAGCCCCCCAAGCGTCCCACCGACAAGCCCCTCCGTCTTCCCCTCC

>MN605519.1 Gnomoniopsis daii strain CMF002A

CATAAAATACCTCAGAGTGCTGCTGCTGCGACCGCAAGGTCGTGGCCGTTGTTCTGTTGAGGGGCGAAGTGGCTCGTCTTTTTTTCGCCTACTGCACATTTTTCGCTTTCGGTGGTGCGGGGTTTGTCCCTCTTATCTGGGAGATAGACAAACGTGACCCACCCTTGGTCACTCAATGGTCCTCTCGATCATCCTCCACCACCTCAACAGTCCTTACCCACGCCTCCGTCTTCTTTCTTGTAAACAATGATCATCTCGATGCTAACTCGATTATCATCACAGCTGCTGAGCTCGGTAAGGGTCCCTTCAAGTAA

>MZ936389 Gnomoniopsis fagacearum CFCC 54288

CATCACAAACTCCTCGAGAGCGCCCACAGCTGCGACCGCGAGGTTGCGGCCGCTTCCTTGGGGCAGAGTGGCTCTCTTTTTTCGCCTGCTGCACATTTTTCGTTTTCAGTGGTGCGGGGTTTGTCGCTCTCTTATCTGGGAGACAGATAAACGTGACCCACTCCTCGTCCCTCACTCGCCATCTCGGCCAACCTCCATTCACCCTCAACAGCCCATGACTCTTATCCTCTCTTCCCTTCTGCTCAATCAACGATCATCATGATGCTAACTCAATCGCCACCACAGCTGCTGAGCTCGGAAAGGGTTCCTTCAAGTAC

>MZ936394 Gnomoniopsis guangdongensis CFCC 54443

CATCAAAAATCACCTCAAACCGCTGCTACTGCGACCGCATGGTCGTGGCCATTGGCCTTTTGGGGGCGCAGTGGCTCGTCTTTTTTCGCCCACTGCACATTTTTCGTTTTCGGTGGTGCGGGGTTTGTCCCTCTTATCTAGGAGATAGACAAACGTGACCCACCCTGGGTCACTCAATGGTCCCCTCGGCCATCCTCCATTCATCTCAACAACCCATACCCATATCCTTGTCATATCATACCATGGAGTTTTCAATGCTAATTCGAATGTCATAACAGCTGCTGAGCTCGGAAAGGGTTCTTTCAAGTAC

>MZ936397 Gnomoniopsis hainanensis CFCC 54376

CATCATCAAGACTGGAAATGCTGCGATCGCGAGTCGCGGGCGTTTGCTCTCAGCGGAGTGGCTCTTTTTTTTCACCTACTGCACATTTTTCGTTTTCAGTGGTGCGGGGTTTGTTGCCTCTTATCTGGGAGATAGACAAACGTGACCCACTCCTCGTCACTCCCCCCATCAACTTTAACAACCCATGCCCATCCCGTCCTGTCCGTACTCATTCAATGATTACATGATACTAACTAAAGCATCATCACAGCTGCTGAGCTCGGAAAGGGTTCTTTCAAGTAT

>MZ936398 Gnomoniopsis hainanensis 55877

CATCATCAAGACTGGAAATGCTGCGATCGCGAGTCGCGGGCGTTTGCTCTCAGCGGAGTGGCTCTTTTTTTTCACCTACTGCACATTTTTCGTTTTCAGTGGTGCGGGGTTTGTTGCCTCTTATCTGGGAGATAGACAAACGTGACCCACTCCTCGTCACTCCCCCCATCAACTTTAACAACCCATGCCCATCCCGTCCTGTCCGTACTCATTCAATGATTACATGATACTAACTAAAGCATCATCACAGCTGCTGAGCTCGGAAAGGGTTCTTTCAAGTAT

>GU320803 Gnomoniopsis racemula CBS 121469

CACAATCTCCCCATTAATCCGATCTCTTTGATCATCGAAATCACATCGAAAACACTCTGGAGCCGCAGCTGCGACCGCAAGGTAGCGGCTGTCTGCTTGGGGCTTGCGTGGCTCGACTTTTTTCGCCCTGTGCACATTTTTCGTTTTCGGTGGTGCGGGGTTTGTCGCTCTTATCGGGCGATAGACAAACGTGACCCACCCTGCGTCACTCAAAGGGGCCCTCTTGGCCAACCTCCACCCACCTCGACAGCCCATGCCTTCACACTCGTTCTCTTGCTCAATGAACAATGATACCGATGCTAACTCAATTGTTATCACAGCTGCTGAGCTCGGAAAGGGTTCTTTCAAGTATGCCTGGGTTTTGGACAAGCTGAAGGCTGAGCGTGAGCGTGGAATCACAATCGACATTGCTCTCTGGAAGTTCGAGACTCCCAAGTACTACGTCACCGTCATCGGTAAGTTAGCAAGTTTTCCCCCAACACTGGCAATGTCGTGCAGGTTCGGCAGTGTTGCTGCCCGACCGCGCGCGATGCCAAACTGGGCAATTCCTTGACTTGGCGACATGTGCTGATGCCTTGACTCCAGACGCTCCCGGTCACCGTGATTTCATCAAGAACATGATCACTGGTACCTCCCAGGCCGACTGCGCCGTTCTCATCATTGCCTCCGGTACTGGTGAGTTCGAGGCTGGTATCTCCAAGGATGGCCAGACTCGTGAGCACGCTCTGCTCGCCTACACCCTCGGTGTCAAGCAGCTCATTGTTGCTTGCAACAAAATGGACACTGCCGAGTGGAAACAGGCTCGTTTTGAGGAGATCCAGAAGGAGACCTCCACCTTCATCAAGAAGGTCGGCTACAACCCCAAGACCGTTGCCTTCGTTCCCATCTCTGGCTTCAACGGTGACAACATGATCGAGGGCGAGACTCTCGACCCCCGTGCCAAGGCCTGGTACAAGGGCTGGAAGAAGCTGGGTTCCGACGGCAAGGAGGTCTCTGGAAAGACCCTCCTCGACGCCATTGACGCCATTGAACCCCCCAAGCGTCCCACCGACAAGCCCCTCCGTCTTCCCCTCC

>MZ936399 Gnomoniopsis rossmaniae CFCC 54307

ACCTCGCAAACACCCCCGAGCGGGTGGTCGCGGTCGCGAGACTGCGGCCGTTTGCTCTGGGCCGAGAGGCCCTTTTTTTTTTTGGCCCACTGCACATTTTTCGTTTTCCGTGGTGCGGGGTTTGTCGCTCTTATCTGCGAGATAGACAAACGTGACCCACTTCTCATCCCTCCTCAGTCGCCGCCTCGGCCATCCTCCACCGCCCACCTCAACAGCCCATGCCCACAATCCCCCTTCTCTTGCCAATAAATGACCATCTCGATGCTAACTCAATTGCCTTCGCACAGCTGCTGAGCTCGGAAAGGGTTCCTTCAAGTAC

>MZ936400 Gnomoniopsis rossmaniae CFCC 55876

ACCTCGCAAACACCCCCGAGCGGGTGGTCGCGGTCGCGAGACTGCGGCCGTTTGCTCTGGGCCGAGAGGCCCTTTTTTTTTTTGGCCCACTGCACATTTTTCGTTTTCCGTGGTGCGGGGTTTGTCGCTCTTATCTGCGAGATAGACAAACGTGACCCACTTCTCATCCCTCCTCAGTCGCCGCCTCGGCCATCCTCCACCGCCCACCTCAACAGCCCATGCCCACAATCCCCCTTCTCTTGCCAATAAATGACCATCTCGATGCTAACTCAATTGCCTTCGCACAGCTGCTGAGCTCGGAAAGGGTTCCTTCAAGTAC

>GU320805 Gnomoniopsis sanguisorbae CBS 858.79

CACTATCTCCCCATCGTCCTGATCTCCTTGATCATCCAAATCATCACATTTGCAAAAACCCTAGAGCCCCAGCTGCAACCACAAGGTGGTGGCTGGTTGGGGTTGAGTGGCTCGTCTTTTTTGGCCCTGTGCACATTTTTTCGTTTTTTAGCGGTGCGGGGTTTGTCTCTCTTATCTAGGAGATAGACAAACGTGACCCACCCCTCCGTCACCCGAAGGACTATCTTGGCCAGCCTTCACCACCTGAACAGCTAATGCCTACACCTCCTTGCCTTGATGAATGAATCATGATATCGATGCTAACTGACTGTCATAACAGCTGCTGAGCTCGGAAAGGGTTCTTTCAAGTATGCCTGGGTTCTGGACAAGCTGAAGGCTGAGCGTGAGCGTGGAATCACCATCGACATTGCTCTCTGGAAGTTCGAGACTCCCAAGTACTACGTCACCGTCATTGGTAAGTTAGCAATTATACTTTCCCCCGTTGACTGGCCATGCCGTGCTGGGTTTGGCAGTATCACTGCTTCTCCCGCGCGATGCTGGCTTTAGCAATCCCTCGACTTCACAACACACGCTGACGCCTCGGTTTACAGACGCTCCCGGTCACCGTGATTTCATCAAGAACATGATCACGGGTACTTCCCAGGCCGAGTGCGCTGTTCTCATCATTGCCTCCGGTACTGGTGAGTTCGAGGCTGGTATCTCCAAGGATGGCCAGACTCGTGAGCACGCTCTGCTCGCTTACACCCTCGGTGTCAAGCAGCTCATTGTTGCTTGCAACAAGATGGACACTGCCGAGTGGAAGCAGGCCCGTTTCGAGGAGATCCAGAAGGAGACCTCCACCTTCATCAAGAAGGTCGGCTACAACCCCAAGACCGTTGCCTTTGTCCCCATCTCCGGCTTCAACGGTGACAACATGATCGAGGGCGAGTCTCTCGACCCCCGTGCCAAGGCCTGGTACAAGGGCTGGAAGAAGCTCGGTTCCGACGGCAAGGAGGTCTCTGGAAAGACCCTCCTCGACGCCATTGACGCCATTGAGCCCCCCAAGCGTCCCACCGACAAGCCCCTCCGTCTCCCCCTCC

>MZ936401 Gnomoniopsis silvicola CFCC 54304

CATCACCAATACCTCCAAGTGCTGCTGCTGCGACCGGAAGGTCGTAGCCGTTGCCCTGGCGAGGGGCGAAGTGGCTCGTCATTTTTTCGCCTGTTGCACATTTTTCGTTTTCGGTGGTGCGGGGTTTGTCCCTCTTATCTGGGAGATAGCCAAACGTGACCCACCCTGGGTCACTCGATGGTCATCTCGGCCATCCTCCACCACGCTTAACAGCCCTTATCCACATTTCCATCTTCTTTCTTGTATACAATGATGATCTCGATGCTGACTCGATTATCATCATAGCTGCTGAGCTCGGAAAGGGTTCCTTCAAGTAC

>MZ936402 Gnomoniopsis silvicola CFCC 54418

CATCACCAATACCTCCAAGTGCTGCTGCTGCGACCGGAAGGTCGTAGCCGTTGCCCTGGCGAGGGGCGAAGTGGCTCGTCATTTTTTCGCCTGTTGCACATTTTTCGTTTTCGGTGGTGCGGGGTTTGTCCCTCTTATCTGGGAGATAGCCAAACGTGACCCACCCTGGGTCACTCGATGGTCATCTCGGCCATCCTCCACCACGCTTAACAGCCCTTATCCACATTTCCATCTTCTTTCTTGTATACAATGATGATCTCGATGCTGACTCGATTATCATCATAGCTGCTGAGCTCGGAAAGGGTTCCTTCAAGTAC

>KR072534 Gnomoniopsis smithogilvyi CBS 130190

TCTTCATCGTCGATTCCTTGTACAAGCATCCCCGGATCCAAAGCTGCGAGCGAGTTGGCCGTTGGCCTTGGCGGGAGTGGCTCGTCTTTTTTCGCCTGCTGCACATTTTTTCGTTTTCGGTGGTGCGGGGTTTGTCGCTCTTATCTGGGAGATAGACAAACGTGACCCACTTTACCGTCACTCCTTGGCCATTTCGGTCACCCCCCATCAACCTCACCAGCCCATGCCTAGAACTCCACCCCATCTTCTTGTTGTCATGATCTCGTCGCTGACTCAATTACTATCATAGCTGCTGAGCTCGGAAAGGGTTCTTTCAAGTACGCCTGGGTTCTGGACAAGCTGAAGGCTGAGCGTGAGCGTGGAATCACCATCGACATTGCTCTCTGGAAGTTCGAGACTCCCAAGTACTATGTCACCGTCATTGGTAAGCTTTCATCCAATCTCCCATCCCGTTGGCCACATCGCGCAGGAACGGCACACGGCTACCTGACCGCGTTTGGTGTTGGTTCCACAGCTCTTCAATTTTGCGGCATTTCACTGACAATATTATTTCTCAGACGCTCCCGGTCACCGTGATTTCATCAAGAACATGATCACTGGTACCTCCCAGGCCGATTGCGCTATTCTCATCATTGCCTCCGGTACTGGTGAGTTCGAGGCTGGTATCTCCAAGGATGGCCAGACTCGTGAGCACGCCCTGCTCGCCTACACCCTCGGTGTCAAGCAGCTCATTGTTGCTTGCAACAAGATGGACACTGCCGAGTGGAAGCAGGCCCGTTTCGAGGAGATCCAGAAGGAGACCTCCACCTTCATCAAGAAGGTCGGCTACAACCCCAAGACCGTTGCCTTCGTCCCCATCTCTGGCTTCAACGGTGACAACATGATCGAGGGTGAGTCCCTCGACGCCCGTGCCAAGGCCTGGTACAAGGGCTGGAAGAAGCTCGGTTCCGACGGCAAGGAGGTCTCTGGAAAGACCCTCCTCGACGCCATTGACGCCATTGAGCCCCCCAAGCGTCCCACCGACAAGCCCCTCCGTCTTCCCCTCCAGGATGTCTACAA

Gnomoniopsis（TUB）

>XP1-6

CAAATGGTGCTGCTTTCTGGTGCGTACTCGAGCTCGACAGCCATATACCTCGACGCGATCTATCCAGCTCGACCGTGTCGCAGCTTCAGTAGCTGACCTCAACCGCAGGCAAACCATCTCTGGCGAGCACGGCCTCGACAGCAATGGCGTGTACGTATATTCCTGTACCGCGGCCAAACACCACGCGACCTGAGCTCAGAACTGATAATTGTACAGTTACAACGGTACCTCCGAGCTCCAGCTCGAGCGTATGAACGTCTACTTCAACGAGGTCAGTCTATTCCAACACCTCGGGACCTCATCTTCGCGACGTTGCTCGCTTTGAATAGGTGGCATCTGACGAGTTTTTCTCCAGGCCTCCGGCAACAAGTACGTTCCTCGTGCCGTCCTCGTCGATCTCGAGCCTGGTACCATGGACGCCGTCCGCGCTGGCCCCTTCGGTCAGCTGTTCCGCCCCGACAACTTCGTCTTCGGCCAGTCCGGTCCCGGAAACAACTGGGCCAAGGGTCACTAACCT

>TMR3-3

AATGGTGCTGCTTTCTGGTGCGTACTCGAGCTCGACAGCCATATACCTCGACGCGATCTATCCAGCTCGACCGTGTCGCAGCTTCAGTAGCTGACCTCAACCGCAGGCAAACCATCTCTGGCGAGCACGGCCTCGACAGCAATGGCGTGTACGTATATTCCTGTACCGCGGCCAAACACCACGCGACCTGAGCTCAGAACTGATAATTGTACAGTTACAACGGTACCTCCGAGCTCCAGCTCGAGCGTATGAACGTCTACTTCAACGAGGTCAGTCTATTCCAACACCTCGGGACCTCATCTTCGCGACGTTGCTCGCTTTGAATAGGTGGCATCTGACGAGTTTTTCTCCAGGCCTCCGGCAACAAGTACGTTCCTCGTGCCGTCCTCGTCGATCTCGAGCCTGGTACCATGGACGCCGTCCGCGCTGGCCCCTTCGGTCAGCTGTTCCGCCCCGACAACTTCGTCTTCGGCCAGTCCGGTCCCGGAAACAACTGGGCCAAGGGTCACTAACCT

>OP688562 Gnomoniopsis rosae strain GUCC 408.17

CTGGTGCGTACTCGAGCTCGACAGCCATATACCTCGACGCGATCTATCCAGCTCGACCGTGTCGCAGCTTCAGTAGCTGACCTCAACCGCAGGCAAACCATCTCTGGCGAGCACGGCCTCGACAGCAATGGCGTGTACGTATATTCCTGTACCGCGGCCAAACACCACGCGACCTGAGCTCAGAACTGATAATTGTACAGTTACAACGGTACCTCCGAGCTCCAGCTCGAGCGTATGAACGTCTACTTCAACGAGGTCAGTCTATTCCAACACCTCGGGACCTCATCTTCGCGACGTTGCTCGCTTTGAATAGGTGGTATCTGACGAGTTTTTCTCCAGGCCTCCGGCAACAAGTACGTTCCTCGTGCCGTCCTCGTCGATCTCGAGCCTGGTACCATGGACGCCGTCCGCGCTGGCCCCTTCGGTCAGCTGTTCCGCCCCGACAACTTCGTCTTCGGCCAGTCCGGTGCCGGAAACAACT

>OP688561 Gnomoniopsis rosae strain GUCC 408.7

CTGGTGCGTACTCGAGCTCGACAGCCATATACCTCGACGCGATCTATCCAGCTCGACCGTGTCGCAGCTTCAGTAGCTGACCTCAACCGCAGGCAAACCATCTCTGGCGAGCACGGCCTCGACAGCAATGGCGTGTACGTATATTCCTGTACCGCGGCCAAACACCACGCGACCTGAGCTCAGAACTGATAATTGTACAGTTACAACGGTACCTCCGAGCTCCAGCTCGAGCGTATGAACGTCTACTTCAACGAGGTCAGTCTATTCCAACACCTCGGGACCTCATCTTCGCGACGTTGCTCGCTTTGAATAGGTGGTATCTGACGAGTTTTTCTCCAGGCCTCCGGCAACAAGTACGTTCCTCGTGCCGTCCTCGTCGATCTCGAGCCTGGTACCATGGACGCCGTCCGCGCTGGCCCCTTCGGTCAGCTGTTCCGCCCCGACAACTTCGTCTTCGGCCAGTCCGGTGCCGGAAACAACT

>DQ862014 Apiognomonia errabunda

TTTCTCACTNGCGGCGCACCAACACTCCCATCGGACGTGACGNCAAGCTGGCCAAGCNTNGTCAACTCCACAACACGCACTGGGGCCTCGTGTGTCNTGCAGAAACACCAGAGGNTCAAGCTTGTGGCCTTGTCAAGAATTTGTCGCTCATGTGCTACGTCAGCGTGGGTTCGCCTGCAGAGCCCATCAAGGAGTTTATGGTACAGCGTAACATGGAGCTGCTCGAAGAGTACGAGCCTGGCTCTAGCCCTGATTCCACGAAGGTCTTCATCAATGGTACATGGGTGGGCGTCCACAATGAGCCCGCTCACCTGGTACAACTTGTCCAAGATCTCCGGCGTCGCTGCATCATCTCGCACGAGGTCTCTCTTGTCCGAGAAATTCGTGACCGCGAGTTCAAGATCTTCTCTGACGCTGGACGTGTGATGCGACCTTTGTTCGTGATCGAGCAACAGGACAACCCCGACACTAACGTACCTCAGGGCACTCTTGCTCTCACGAAGGAACACATCCGCAAACTTGAAGAGGATGCCCTTTACCACCGCAAGANNGACGACGNNGATTATTTTGGATGGGATGGTCTCCAGAACAGCGGTGTCATTGAGTATTTGGACGCTGAGGAGGAGGAGTCGGCCATGATCTGCATGTCTCCTGAGGATCTGGAGGACTACAGGCAGCAGAAGGCCCGAGGAAAGCCTGACCCCAACGTGAAAGTTGAGCCAGAAGATGATGGCAGGAGTCTAAATGCGCGCGTCAAGACCAAGATCAACCTTGACATCCACACGTACACCCACTGTGAGATTCATCCCAGTATGCTTCTAGGGATTTGCGCTAGCATTATTCCCTTCCCAGATCACAATCAGGCAAGTCTGCTCCACCTGAAATTATCATCATGCACACTTACTAACCAAGAA

>GU320787 Gnomoniopsis alderdunense

TGCTGGTGCTGTGCTCGCCTTTTTCGCATCTTCTAGTGCCCCTGAGGGCCTGGACCCCACTATCGCGACCGGCCTCACTTTGGGACTTCGATGCTCCGATATTCACCCTCAGAGACGCGTCCAGATGGCTAACATGATGTTTTCTTCGCATATAGGTTCACCTCCAGACCGGCCAATGTGTACGTACCCCGTACCCCGCCGTCGTCAAGAATACCTCTGTCGCTCTTCGCTGGGTAGGCCTCGACATGACACACTGACACGTTTCCCAGGGTAACCAAATTGGTGCTGCCTTCTGGTGCGTACCCGAACCCCACGATCATGTTTTACGACGCGACAAGGTCATCTCGAACGTGTTGTGGCTTCCATGACTGACCTCGTCTGCAGGCAAACCATCTCTGGCGAGCACGGCCTCGACAGCAATGGCGTGTATGTACATCATAACCCCCCCGGCCCGATTCAAGCCCAGAACTGACCACCATGAAGCTACAATGGCACCTCTGAGCTCCAGCTCGAGCGCATGAATGTCTACTTCAACGAGGTATGTCTGTCAAAACCTGGGGTGTCCTCATGGCAAGCCTGCTCGGTTTACTGTGGCGGAAACTGACGCGTTTTCCCTCAAGGCCTCCGGCAACAAGTACGTTCCCCGTGCCGTCCTCGTCGATCTCGAGCCTGGTACCATGGACGCCGTCCGTGCCGGCCCCTTCGGTCAGCTT

>MN605517.1 Gnomoniopsis daii strain CMF002A

TGCTGGCGCTTGGCTCACCTTTTTCGCATCTCCTGCCCCTGAAGCCCTCGACCCCACTATCGCGACCGGCCTCACTTTGGGCCTCGCTCTCGTCTGATATCATTTTCACCAAAAAGACGCGTTTAGATGGCTAACATGATCTTTTTCTTTCGCGTATAGGTTCACCTTCAGACCGGTCAATGCGTACGTATTGGTGACCCCACCGTCGACGACGACGACGACGACACCCTATGCTGCTACAATCGCTAGCTGTCCTCGACACGACAGACTGACACGTTTATTAGGGTAACCAAATTGGTGCTGCCTTCTGGTGCGTACTCGAGCTCCACGACCGTATACCTCGACGCGAAATATACATCTAGACGGTGTCGCGGCTACGAAAAATAGAAGGCTGACCTCGACTACAGGCAAACCATCTCTGGCGAGCACGGCCTCGACAGCAATGGCGTGTACGTACATTTCTACGCCGCGTTACTACGTGGTTCCGGGCTCTTGACTGACTTGCGAACAGTTACAATGGTACCTCAGAGCTCCAGCTCGAGCGTATGAACGTCTACTTCAACGAGGTATGTCTACCAATATCTGGGACAATTCTTGTGCGACTAATCGATTTGCTTGGATGAACTCTGACGCGTTTTCCTTCAGGCCTCCGGCAACAAGTACGTTCCCCGTGCTGTCCTCGTCGATCTCGAGCCCGGTACCATGGACGCCGTCCGTGCCGGTCCTTTCGGTCAGCTT

>MZ936405 Gnomoniopsis sp.

GTAACCAAATTGGTGCTGCCTTCTGGTGCGTACCCGAGCGAGACGACCAATTCCATCGACGCGACTTACCGTCTCGACCGTGTCCATGCTTCAATAACTGACTTCGACTGCAGGCAAACCATCTCTGGCGAGCACGGCCTCGACAGCAATGGCGTGTACGTATCTCTATGCCGCGTACACCACGCGACTTGAGCTCGAAACTGACCGCCGTACAGCTACAACGGTACCTCCGAGCTCCAGCTCGAGCGTATGAACGTCTACTTCAACGAGGTATGTCTGCGATGCACTGGGACGTAGTCATTTGCGACACTGCTTGCTTGGGTTGGAATTGACGCGCTTTCCTTCAGGCCTCCGGCAACAAGTACGTGCCCCGTGCCGTCCTCGTCGATCTCGAGCCTGGTACCATGGACGCCGTCCGCGCCGGCCCCTTCGGTCAGCTGTTCCGCCCCGACAACTTCGTCT

>MZ936410 Gnomoniopsis sp.

GTAACCAAATTGGTGCTGCCTTCTGGTGCGTACTCGAGCTCCACGACCATACTCCTCGACGCGAAACACACATCTAGACGGTGTCGCGGCTACGAAAACACAAGACTGACCTCGACTGCAGGCAAACCATCTCTGGCGAGCACGGCCTCGACAGCAATGGCGTGTACGTTCATTTCTACGCCGCATACGATACGCGACCTTGGGCTCAGGACTGACCTGTGTCTAGCTACAATGGTACCTCGGAACTCCAGCTCGAGCGTATGAACGTCTACTTCAACGAGGTATGTCTACCAATATCTGGGACGTTGTTTTGTGCGACACAGATCGGTTTGCTGGGATGAAATCTGACGCGTTTTCCTCCAGGCCTCGGGCAACAAGTACGTTCCCCGTGCCGTCCTCGTCGATCTCGAGCCCGGCACCATGGACGCCGTCCGCGCCGGTCCTTTCGGTCAGCTTTTCCGCCCCGACAACTTCGTCT

>MZ936413 Gnomoniopsis sp.

GTAACCAAATCGGTGCTGCTTTCTGGTGCGTACCCGACCTCTACGGCCACATCTTTGGACGCGAAATGCCCATCTCGACCGTGTCGTAGCTTCAATTACTGACCTCAACTGCAGGCAAACCATCTCTGGCGAGCACGGCCTCGACAGCAATGGCGTGTATGTATCTTAGGCCACGTGCACCACGCGACTTCACCTCAAAACTAATCGCCGTATAGCTACAACGGTACCTCTGAGCTCCAGCTCGAGCGTATGAACGTCTACTTCAACGAGGTATGTCTAATAATGCCTGGGCCGTCTTGTTTCGCTGCTAGGGTTGGTTCTGACGCGGGTTCCTTCAGGCTTCCGGCAACAAGTACGTTCCCCGTGCCGTCCTCGTCGATCTCGAGCCTGGTACCATGGACGCCGTTCGTGCCGGCCCCTTCGGTCAGCTGTTCCGTCCCGACAACTTTGTCT

>MZ936414 Gnomoniopsis sp.

GTAACCAAATCGGTGCTGCTTTCTGGTGCGTACCCGACCTCTACGGCCACATCTTTGGACGCGAAATGCCCATCTCGACCGTGTCGTAGCTTCAATTACTGACCTCAACTGCAGGCAAACCATCTCTGGCGAGCACGGCCTCGACAGCAATGGCGTGTATGTATCTTAGGCCACGTGCACCACGCGACTTCACCTCAAAACTAATCGCCGTATAGCTACAACGGTACCTCTGAGCTCCAGCTCGAGCGTATGAACGTCTACTTCAACGAGGTATGTCTAATAATGCCTGGGCCGTCTTGTTTCGCTGCTAGGGTTGGTTCTGACGCGGGTTCCTTCAGGCTTCCGGCAACAAGTACGTTCCCCGTGCCGTCCTCGTCGATCTCGAGCCTGGTACCATGGACGCCGTTCGTGCCGGCCCCTTCGGTCAGCTGTTCCGTCCCGACAACTTTGTCT

>EU219125 Gnomoniopsis racemula

GACGCGTCGGCAAATGCTGGCGTTGTGCTCGCCTTTTTTCGCATCTTCTGTGCCCCTGAGGGCCTGGACCCCACTATCGCGACTGGCCTCACTTTGGGACCTTGATCGTCCAATATACACCCCCAGAGACGCGTCTAGATGGCTAACATGATGGTTTCTTCCTTCGCATCTAGGTTCACCTCCAGACCGGTCAATGCGTACGTATTCGTTACCCCGCCGTCGTCAACAATACCTATGTCGCTCTTCGCTGGGAAGGCCTCGACATGACAGACTGACACGTTTCGCAGGGTAACCAAATTGGTGCTGCCTTCTGGTGCGTACCCGAGCCCCACGAGGATGATATTTTTTGACGCGACGAGAACATCTCGAACGTGTTGCGGCTTCGATGACTGACCACGTTTGCAGGCAAACCATCTCTGGCGAGCACGGCCTCGACAGCAATGGCGTGTATGTACATATCTCTCCCTCGGCCGCGAAATACAATGCGATCCCAGCCCTGAACTGACCACCATGAAGTTACAATGGCACCTCTGAGCTCCAGCTCGAGCGCATGAACGTCTACTTCAACGAGGTATGTCTACCAACACCTGGGGTGTCCGCATTGCAAGACTGCTCGGTTTGCTTTAGCGGAAACTGACGCGTTTTCTTTCTTAGGCCTCCGGCAACAAGTACGTTCCCCGTGCCGTCCTCGTCGATCTCGAGCCTGGTACCATGGACGCCGTCCGCGCCGGCCCCTTCGGTCAGCTTTTCCGCCCCG

>MZ936415 Gnomoniopsis sp.

GTAACCAAATCGGTGCTGCCTTCTGGTGTGTCCCCGAGCTCCACGAACATCTTTCTCGACGCGACACACCCGTTTCGACCGTGTCGCGGCTTCGATATCTTCTCGTACTGACCTCGACTGCAGGCAAACCATCTCTGGCGAGCACGGCCTCGACAGCAATGGCGTGTACGTATCTCCATGCCGCAGACACGACGCGACTCGAGCTCAAAACTGACCATCGCATAGCTACAATGGTACCTCCGAGCTCCAGCTCGAGCGTATGAACGTCTACTTCAACGAGGTATGTCTCATCTTTTTGCGATACAGCTCCTTATCTTTGGGGTTCGAATCTGACGCGTTTTCCTGCAGGCCTCTGGCAACAAGTACGTTCCCCGTGCCGTCCTCGTCGATCTCGAGCCCGGTACCATGGACGCCGTCCGCGCCGGCCCCTTCGGTCAGCTGTTCCGCCCCGACAACTTCGTCT

>MZ936416 Gnomoniopsis sp.

GTAACCAAATCGGTGCTGCCTTCTGGTGTGTCCCCGAGCTCCACGAACATCTTTCTCGACGCGACACACCCGTTTCGACCGTGTCGCGGCTTCGATATCTTCTCGTACTGACCTCGACTGCAGGCAAACCATCTCTGGCGAGCACGGCCTCGACAGCAATGGCGTGTACGTATCTCCATGCCGCAGACACGACGCGACTCGAGCTCAAAACTGACCATCGCATAGCTACAATGGTACCTCCGAGCTCCAGCTCGAGCGTATGAACGTCTACTTCAACGAGGTATGTCTCATCTTTTTGCGATACAGCTCCTTATCTTTGGGGTTCGAATCTGACGCGTTTTCCTGCAGGCCTCTGGCAACAAGTACGTTCCCCGTGCCGTCCTCGTCGATCTCGAGCCCGGTACCATGGACGCCGTCCGCGCCGGCCCCTTCGGTCAGCTGTTCCGCCCCGACAACTTCGTCT

>GU320790 Gnomoniopsis sanguisorbae

TGCTGGCGCTATGCTCGCCTTTTTTTCGCATCTTGTGTGCCCCTGAGGGCCTGGACCCCACTATCGCAACCGGCCTCACTTTGGGACCTCGATCGTCCGATATTCACCCCAAGAGACGCGTCGAGATGGCTAACATGATGGTTTCTTGCTTCGCATCTAGGTTCACCTCCAGACCGGTCAATGCGTACGTACCCATTAACCCCGCCGTCGTCAACAATATCCTGCGTCGCTCTACGCTGGGTAGGCTCTCGACATGACGAACTGACACGTTTCGCAGGGTAATCAAATTGGTGCTGCCTTCTGGTGCGTACCCAAGGCCCACCATCATGTTTTTCGACGCGACAGGAACATCTCGAGTGTGTTGCGGCTTCGATAGCTGACCTCGTCTGCAGGCAAACCATCTCTGGCGAGCACGGCCTGGACAGCAATGGCGTGTATGTACAGATACTTCCCTCAGCCGCGAAACACCACGCAATCCAAGCCCAGAACTGACCACCATGAAGCTACAATGGCACCTCGGAGCTCCAGCTCGAGCGCATGAATGTCTACTTCAACGAGGTATGTCTACCAACACCTGGGGGTGTCCCCATTGCAAAGCTGCCCGGTTTGTTTGGACGGAAACTGACGCGTTTCTCCCTAAGGCCTCCGGCAACAAGTACGTTCCCCGTGCCGTCCTCGTCGATCTCGAGCCCGGTACCATGGACGCCGTCCGCGCCGGCCCCTTTGGCCAGCTT

>MZ936417 Gnomoniopsis sp.

TGGTGCTGCTTTCTGGTGCGTACCCGAGCTCCACGACCGTATTCCTCGACGCGAAATACACATCTAGACGGTGTCGCGGCTACGGAAATAGAAGGCTGACCTCGACTTCAGGCAAACCATCTCTGGCGAGCACGGCCTCGACAGCAATGGCGTGTACGTTCAATTCTATGCCGCATTACTACGCGATTCGGGTCTCGTTGCTGACCTTCGAATAGTTACAATGGTACCTCGGAGCTCCAGCTCGAGCGTATGAACGTCTACTTCAACGAGGTATGTCTGCCAATATATCGGACGGCTTCTTTTGTGATACAGTTCGAAATGCTGGGATGAACTCTGACGCGTCTTCCTGTAGGCCTCCGGCAACAAGTACGTTCCCCGTGCCGTCCTCGTCGATCTCGAGCCCGGTACCATGGACGCCGTCCGCGCCGGTCCTTTTGGCCAGCTTTTCCGCCCCGACAACTTCGTCT

>MZ936418 Gnomoniopsis sp.

TGGTGCTGCTTTCTGGTGCGTACCCGAGCTCCACGACCGTATTCCTCGACGCGAAATACACATCTAGACGGTGTCGCGGCTACGGAAATAGAAGGCTGACCTCGACTTCAGGCAAACCATCTCTGGCGAGCACGGCCTCGACAGCAATGGCGTGTACGTTCAATTCTATGCCGCATTACTACGCGATTCGGGTCTCGTTGCTGACCTTCGAATAGTTACAATGGTACCTCGGAGCTCCAGCTCGAGCGTATGAACGTCTACTTCAACGAGGTATGTCTGCCAATATATCGGACGGCTTCTTTTGTGATACAGTTCGAAATGCTGGGATGAACTCTGACGCGTCTTCCTGTAGGCCTCCGGCAACAAGTACGTTCCCCGTGCCGTCCTCGTCGATCTCGAGCCCGGTACCATGGACGCCGTCCGCGCCGGTCCTTTTGGCCAGCTTTTCCGCCCCGACAACTTCGTCT

>JQ910639 Gnomoniopsis smithogilvyi

CGCGTCGGCAAATGCTGGCCTTTTGCTCACCTATTTTTCGCATCTCCTATGCCCCTGAAGGCCTGGACCCCACTATCGCGACCGGCTTCACTTTGCGGCGTCGATCGTCCGATATCAACCCCTTCAGAGACGCGTCTAAATGGGCTAACATGATGATTTTTCTTCTCTATAGGTTCACCTCCAGACCGGTCAATGCGTACGTACCGAATACCCCCCCCCCGGTCGACGACGACAACAACAACAACAGCAACACTGCTGCTGCGTCACGGTGGCTACATCTCAACATGACAAACTGACATATCTTGCAGGGTAACCAAATTGGTGCTGCCTTCTGGTGCGTACTTGAGCTTCACGTCCATAATCCTCGACGCGAAATGCCCAACTCGACCGTGTCGTGGCTTCGACAACTGACCTCGACTGCAGGCAAACCATCTCCGGCGAGCACGGCCTCGACAGCAATGGCGTGTACGTTTATATATTCCCATGCCGCAAACACCACGCGA
